# PRESCOTT: a population aware, epistatic, and structural model accurately predicts missense effects

**DOI:** 10.1186/s13059-025-03581-y

**Published:** 2025-05-06

**Authors:** Mustafa Tekpinar, Laurent David, Thomas Henry, Alessandra Carbone

**Affiliations:** 1 Department of Computational, Quantitative and Synthetic Biology (CQSB), Sorbonne Université, CNRS, IBPS, UMR 7238, Paris, 75005 France; 2 Centre International de Recherche en Infectiologie (CIRI), Inserm U1111, Université Claude Bernard Lyon 1, CNRS, UMR5308, ENS de Lyon, Univ Lyon, Lyon, 69007 France; 3 Institut Universitaire de France (IUF), Paris, France

## Abstract

Predicting the functional impact of point mutations is a critical challenge in genomics. PRESCOTT reconstructs complete mutational landscapes, identifies mutation-sensitive regions, and categorizes missense variants as benign, pathogenic, or variants of uncertain significance. Leveraging protein sequences, structural models, and population-specific allele frequencies, PRESCOTT surpasses existing methods in classifying ClinVar variants, the ACMG dataset, and over 1800 proteins from the Human Protein Dataset. Its online server facilitates mutation effect predictions for any protein and variant, and includes a database of over 19,000 human proteins, ready for population-specific analyses. Open access to residue-specific scores offers transparency and valuable insights for genomic medicine.

**Supplementary Information:**

The online version contains supplementary material available at 10.1186/s13059-025-03581-y.

## Background

Proteins play a critical role in cellular processes: understanding what proteins do when they interact within complex systems is crucial for unveiling the underlying mechanisms that govern life. Yet, proteins themselves exhibit remarkable complexity, with a single protein potentially having multiple functions depending on their interacting molecules, (alternative) folding, post transcriptional modifications, cellular compartments, or the organisms that it belongs to. Despite advances in experimental and computational methods, only a small fraction of known proteins has been fully functionally characterized [1, 2]. Moreover, current research on the molecular foundations of life and disease has focused primarily on a limited set of well-known proteins, resulting in a bias in protein annotation [3–5]. For the functional characterization of the human proteome, for instance, this bias is massive since there are only 5000 particularly well-studied human proteins [6] over the ~ 20,000 in the full proteome [3, 7, 8]. Thus, given the enormous diversity of proteins and their multiple functions, the understanding of how these proteins recognize other molecules and how sensitive they are to mutations would transform our comprehension of the processes that drive many key biological functions and genetic diseases. Indeed, missense mutations, which are non-synonymous mutations induced by single nucleotide changes, can alter protein function through various mechanisms, such as disrupting protein stability, modulating enzyme activity allosterically, and changing protein–protein interactions (PPIs). Understanding the effects of missense mutations, whether they are pathogenic or benign in the human proteome can help rationalize potential mechanisms of disease-related interface mutations and improve molecular diagnosis. More globally, the identification of the mutations that can affect or not a protein’s action provides a huge amount of information for the design of experiments heading to advances on our comprehension of the functional roles of proteins. Furthermore, pharmaceutical research can be facilitated if protein mutational landscapes can be studied accurately and efficiently. Due to these reasons, numerous clinical, industrial and experimental studies have been conducted. However, a clear comprehension of mutational effects is far from being complete. As a result, new high-throughput experimental and computational methods are highly needed to cover this unmet demand.

Among all human variants, rare missense changes make over 99% of the set of observed missense variants (with a minor allele frequency, in short MAF, below 0.5%), and 90% are extremely rare (with MAF < 10^−6^) [9]. This means that common variants can be more confidently annotated as truly benign and are therefore likely to offer higher labeling accuracy. Hence, the challenge primarily lies in accurately distinguishing between rare pathogenic variants and rare benign polymorphisms, which is crucial in assessing the pathogenicity of missense variations.

In recent years, multiple deep mutational scanning (DMS) experiments for various proteins were conducted by different groups. They demonstrated to experimentally assess the functional impact of nearly all possible missense variants for a target protein. However, these large-scale experiments are not easily implementable nor inexpensive in terms of time and costs. Therefore, highly efficient computational methods are in demand to predict the effects of non-synonymous mutations, and in particular missense mutations.

Many computational methods predicting mutational effects exploit information coming from multiple sequence alignments (MSA) and sequence covariation [10, 11], and more recently deep learning models [12–19]. Here, we are using information coming from three different biological scales: the evolutionary scale, the molecular scale, and the human population scale. We exploit the evolutionary distance between natural sequences which encodes both epistatic information and conservation of amino acids positions [20], and protein structural information coming from available model structures produced by AlphaFold [21, 22] to introduce ESCOTT, an epistatic and structural model of mutational effects. ESCOTT evolutionary- and structure-based predictions are coupled with information on allele frequencies collected in the Genome Aggregation Database (gnomAD) through an intuitive model privileging population-specific allele frequency. This results in a population-aware predictor of mutational effects, named PRESCOTT (Population-awaRe Epistatic and StruCtural mOdel of muTational effecTs). PRESCOTT handles common variants towards their labeling as benign mutations and assigns ESCOTT scores (no change) to rare variants towards their classification in benign and pathogenic. PRESCOTT relies on a few parameters whose thresholds are computed on a small set of a few hundred proteins. No training on human clinical data over large quantities of rare pathogenic and rare benign variants is realized. Hence, PRESCOTT remains interpretable in each step of the computation process towards an understanding of the effect of a mutation.

## Results

**Fig. 1:**
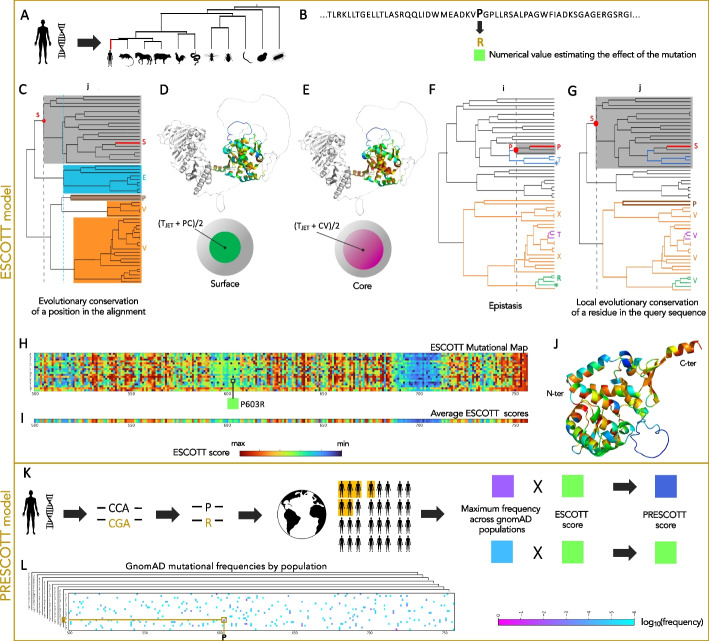
Conceptual building blocks in ESCOTT and PRESCOTT. The ESCOTT model is described in panels **A**–**J** and PRESCOTT in **K**–**L**. **A** Given a protein sequence, for example in humans, ESCOTT examines its homologs and reconstructs a phylogenetic tree of sequences on which it evaluates the positions of the mutation in other species in the tree. The position of the query sequence in the tree is indicated by the red edge. **B** The effect of a mutation, such as P-to-R at position i, will be predicted based on sequences (panels **C**, **F**, and **G**) and on the model structure for the query sequence, if available (panels **D** and **E**). **C** ESCOTT estimates an evolutionary conservation term (T_JET_): for position j in the query sequence, occupied by the amino acid S, ESCOTT considers the level in the tree (dashed line—gray subtree) where the amino acid at that position appeared and remained conserved thereafter. ESCOTT defines the evolutionary conservation [23] of position j by looking at the height of maximal subtrees within the whole tree where the position is conserved, not necessarily with the same amino acid: here, five such maximal subtrees are shown, colored gray, cyan, brown and orange to differentiate amino acids. Two subtrees of maximum height (gray and cyan) are used to set the evolutionary conservation of the position. **D**–**E** Positions 500–756 of the MHL1 human protein are colored on the structural model according to ESCOTT’s model terms, which combine evolutionary conservation (T_JET_), physico-chemical properties (PC), and structural core positions (CV). **F** ESCOTT estimates an epistatic term that evaluates the effect of a mutation P-to-R with the minimal global amount of changes needed to “accept” R within species in the tree. The further the sequences accepting R in the tree (green) are from the reference one (red), the greater the mutational effect. **G** ESCOTT compares sequence positions by favoring residues which are more conserved in the tree as shown for residue S at position j (panel G) vs residue P at position i (panel **F**).** H** ESCOTT mutational map records mutational effects for all the 500–756 positions of the MHL1 human protein. Mutation P-to-R at position 603 is highlighted and the score reported with the corresponding color code. **I** Averages of the scores by columns (across 19 mutations) are reported for positions 500–756 of MHL1. **J** MLH1 structure colored with average scores in **I**. **K** PRESCOTT combines ESCOTT scores and allele frequency in human populations, depending on whether allele frequency is higher than a fixed threshold. **L** Allele frequencies are computed for the eight populations in gnomAD and a PopMax model employed. Each missense mutation is analyzed independently with respect to the eight populations

The genes observed across species today are products of extensive evolutionary processes that selected for functional molecules (Fig. 1A). By modeling the constraints imposed by these processes on protein sequences, we can infer which mutations are likely to be benign and which might be pathogenic, through an analysis that covers every possible substitution at every position (Fig. 1B). ESCOTT exploits the vast array of available sequences and structural models to achieve this. It relies on two key modeling steps: first, assessing the importance of a residue at a specific position within the protein sequence, and second, estimating the mutational effect of a variant at that position.

### ESCOTT models residues in sequences with evolutionary conservation and structural features

ESCOTT models a protein sequence position by integrating two structural features with the evolutionary conservation of the position [23]. The first structural feature focuses on the packing density of amino acids within the protein’s core. This aspect is crucial since mutations that change hydrophobic residues into hydrophilic residues, or those that modify amino acid size, can destabilize the protein, leading to a loss of function [24]. This feature is quantified by the residue’s Circular Variance (CV), which measures the atomic density surrounding a residue, providing insight into its structural position [25, 26]. Lower CV values indicate a protruding residue while higher CV values indicate a buried residue. The second structural feature concerns changes of surface residues, which could impact the protein’s interaction with other molecules, potentially resulting in functional loss [27–29]. This is assessed by the physico-chemical (PC) properties of the residues at an interface, where PC is a metric indicating the propensity of each amino acid to be located at a protein interface [30]. Given the diverse evolutionary and physico-chemical characteristics of protein binding sites, previous studies [23, 25] have shown that combining PC with evolutionary conservation provides a more accurate model for identifying critical residues at interfaces. The concept of evolutionary conservation, called T_JET_ and introduced in [23], is utilized by ESCOTT and illustrated in Fig. 1C. Here, the amino acids S, E, P, and V occur at position j in five maximal subtrees, each maintaining the same amino acid across all species within the subtree. This local residue conservation within the tree, captured by the size of the maximal subtrees conserving a residue at that position, is used in T_JET_ to model the conservation of a position j as a function of the sizes of the associated maximal subtrees. Thus, position j is deemed to be more conserved than position k if the residues in j are associated with larger subtrees than those in k. Therefore, for each residue, ESCOTT combines T_JET_ with the PC and CV metrics to identify (with higher numerical scores) those residues that are conserved or/and located either at the protein surface or within its core (Fig. 1D and 1E) as being more susceptible to bear pathogenic mutations. To achieve this, positions are first categorized in two classes, as either participating to the structure or remaining unstructured (see the “Methods” section). In the first case, the position is modeled by the highest value among two terms, one modeling residues which lie potentially at the interface of the protein with other molecules, and another one modeling residue in the protein structural core:

$$\text{MaxScore}(\text{i})=\text{ Max}\left\{\frac{({\text{T}}_{\text{JET}}^{\text{i}}+{\text{PC}}^{\text{i}})}{2}, \frac{({\text{T}}_{\text{JET}}^{\text{i}}+{\text{CV}}^{\text{i}})}{2}\right\}$$

where i indicates the amino acid position in the protein. The first term in the MaxScore formula models residues that are expected to be evolutionary conserved (${\text{T}}_{\text{JET}}^{\text{i}}$, Fig. 1C) [23] and to satisfy physico-chemical properties at a binding site (PC^i^). The second term models residues that are expected to be evolutionary conserved (${\text{T}}_{\text{JET}}^{\text{i}}$) and to lie at the structural core of the protein (CV^i^). Note that there is no explicit prediction of residues lying at the interfaces but merely a modeling of residues with conserved physico-chemical properties, expected at the interfaces, indicating a potential interaction with other molecules [25]. The maximum between the value of the two terms selects the effective structural role of the position. When a residue in the sequence belongs to a coiled region longer than 5 amino acids, ESCOTT uses only its evolutionary conservation ${\text{T}}_{\text{JET}}^{\text{i}}$ to model it

$$\text{MaxScore}(\text{i})= {\text{T}}_{\text{JET}}^{\text{i}}$$

Based on the intuition that if a residue is evolutionary conserved, it should be important and hence susceptible to mutations. Note that T_JET_, PC and CV values fall in the [0–1] range.

### ESCOTT traces structural information in the course of evolution to predict mutational effects in proteins

Evolutionary processes satisfy two basic hypotheses. First, mutations appearing very rarely in nature are likely deleterious or, in other words, the more a residue is conserved across species, the more sensitive it is to mutations. Second, residues in a protein depend on other residues, because of structural and functional constraints; hence, the effect of a given mutation depends on the presence or absence of other mutations. ESCOTT uses these two hypotheses to model the effect of a mutation, say P-to-R, at position i of the query sequence q by defining a predicted effect (PE) of the mutation as a combination of an independent (Ind) and an epistatic (Epi) term linearly combined with a scalar coefficient

$$\mathrm{PE}\left({\mathrm R}_{\mathrm i}\right)=\left(1-\alpha\right)\;\mathrm{Ind}\;\left({\mathrm R}_{\mathrm i}\right)+\alpha Epi\left({\mathrm R}_{\mathrm i}\right)$$

where $\alpha$ is a linear coefficient set to 0.6 by default (see below for an analysis of the two terms), giving more weight to the epistatic contribution of the model. This model is based on the GEMME approach [20] where the basic terms are fully described.

The independent term, $\text{Ind }({\text{R}}_{\text{i}})$ describes the amino acid frequency observed at a given position i independently from other positions in the sequence. It is computed as the relative frequency of R_i_ (within a reduced alphabet [20]) with respect to P_i_ as follows:

$$\text{Ind}\left({\text{R}}_{\text{i}}\right)= -\text{log}\left[\frac{\text{max}(1,|{\text{S}}_{\text{Ri}}|)}{|{\text{S}}_{\text{Pi}}|}\right]$$

According to this model, the fewer sequences displaying the mutation, the more deleterious the mutation at a given position.

The epistatic term, $\mathrm{Epi }({\mathrm{R}}_{\mathrm{i}})$, captures the epistatic effect of a mutation P-to-R in a query sequence q by measuring the importance of positions that have mutated relative to q in natural sequences s carrying the mutation P-to-R. Intuitively, the idea can be visualized by imagining natural sequences as if they were organized in a distance tree, using two evolutionary measures [20] that capture:

1. The minimal distance in the tree from q to s which accepts R at position i, as illustrated in Fig. 1F. If s is located far from q in the tree, the P-to-R mutation should require a large number of mutations in q for R to be “accepted,” suggesting that P-to-R is likely deleterious.
2. The different level of conservation for residues within the query sequence, as illustrated in Fig. 1G. Mutations in positions that are locally conserved in large maximal subtrees are more impactful than mutations in positions associated with smaller maximal subtrees.

Formally, ESCOTT captures these two concepts by bypassing the use of trees, instead analyzing sets ${\text{S}}_{\text{R}}$ of natural sequences s that carry the mutation R. To address the first concept, it defines a distance function that encodes the importance of each mutated amino acid in s along the sequence, compared to q:

$${\text{D}}_{\text{evol}}\left(\text{q},\text{s}\right)= \sum_{\text{k}=1}^{\text{n}}\text{MaxScore}{(\text{k})}^{2} \times {1}_{{\text{x}}_{\text{k}}^{\text{q}}\ne {\text{x}}_{\text{k}}^{\text{s}}}$$

The importance of each mutated position k is represented by the MaxScore, and the minimal effect across all natural sequences serves as a proxy for the epistatic contribution:

$${\text{PE}}^{\text{EPI}}\left({\text{R}}_{\text{i}}\right)= {\text{min}}_{\text{s}\in {\text{S}}_{{\text{R}}_{\text{i}}}} [{\text{D}}_{\text{evol}}\left(\text{q},\text{s}\right)]$$

This is the foundational term defining $\text{Epi}({\text{R}}_{\text{i}})$. For further details refer to [20].

The second concept is used to calculate a normalized predicted effect (NPE; Eq. 8 in [20]; Fig. 1G) by scaling the PE(R_i_) scores with MaxScore. This ensures that the predicted effects are adjusted relative to the importance of each residue, as follows:

$$\text{NPE}\left({\text{R}}_{\text{i}}\right)=\text{ MaxScore}(\text{i}) \times \text{ PE}({\text{R}}_{\text{i}})$$

The scaling enables a fair comparison of predicted effects across different mutations and residues. For additional details refer to [20].

In summary, ESCOTT incorporates its unique residue representation, MaxScore—which encodes T_JET_, PC, and CV values—into a global epistatic model of mutational effects. It differentiates residues in coiled regions from those in structural regions, with a particular emphasis on residues located on the protein’s surface or core. This intermediate scoring system, built on conceptually well-defined contributions, provides detailed insights into the effects of mutations.

### Analysis of the independent and epistatic terms in ESCOTT

ESCOTT evaluates the mutation from P_i_-to-R_i_ through the contribution of its independent ($\text{Ind}({\text{R}}_{\text{i}})$) and epistatic ($\text{Epi}({\text{R}}_{\text{i}})$) terms. To assess the importance of these contributions, we analyzed ESCOTT’s performance on the ProteinGym dataset [12], consisting of 87 DMS experiments across 72 different proteins and encompassing over 1.5 million single and multiple mutations. ESCOTT achieves an average Spearman correlation of 0.492 on this dataset, compared to 0.459 for the independent term alone and 0.465 for the epistatic term alone. Additional file 1: Fig. S1 provides a detailed breakdown, showing that combining the two terms improves performance for nearly all proteins. Notably, the epistatic model outperforms the independent model on most proteins, a trend also observed in GEMME [20] but more pronounced in ESCOTT. This explains the higher weight (0.6) assigned to the epistatic term in calculating the predictive effect PE.

### PRESCOTT integrates population-specific allele frequencies to filter common variants

Genomic information from unrelated individuals in human populations across the world provides a complementary source of data for assessing the effect of a mutation. Because of its widespread use in clinical diagnosis, this data has been collected, through a community effort across countries, in the Genome Aggregation Database (gnomAD) [31]. gnomAD contains mutation frequencies from unrelated individuals as part of human population genetics studies. It contains data from eight different populations: African/African American, Admixed American, Ashkenazi Jewish, East Asian, European (Finnish), European (non-Finnish), South Asian, and Middle Eastern. This information, when available, is generally used by experts to assess the significance of missense mutations. However, it remains intrinsically limited because allele frequencies depend on the number of individuals sequenced and other factors, which means that simply knowing allele frequencies is an uncertain basis for making decisions.

PRESCOTT combines ESCOTT predictions with population allele frequencies, providing a clinician with an integrated score for missense mutations (Fig. 1K). To do this, PRESCOTT follows the common intuition that the higher the number of individuals carrying the mutation present in gnomAD, the lower the probability that the mutation is pathogenic. If a mutation is too frequent in relation to an overall allele frequency threshold (see the “Methods” section), PRESCOTT adjusts the ESCOTT score by decreasing it proportionally to the allele frequency recorded in one of the eight gnomAD populations, thus reinforcing the benign effect of the mutation. After evaluating a mutation on each population, PRESCOTT selects the popmax allele frequency, defined as the maximum allele frequency in the eight continental populations, as recommended in [32] (Fig. 1L). As a general rule, if a variant is common in one population, it can be assumed to be benign across all populations.

The PRESCOTT model operates using two primary parameters, fine-tuned using sequenced data from gnomAD for a set of 500 human proteins. These parameters include a scaling coefficient, “c,” fixed at 1, and a frequency cutoff, “Fc,” established at 0.0001 (details in the “Methods” section). The model does not apply the frequency cutoff in a straightforward manner to filter variants. This is especially true for variants with a MAF less than 0.005 (0.5%), typically classified as rare. PRESCOTT evaluates all mutations with a frequency value f greater than 0.0001 (as per the testing condition “f > Fc” outlined in the “Methods” section), including those between 0.005 and 0.0001. The model adjusts the ESCOTT score of these mutations by the factor “[Fc − f]/Fc.” Therefore, a mutation with a high ESCOTT value, indicating potential pathogenicity, is less likely to have its clinical assessment altered due to its low frequency. Conversely, for mutations that have lower ESCOTT scores, indicative of benign effects, incorporating allele frequency data reinforces the prediction and leads to the reclassification of certain variants of uncertain significance (VUS) as benign.

**Fig. 2:**
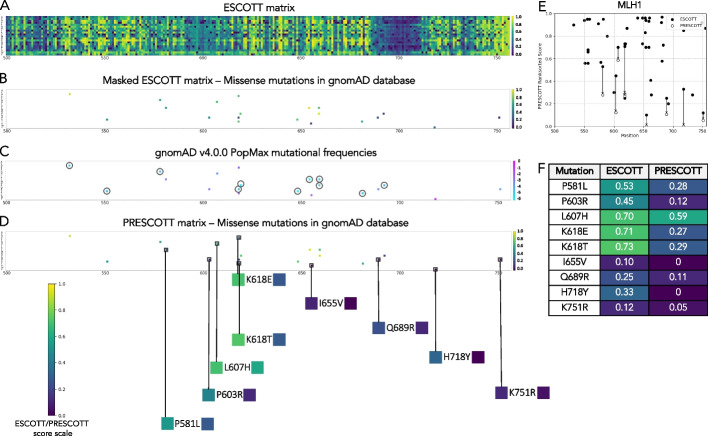
Analysis of the human MLH1 protein with PRESCOTT. The analysis focuses on the 500–756 region of the MLH1 protein, which includes 20 gnomAD missense mutations labeled in ClinVar. ClinVar lists 48 missense mutations in this MLH1 region: 16 benign/likely benign and 32 pathogenic/likely pathogenic. **A** ESCOTT matrix showing all possible substitutions, from any amino acid position in the sequence to any other amino acid. **B** ESCOTT matrix (**A**) masked to highlight scores for the 20 gnomAD missense mutations. **C** gnomAD v.4.0.0 matrix displaying maximum allele frequencies for the 20 mutations across eight populations. Low frequency mutations (cyan tones) are circled. The color scale (pink to cyan) represents high to low frequency. **D** PRESCOTT matrix for the 20 gnomAD mutations in **B**, **C**. Black squares highlight 9 mutations with scores differing from ESCOTT. Below: score changes between ESCOTT (left colored square) and PRESCOTT (right colored square) are represented by colors. See panel **F** for numerical scores. **E** ESCOTT scores (black circles) for the 48 ClinVar missense mutations. Only 45 points are visible due to 3 positions with double mutation: L555P and L555R (Likely pathogenic, ESCOTT score 0.88), L653P and L653R (Likely pathogenic, 0.87), and R659L and R659P (Pathogenic, 0.75). The 20 gnomAD mutations in **B**–**D** are included. PRESCOTT scores (white circles) are lower than ESCOTT scores, as indicated by arrows. Compare to Fig. 7B, 7C. **F** ESCOTT and PRESCOTT scores for the 9 gnomAD mutations in **B**–**D** with changes due to high allele frequency. Colors correspond to the scale in the **A**,** B**, and** D** panels, shown bottom left

Figure 2 illustrates the intermediate steps following the reconstruction of the ESCOTT score matrix in the analysis of the MutL protein Homolog 1, encoded by the *MLH1* gene in the human genome. This gene, involved in DNA repair, is associated with conditions such as Mismatch Repair Cancer Syndrome 1 and Lynch Syndrome 2. Given its high medical relevance, allele frequencies of MLH1 missense mutations are included in gnomAD. For this analysis, we focused on the protein region 500–756 and reconstructed the ESCOTT score matrix, which evaluates the impact of all possible mutations (Fig. 2A). However, genetic disorders are typically linked only to specific missense mutations—those resulting from single nucleotide changes in the DNA sequence. This subset of mutations is much smaller (see Fig. 1L). Within this subset, we further narrow our focus to a smaller group of 20 mutations included in the gnomAD dataset (Fig. 2C). As illustrated in Fig. 2B, we consider only those specific mutations susceptible to ESCOTT score changes and analyzed them with the PRESCOTT model. PRESCOTT modifies the scores for 9 mutations with sufficiently high allele frequencies, while the remaining 11 mutations, associated with lower frequencies, are not adjusted in this process (Fig. 2C, circled mutations). It is crucial to understand that the PRESCOTT model selectively employs gnomAD allele frequency data to modify only those ESCOTT scores associated with sufficiently high allele frequencies, as detailed in the Methods section.

Note that the ESCOTT matrix in Fig. 2A is based on ranksorted scores, ranging from 0 (no mutational effect) to 1 (high mutational effect). Consequently, the ESCOTT and PRESCOTT score scales are identical in this context (Fig. 2, bottom left). In general, ESCOTT generates both a raw score matrix and a ranksorted score matrix, with PRESCOTT adjusting the ranksorted ESCOTT scores within the 0–1 scale (see the “Methods” section).

A key aspect of PRESCOTT's methodology is its independence from ClinVar classifications present in gnomAD, which typically categorize variants as pathogenic, benign, likely pathogenic, or likely benign. In the analyzed MLH1 region, ClinVar describes 48 missense mutations, of which only 20 are included in gnomAD. Figure 2E illustrates how allele frequency data from gnomAD (Fig. 2C) is instrumental to adjust ESCOTT scores (Fig. 2B) into PRESCOTT scores (Fig. 2D and 2F) for the 48 mutations. The 9 ClinVar mutations affected by the score changes are indicated by arrows in Fig. 2E.

To enhance classification, two general thresholds for PRESCOTT scores were established to predict pathogenic, benign, and VUS variants. These thresholds are detailed in the “Classification of human variants as benign, pathogenic, or VUS by ESCOTT and PRESCOTT” section. Using a threshold of 0.28 for benign variants and 0.42 for pathogenic variants, PRESCOTT assists in the classification process (see Fig. 2F). For example, K618E, labeled as “Conflicting classification of pathogenicity” in ClinVar, is reclassified as benign by PRESCOTT (score of 0.27), while L607H, labeled as “Likely benign” in ClinVar, is reclassified as pathogenic by PRESCOTT (score of 0.59). Note that ESCOTT assigns very high scores to both variants, 0.71 and 0.70, respectively. The remaining six mutations receive with very low scores from PRESCOTT, supporting their benign classification. Among these, ClinVar categorizes three as “Benign” (K618 T, Q689R, H718Y), and four as “Likely benign” (P581L, P603R, I655 V, K751R). This analysis highlights how PRESCOTT provides a data-driven approach to variant classification, enabling clinicians to effectively distinguish between pathogenic and benign mutations. Clinicians can further enhance their interpretation by combining PRESCOTT’s final classification with ESCOTT’s intermediate scores—T_JET_, CV, and PC—and structural insights derived from the mutation’s position on the AlphaFold model.

### A large-scale assessment of ESCOTT highlights an accurate identification of sensitive regions in proteins

ESCOTT produces full length sequence evaluations for all possible mutations in a protein sequence. It can be applied to large proteins, intrinsically disordered regions, and splicing variants. The method is sensitive to the long-range correlations between amino acid positions, enabling the detection of differences in the mutational landscape of closely related sequences. We tested ESCOTT’s performance on 32 human protein DMS experiments from the ProteinGym database [12] and assessed its generality across species using all 87 DMS experiments. ESCOTT was benchmarked against three state-of-the-art methods: EVE_ensemble [13], ESM1b [18], and AlphaMissense [19].

**Fig. 3:**
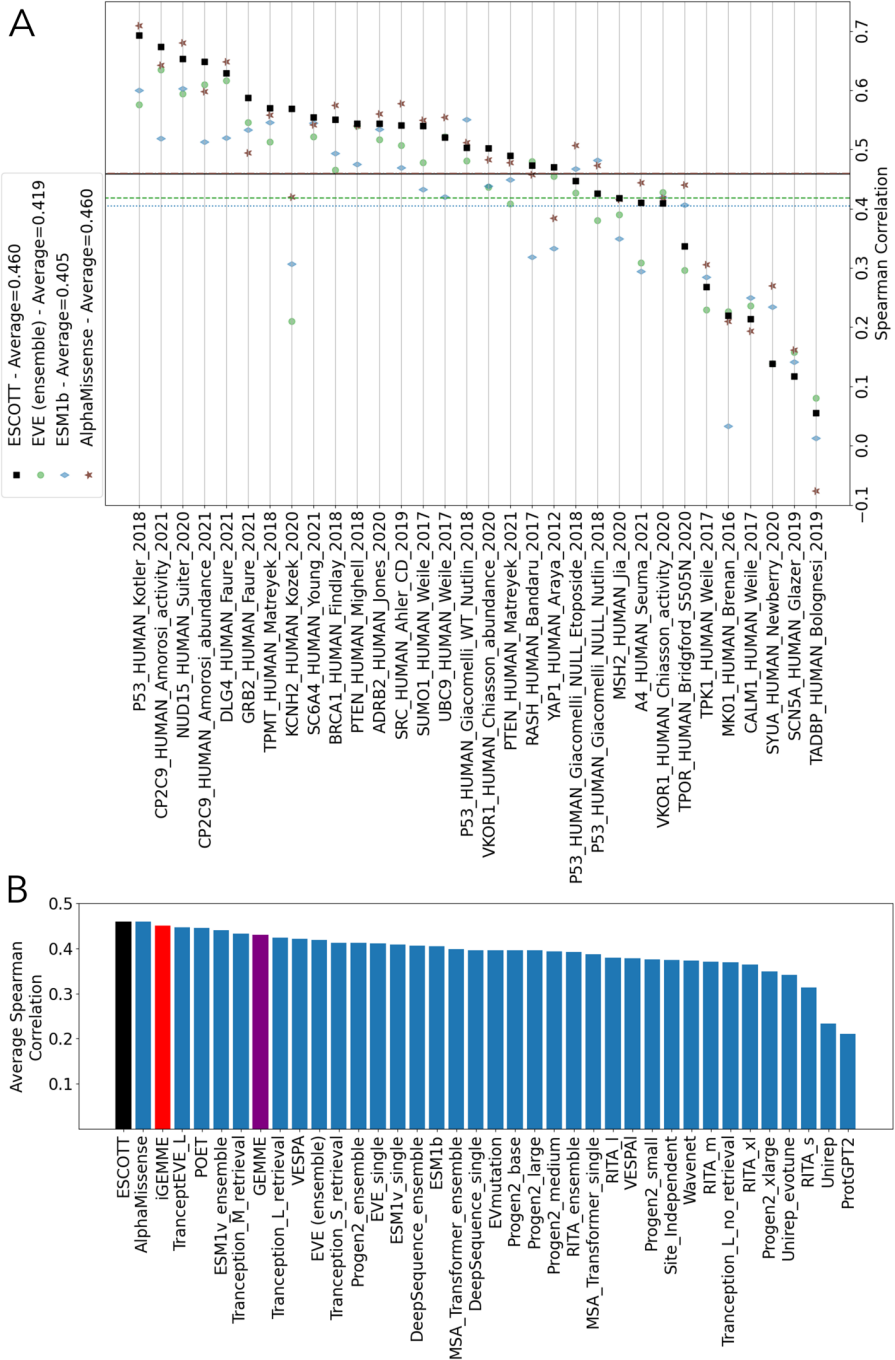
Comparison of ESCOTT, EVE (ensemble), ESM1b and AlphaMissense on 32 DMS experiments for human proteins.** A** ESCOTT (black squares), EVE (ensemble, green circle), ESM1b (blue diamond) and AlphaMissense (brown stars). Dashed lines indicate average Spearman correlation coefficients of the predictive methods with experimental data from the 32 human DMS experiments in the ProteinGym dataset: solid black line for ESCOTT, dashed dotted brown line for AlphaMissense, dashed green line for EVE (ensemble), dotted blue line for ESM1b. **B** Comparison of ESCOTT (black) with existing methods. The list of methods includes EVE (ensemble), AlphaMissense, iGEMME (red), GEMME (purple), and ESM1b. Data for all other methods was obtained from https://www.proteingym.org/substitutions-dms-level, except for AlphaMissense and PoET (ensemble) which are reported in [19] and [33], respectively

The average Spearman correlation between the predictions of the four approaches and the 32 DMS experiments was computed (Fig. 3A, Table 1). ESCOTT achieved the same accuracy as AlphaMissense, with an average Spearman correlation coefficient of 0.46 across 218,301 point mutations (Table 1). A Mann–Whitney paired *u*-test analysis confirms that their performances are statistically indistinguishable (Additional file 2: Table S1 A). Notably, AlphaMissense is fine-tuned on human and primate variant population frequency data, with confidence calibrated on known disease variants. In contrast, ESCOTT relies solely on sequence and structure information. An extended comparison with 37 existing methods, including GEMME [20] and iGEMME, an improved version of GEMME introduced in this work, is shown in Fig. 3B.

**Table 1:** Comparison of ESCOTT with EVE (ensemble), ESM1b and AlphaMissense on human proteins in the ProteinGym dataset. Average Spearman correlation coefficients are reported for all, single and multiple mutation experiments on the 32 human proteins in the ProteinGym dataset. “Average” refers to Spearman correlation computed on the average vector associated to a matrix. For each subset, the best result is in bold and the second best is in italic

Mutational classes	ESCOTT	EVE (ensemble)	ESM1b	AlphaMissense
Overall	**0.460**	0.419	0.405	**0.460**
Single	*0.451*	0.410	0.403	**0.456**
Multiple	**0.524**	0.482	0.420	*0.493*
Average (single)	**0.573**	0.520	0.515	*0.522*

**Fig. 4:**
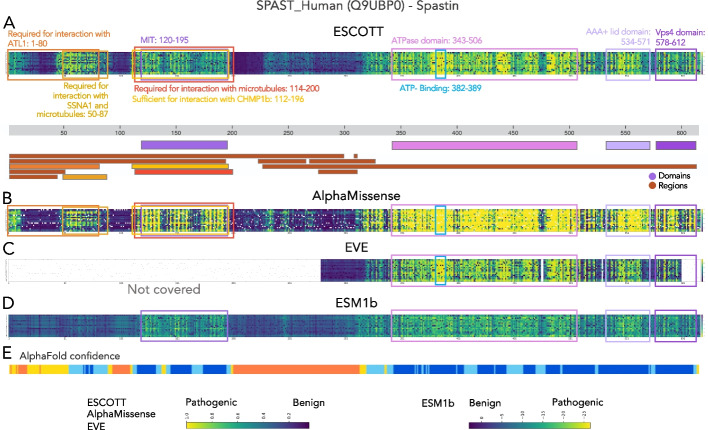
ESCOTT highlights functionally relevant regions of proteins. **A** The human *SPAST* gene, encoding the Spastin protein. ESCOTT evaluation of the full length Spastin protein. The matrix reports ranksorted ESCOTT scores in a colorscale going from dark blue (0, no effect) to yellow (1, highest effect). Below the ESCOTT matrix, known domains (purple color scale) and regions (brown color scale) of interest for the protein are reported from the UniProtKB database (www.uniprot.org/uniprotkb/Q9UBP0/feature-viewer) and the InterPro database (www.ebi.ac.uk/interpro/protein/UniProt/Q9UBP0/), data download in August 2023. Color scale at the bottom. **B** AlphaMissense analysis. Color scale at the bottom. **C**. EVE analysis of Spastin. The four white regions have not been calculated by EVE. They correspond to approximately half of the positions. Color scale at the bottom. **D** ESM1b analysis, as reported in [18]. Color scale at the bottom. **E** AlphaFold confidence (from InterPro) of positions in the Spastin’s structural model. Confidence is represented by a color scale from dark blue (very high) to red (very low)

To gain a deeper understanding of the matrices produced by ESCOTT, EVE_ensemble, ESM1b, and AlphaMissense, we focused on consecutive positions within each matrix where most amino acid mutations exhibit high scores. These elevated scores indicate positions where mutations have a heightened impact on the phenotype measured in the experiment and are potential indicators of pathogenicity. An example of “sensitive” regions in a protein, that is regions which are most sensitive to mutations, is shown in Fig. 4, for the human Spastin protein (encoded in the *SPAST* gene), previously used in [18] to compare ESM1b to EVE. The score matrices for the four computational approaches applied to the human Spastin are displayed in Fig. 4A–D. As remarked in [18], ESM1b highlights one main sensitive region corresponding to the MIT domain (120–195) (Fig. 4D). ESCOTT and AlphaMissense detect several other regions of interest, highlighted in the UniProt [1] and InterPro [34, 35] databases (Fig. 4AB). These are the MIT, ATPase domain (343–506), AAA + lid (534–571) and VPS4 (578–612) domains and three other regions, required for interaction with ATL1 (1–80), for interaction with SSNA1 and microtubules (50–87), and the ATP-binding motif (382–389), along the entire length of the protein. The EVE analysis does not cover approximately half of the total length of the protein, but over the region provided, it highlights known biological signals (Fig. 4C). Interestingly, the N-terminal region of the Spastin protein is marked by relatively high uncertainty by AlphaFold (Fig. 4E) whereas both ESCOTT and AlphaMissense can recognize insightful biological signals there. In general, the regions of a protein reconstructed with higher confidence by AlphaFold align with regions exhibiting high ESCOTT scores.

It should be noted that sensitive regions identified in the ESCOTT matrix are not a direct consequence of the integration of structural information into the model. Indeed, the detection of sensitive regions for the Spastin protein is also present in the iGEMME matrix, which is based solely on evolutionary information, as shown in Additional file 1: Fig. S2. However, the use of structural information improves ESCOTT scores. In addition, the ESCOTT matrix based on ranksorted scores (see the “Methods” section) increases the intensity of the biological signal. Finally, it should be noted that a number of other regions along the protein, such as 437–447 and 470–487 in the ATPase domain, are highlighted by ESCOTT and EVE as being sensitive to mutations, but that no functional interpretation of these regions is known at present.

**Fig. 5:**
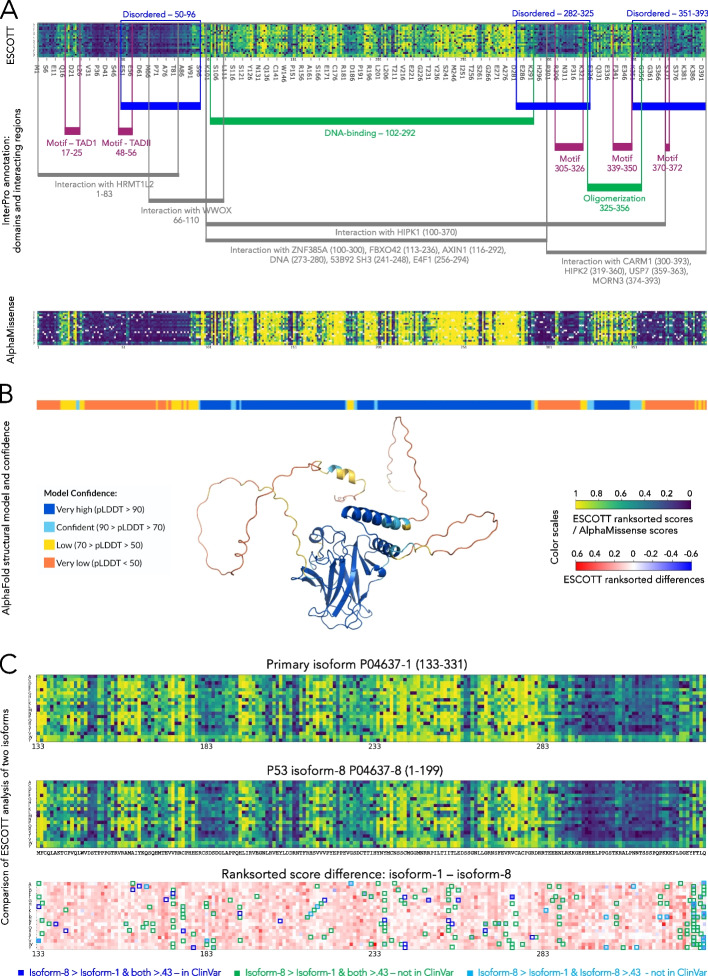
ESCOTT analysis of the P53 human protein. **A** ESCOTT and AlphaMissense matrices of mutational effects, with InterPro annotation of domains and interacting regions highlighting the significance of sensitive regions identified in the matrices. **B** AlfaFold structural model of the *TP53* gene, including confidence scores plotted along the sequence and structural model. The inset legend explains the color scale. **C** Comparative analysis of TP53 “isoform- 8” (199aa; middle) and the corresponding subsequence of the primary isoform, “isoform- 1” (133–331; top). The difference matrix (bottom) highlights mutations with increased effect in isoform- 8: missense mutations from ClinVar predicted to be pathogenic in both isoforms (blue), mutations predicted to be pathogenic in both isoforms but not reported in ClinVar (green), and mutations predicted to be pathogenic only in isoform- 8 and not reported in ClinVar (cyan)

A similar analysis of the well-studied P53 human protein confirms the ability of ESCOTT and AlphaMissense to identify sensitive regions. In Fig. 5A, all InterPro-annotated domains and interacting regions are highlighted as significant by both methods, even in protein segments lacking an associated AlfaFold structural model (Fig. 5B). We considered the three regions of P53 annotated as disordered by InterPro, and as well as larger regions with low pLDDT scores predicted by AlphaFold, to assess the signals identified by the two approaches (Fig. 5AB). Both methods consistently highlight the same regions as potentially sensitive to mutation (light tones for both approaches). These regions include five known motifs annotated by InterPro: TAD I, TAD II, the bipartite nuclear localization signal, the nuclear export signal, the [KR]-[STA]-K motif. All of these motifs are captured by both methods.

**Fig. 6:**
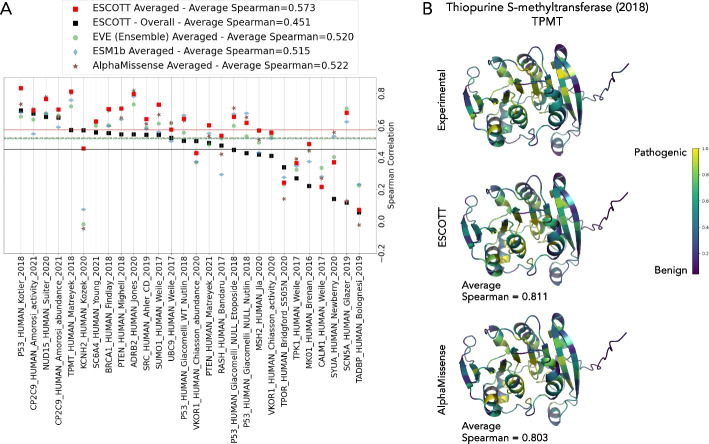
ESCOTT highlights functionally relevant regions of proteins. **A** Spearman correlation coefficients between positional averages of the 28 single point mutation DMS matrices and predicted matrices in ProteinGym. Black squares correspond to the values reported in Fig. 3 for ESCOTT. **B** Averaged scores from experiments, ESCOTT and AlphaMissense are projected onto the human Thiopurine methyltransferase (TPMT_HUMAN in **A**) enzyme. A viridis color scale was used for low (blue) versus high (yellow) average scores projected onto the protein structure. Gray residues (in positions 95, 96, 117, 172) are associated to absence of experimental data. Protein visualizations were realized with PyMOL [36]

To obtain a quantitative assessment of the sensitive regions in a protein, we computed the average of the predictions at each protein position, thus reducing the matrix to a vector of average values (Fig. 1I). Then, we computed the average Spearman’s correlation coefficient of the 28 DMS vectors from single-mutation experiments (Fig. 6A). For almost all proteins and all methods, correlation values increased sharply compared to the correlations computed on DMS matrices (Fig. 3A). This means that all methods are able to identify the most sensitive regions for a protein. ESCOTT obtains an overall average across the 28 experiments of 0.573. This performance surpasses that of AlphaMissense, which scored 0.522, EVE with 0.520, and ESM1b at 0.515. A Mann–Whitney paired *u*-test analysis confirmes the interpretation (see legend in Additional file 2: Table S1B). These average scores can be visually represented on protein structures (Fig. 1J), aiding in the identification of sensitive regions. For instance, Fig. 6B illustrates this with the Thiopurine S-methyltransferase protein (TPMT), where sensitive areas are highlighted in yellow tones. This three-dimensional plotting is instrumental to users in further analyzing proteins, particularly in identifying binding pockets, interaction sites, and functionally relevant residues. In the case of TPMT, both ESCOTT and AlphaMissense exhibit consistency with experimental values. They show comparable correlations in their experimental predictions and consistently identify the same sensitive areas within the protein's structure.

ESCOTT performance has also been evaluated on the entire ProteinGym dataset of 87 DMS experiments of single and multiple point mutations. A comparative account of the four methods on each experiment is shown in Additional file 1: Fig. S3 A, and a more global comparison with 37 state-of-the-art methods [10, 12–17, 37–44] is reported in Additional file 1: Fig. S3B. AlphaMissense outperforms all methods and shows better performance than ESCOTT across species groups, except for humans: 0.564 vs. 0.503 on other eukaryotes, 0.584 vs. 0.553 on prokaryotes, 0.540 vs. 0.474 on viruses, compared to ESCOTT. Yet it is important to note that AlphaMissense advantage partly stems from being explicitly fine-tuned to distinguish between pathogenic and benign effects. In contrast, ESCOTT, along with the other comparative methods, does not incorporate classified data into its modeling process. This difference might offer a margin for further improvement. The incorporation of structural information into the ESCOTT model marks a significant improvement over iGEMME and GEMME. These two models are ranked just below POET [33] and TranceptEVE_L. Thanks to this structural integration, ESCOTT secures the position of second best after AlphaMissense. It achieves a correlation coefficient of 0.492 over the entire ProteinGym dataset, closely following AlphaMissense, which scores 0.525.

A comparative analysis of the positional average vectors for the 76 single point DMS experiments matrices across species is presented in Additional file 1: Fig. S4. Consistent with our observations for human proteins, all methods demonstrate improved predictions for sensitive regions compared to the baseline based on full matrix correlations. The superior performance of AlphaMissense across species groups is reflected in an average Spearman correlation coefficient of 0.614 over the 76 matrices, compared to 0.605 achieved by ESCOTT.

#### Some global observations lead to an improved interpretation of ESCOTT scores

Are sensitive positions located in three-dimensional clusters? An answer to this question would be particularly useful in interpreting protein mutations affecting specific positions or spatial regions in a protein. A visual inspection of the human Thiopurine methyltransferase (TPMT) (Fig. 6B) shows contiguous three-dimensional regions within the protein structure that are sensitive to mutations, suggesting that a protein displays high sensitivity in specific three-dimensional regions and that these regions can be predicted with high accuracy (ESCOTT average Spearman is 0.811 and AlphaMissense is 0.803 on TPMT). In conclusion, ESCOTT scores over amino acid positions are highly informative about the sensitivity of the protein to mutations and allow the identification of sensitive regions along the sequence but also within the structure.

Can ESCOTT better predict phenotypes induced by mutations located in preferred structural elements, such as helices, beta strands, or coils? We evaluated ESCOTT’s predictions of mutations occurring in different structural elements and compared them with experimental data collected at the same locations. The average Spearman correlation on 76 DMS single mutation experiments increases from 0.489 to 0.497 for mutations in structured regions (specifically at positions annotated as secondary structural elements H, E, G, B, I, S, T in DSSP notation). In contrast, for coiled regions (Additional file 1: Fig. S5), the average Spearman correlation drops to 0.450. In conclusion, our analysis suggests that ESCOTT better predicts the effect of mutations located in structured regions. The same analysis was conducted for AlphaMissense, revealing a consistent trend: performance increased from 0.499 in coiled regions to 0.530 in structured regions (Additional file 1: Fig. S5). Using the pLDDT metric, we further evaluated the performance of ESCOTT and AlphaMissense in high confidence (pLDDT > 0.7) and low confidence (pLDDT < 0.7) regions (Additional file 1: Fig. S6). For ESCOTT, the average performance was 0.501 in high confidence regions compared to 0.247 in low confidence regions. AlphaMissense displayed a similar pattern, with scores of 0.54 and 0.264 in high- and low-confidence regions, respectively. As expected, the trend is present but significantly more pronounced with the pLDDT metric. This difference arises because the secondary structure-based metric identifies coiled regions with high pLDDT confidence from AlphaFold, while the pLDDT metric more accurately identifies disordered regions, where predictions of mutational effects are generally less reliable.

Are mutations towards specific amino acids better predicted, regardless of the starting amino acid? To this end, we computed the Spearman correlations for mutations toward a specific amino acid in all single mutational experiments of the ProteinGym dataset. Additional file 1: Fig. S7 shows that ESCOTT predicts mutations toward hydrophilic residues (D, R, E, K, N, Q) particularly well, with the best prediction for positively and negatively charged amino acids D, R, E, K. On the other hand, Spearman’s average reaches the lowest values for hydrophobic residues (I, V, L, Y, F, W, M, C), reducing to 0.330 for C. The predictions of mutations toward neutral residues (G, S, P, T, H, A) (where P is poorly hydrophilic) are consistently better than those for hydrophobic residues. In conclusion, prediction of mutational effects for specific amino acid changes improves accuracy.

### A large-scale assessment of PRESCOTT that uses population-specific counting of allele variants

A major interest in clinical research is to distinguish, in an individual's genome, a rare variant that causes disease from the millions of common and largely benign variants found in every human genome. Today, the gnomAD v4.0 database contains variant data on more than 807,162 individuals. However, a very small part of missense variants are currently observed in gnomAD (estimated to ~ 12% in 2022 [32]), indicating that much larger numbers of individuals will need to be sequenced before the full spectrum of tolerated variants can be discovered [32].

The majority of rare variants are not pathogenic, and current reference population databases have not yet reached saturation for most types of variation [45]. Furthermore, genetic studies often disproportionately represent European populations, leading to an underrepresentation of other communities. This underrepresentation causes individuals from these communities to have a higher number of rare variants with uncertain significance (VUS). Additionally, datasets may include individuals with Mendelian diseases, and despite stringent quality control, sequencing or annotation errors can occur. Given these factors, there is a pressing need for computational methods that are not only interpretable but also capable of providing detailed explanations for mutations suspected to be pathogenic and for regions sensitive to mutations. PRESCOTT has been developed to meet these challenges. It focuses on processing common variants towards categorizing them as benign and minimizes the number of rare variants, specifically evaluating those with allele frequencies between 0.005 and 0.0001. This is achieved by integrating basic allele frequency data, a practice well-established in clinical settings, with the ESCOTT model’s computational predictions that are based on sequence and structure.

**Fig. 7:**
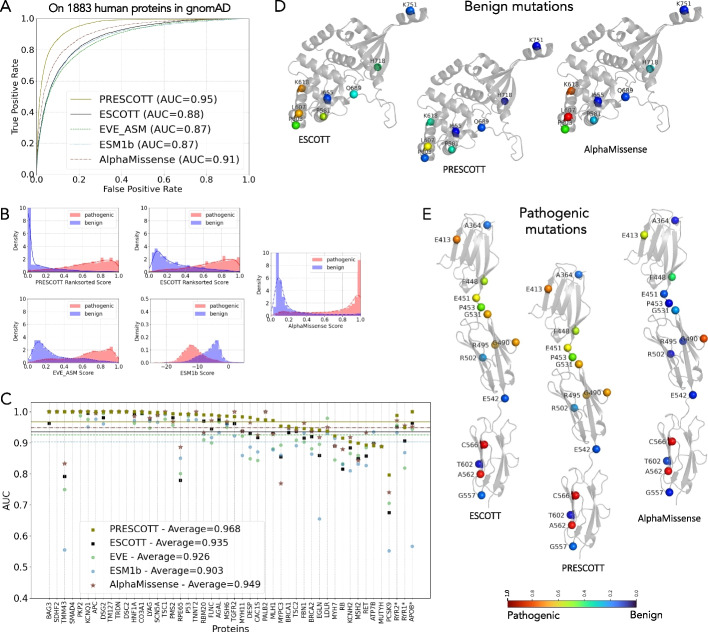
Comparison of PRESCOTT, ESCOTT, EVE, ESM1b and AlphaMissense on human proteins in gnomAD and ACMG datasets. **A** Analysis of 1883 human proteins whose mutations collected in gnomAD v4.0.0 are labeled as pathogenic or benign in ClinVar. AUC curves for PRESCOTT, ESCOTT, EVE, ESM1b and AlphaMissense are superimposed. **B** PRESCOTT, ESCOTT, EVE, ESM1b and AlphaMissense distributions of scores for the 17,230 mutations of the 1883 human proteins. The plots split scores in two disjoint subsets, for mutations labeled as benign (blue) or pathogenic (red) by ClinVar. **C.** Global comparative analysis based on AUC scores for the 48 human proteins in the ACMG v3.1 dataset, for ESCOTT, PRESCOTT, EVE, ESM1b and AlphaMissense. Note that there is a structural model for 45 of the 48 proteins. The three remaining proteins, with a starred name in the figure, RYR2 = 5050 aa, RYR1 = 5122 aa and APOB = 4640 aa, have been evaluated with iGEMME. Note that all 48 proteins are represented by both benign and pathogenic mutations in ClinVar, and that both types of mutation are required to calculate the AUC and make the comparison. All proteins present at least 3 benign mutations in ClinVar. Compare with Additional file 1: Fig. S10 reporting the same analysis on 64 human proteins with at least 1 benign mutation described in ClinVar. **D** ESCOTT, PRESCOTT and AlphaMissense scores of 8 mutations are plot onto the MLH1 structure. These 8 mutations are labeled as benign/likely benign in ClinVar and are listed in Fig. 2G. Due to multiple mutations at position 618, only K618 T is plot. ESCOTT and PRESCOTT scores are reported in Fig. 2G. Color scale is as in Fig. 2. **E** ESCOTT, PRESCOTT and AlphaMissense scores of 14 mutations annotated as pathogenic/likely pathogenic in ClinVar are plotted onto the MYPC3 structure. ESCOTT and PRESCOTT displays show identical scores

We evaluated PRESCOTT's effectiveness in reliably assigning higher scores to pathogenic mutations compared to benign ones. Using a comprehensive dataset of 1883 proteins, as detailed in Table 2 in the “Methods” section, with 7954 pathogenic and 9276 benign mutations from ClinVar, PRESCOTT achieved an AUC of 0.95 without prior knowledge of ClinVar categorization. This performance was benchmarked against other models: AlphaMissense (0.91), EVE (0.87), ESM1b (0.87), and ESCOTT (0.88), as illustrated in Fig. 7A, underscoring the critical role of allele frequency data in refining ESCOTT's scoring system. Moreover, the analysis of the two distinguished ClinVar sets of pathogenic and benign mutations reported in Fig. 7B, clearly show that PRESCOTT model engraves on the ESCOTT distribution of scores for benign mutations (see the “Methods” section for the algorithm, the parameters, and the evaluation of the parameters’ thresholds on an independent dataset of proteins). AlphaMissense also predominantly assigns lower scores to benign mutations, but differs in assigning many low scores to pathogenic mutations. Models like EVE and ESM1b, which do not incorporate allele frequency information, predictably perform closer to ESCOTT. Application of the PRESCOTTscore-high-allele-frequencies algorithm to EVE resulted in a marked improvement elevating EVE's AUC to 0.95.

PRESCOTT’s performance was further analyzed on subsets of the 1883 proteins, grouped by the number of variants per protein (Additional file 1: Fig. S8). Subsets were defined by including at least *k* benign and *k* pathogenic mutations, where *k* = 5,10,15,20. While sets with a higher number of variants and fewer proteins showed a slight decrease in performance, PRESCOTT remained consistently competitive.

Additionally, we conducted an analysis using ClinVar’s starred subsets, which reflect a higher confidence level due to consensus from multiple laboratories. This analysis, shown in Additional file 1: Fig. S9 ABC, aligns well with our primary findings and offers valuable insights into data quality criteria. For each starred subset, we also analyzed the distribution of PRESCOTT scores prior to setting negative values to 0. Additional file 1: Fig. S9D focuses exclusively on mutations whose scores were adjusted by allele frequency in PRESCOTT. Over half of the mutations in each subset were affected, with approximately one-third of all mutations falling within the range [− 1, 0], underscoring the significant impact of allele frequency data on variant classification.

#### PRESCOTT on the ACMG dataset

PRESCOTT has been assessed on the 64 actionable human genes from the American College of Medical Genetics and Genomics dataset (ACMG, version 3.1) [46]. This dataset contains 3790 pathogenic mutations and 1353 benign ones, which were all manually curated and certified by multiple sources in ClinVar. After generating model structures with Colabfold [47–50] and Alphafold [21] (see the “Methods” section), we ran PRESCOTT and compared it to EVE performance as reported in [13], ESMB1b and AlphaMissense. Figure 7C shows that on all ACMG proteins, PRESCOTT achieves an average AUC of 0.968, consistent with its performance on the 1,883 human proteins labeled in gnomAD (Fig. 7A). A Mann–Whitney paired u-test analysis confirms the interpretation (Additional file 2: Table S1 C) (see also Additional file 1: Fig. S10). Figures 7 D and E showcase the application of PRESCOTT on two specific proteins. Figure 7D focuses on a set of ClinVar-classified benign variants of the MLH1 protein (see Fig. 2). Here, we observe notable improvements in the scores assigned by PRESCOTT compared to those by ESCOTT. This is particularly evident in the case of mutations P603, L607, K618 and P581, which are re-evaluated effectively in PRESCOTT thanks to the incorporation of allele frequency data. Conversely, Fig. 7E presents an analysis of 14 ClinVar-identified pathogenic variants in the MYPC3 protein. In this instance, both ESCOTT and PRESCOTT assign identical scores to all mutations, attributable to the low allele frequencies that are not factored in by PRESCOTTscore-high-allele-frequencies algorithm. Notably, some variants like A364, R502, E542, G557, and T602 receive low scores from ESCOTT, indicating a failure to detect their pathogenicity. In contrast, AlphaMissense consistently assigns very low scores to nearly all 14 variants. This results in a lower AUC of 0.769 for the entire MYPC3 protein, as opposed to the significantly higher AUC of 0.954 achieved by PRESCOTT (Fig. 7C).

#### ESCOTT and PRESCOTT intermediate scores to interpret human variants

ESCOTT and PRESCOTT produce a detailed array of intermediate scores for each mutation, which sheds light on the importance of residues from multiple angles: T_JET_, CV, PC. Moreover, the differences between ESCOTT and PRESCOTT scores offer insights into the population-level variations among variants. Pathologists can leverage these four distinct scores to assess mutations’ potential pathogenicity or benignity, aiding in the decision-making process for necessary laboratory tests for loss- or gain-of-function. These intermediate scores play a crucial role in deciphering the mechanisms behind mutations, contributing significantly to the overall evaluation made by both ESCOTT and PRESCOTT.

### Classification of human variants as benign, pathogenic, or VUS by ESCOTT and PRESCOTT

**Fig. 8:**
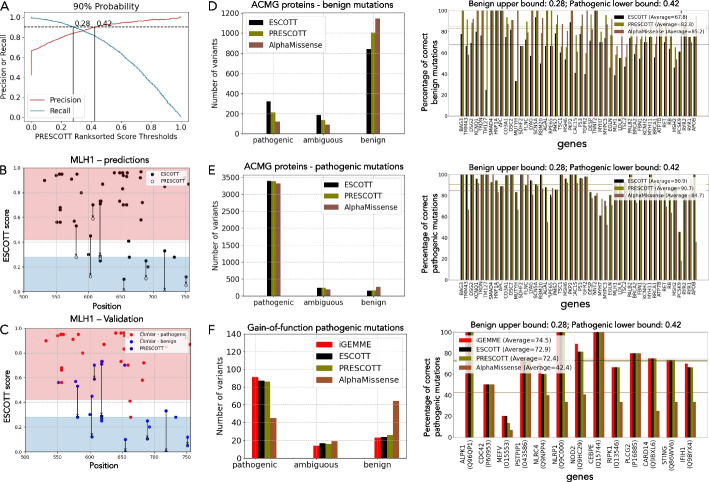
ESCOTT and PRESCOTT benign versus pathogenic mutations. **A** Precision and Recall curves for the 500 human proteins used to calibrate the two thresholds in ESCOTT and PRESCOTT for the purpose of distinguishing between benign and pathogenic variants. **B** Same as Fig. 2E where a colored background indicates those mutations that are labeled as pathogenic (red), benign (blue), VUS (white) variants by ESCOTT/PRESCOTT. **C** The 48 mutations considered in **B** are colored with ClinVar classification as benign/likely benign (16, blue) and pathogenic/likely pathogenic (32, red). Only 45 points are visible, as described in the legend of Fig. 2E. **D** The set of ACMG benign mutations. The plot on the left reports the exact count of mutations which are wrongly predicted as pathogenic (left), or VUS (centre) and correctly as benign (right) by each method. The plot on the right reports, for each protein in the dataset, the percentage of correctly predicted mutations. The horizontal lines indicate the average over all percentages obtained by each method.** E** The set of ACMG pathogenic mutations is analyzed as in **D**. **F** The set of gain-of-function pathogenic mutations involved in autoinflammatory diseases is analyzed as in D. For each gene, the corresponding UniProt ID is provided in parentheses

Recognizing the necessity for automated classification of variants into benign, pathogenic, and VUS, we established two thresholds. These were derived from the analysis of 1849 pathogenic and 2523 benign mutations associated with the 500 human proteins in our training dataset. By examining the precision and recall curves based on their predicted PRESCOTT scores, we identified thresholds that yield 90% precision across these proteins (shown in Fig. 8A). Mutations with scores below 0.28 are categorized as benign, while those above 0.42 are considered pathogenic. Scores falling in between these thresholds are indicative of VUS. In Fig. 8B, we apply these thresholds to the MLH1 protein’s 500–756 region, as seen in Fig. 2E. Notably, several variants shift from the pathogenic (red) to the benign category (blue) in PRESCOTT scores due to allele frequency data. Figure 8C color-codes these mutations according to their ClinVar classifications, revealing that the mutations with lowered PRESCOTT scores align with benign classifications in ClinVar. PRESCOTT’s automated labeling of mutations as benign, pathogenic, or VUS shows reasonable agreement with ClinVar classifications.

In general, PRESCOTT adjusts variant scores exclusively downward due to the inclusion of allele frequency data from gnomAD. Specifically: 1. a variant can only be classified as pathogenic by ESCOTT and subsequently be confirmed as pathogenic by PRESCOTT; 2. a variant classified as pathogenic by ESCOTT may be reclassified as benign or VUS by PRESCOTT. An extensive comparison of ESCOTT/PRESCOTT and AlphaMissense classifications is presented in Fig. 8D and 8E, focusing on the ACMG dataset of human proteins. For the 1353 benign variants, AlphaMissense outperforms PRESCOTT, correctly identifying 1,144 variants compared to PRESCOTT’s 1005, and incorrectly labeling 121 as pathogenic versus PRESCOTT’s 210. However, in the larger pool of 3790 pathogenic mutations, ESCOTT’s performance is superior, accurately predicting 3391 variants compared to AlphaMissense’s 3315, and misclassifying fewer variants as benign (161 versus 276 by AlphaMissense). Intriguingly, ESCOTT’s accurate predictions for pathogenic variants are achieved without relying on allele frequency data. To further underscore this observation, we examined, first, the ability of ESCOTT to distinguish pathogenicity across isoforms and, second, its ability to predict even notoriously challenging mutations, including pathogenic variants from populations with unique genetic heritage and gain-of-function pathogenic variants.

#### ESCOTT/PRESCOTT can differentiate mutational effects across isoforms

Can ESCOTT/PRESCOTT help predict the impact of variants classified as VUS in ClinVar across different isoforms? Variants can be damaging to some isoforms but not others, often due to interactions with alternatively spliced domains. Since ESCOTT evaluates each variant within the context of its corresponding amino acid sequence, driven by its epistatic component, exploring these questions sheds light on its sensitivity to amino acid context. To address this, we conducted an in-depth analysis of isoform- 8 and isoform- 1 (the primary form) of the TP53 human protein, previously analyzed with ESM1b in [18]. Figure 5C highlights all variants in isoform- 8 predicted as pathogenic by ESCOTT, with higher scores in the shorter isoform compared to the primary one. These include both missense mutations reported in ClinVar and unreported mutations. Near the splice junctions of isoform- 8, variants with high scores in both isoforms (blue and green) were identified, alongside variants with increased scores specific to the shorter isoform (cyan). This pattern demonstrates enhanced sensitivity in the shorter form, especially at the right splice junction (positions 326–331). At the left splice junction (positions 133–136), several variants reported in ClinVar (blue) were also observed. These findings align closely with the signals identified by ESM1b at the “borders” of the spliced variant (see Fig. 4 in [18]). However, unlike ESM1b, ESCOTT identifies many score differences throughout the full length of isoform- 8, not just at its borders. A comprehensive list of relevant variants for the isoform- 8 is provided in the supplemental excel file, which includes ESCOTT scores and ClinVar labels (pathogenic, likely pathogenic, benign, likely benign, or VUS). Notably, ESCOTT predicts 32 variants to be pathogenic in at least one isoform, with a score exceeding 0.25—a significant variation given the ranksorted score range of [0–1]. Of these, 17 variants are labeled as VUS in ClinVar (see Additional file 3).

#### ESCOTT and PRESCOTT on unique genetic heritage

PRESCOTT follows the foundational principle observed in gnomAD: high allele frequency in healthy individuals within a population typically indicates the benign nature of a mutation across all populations. However, exceptions to this principle exist, such as in the Finnish population, which possesses a unique genetic heritage resulting from historical isolation and population bottlenecks. These conditions have led to the enrichment of specific pathogenic variants, collectively known as the Finnish Disease Heritage (FDH). FDH encompasses nearly 40 rare monogenic diseases that are over-represented in Finland but remain rare elsewhere [51]. We analyzed the full set of 22 missense mutations reported in FDH (Table 1 in [51]) and compared the performance of ESCOTT, PRESCOTT, and AlphaMissense (Additional file 4: Table S2). ESCOTT systematically identifies pathogenic mutations across all cases except one (in the *CRADD* gene). In contrast, AlphaMissense misclassifies five pathogenic missense mutations as benign (*CRADD*, *BCS1L*, *TWNK*, *HADHA*, and *POLG*), one as VUS (*FSHR*), and does not provide a score for the *CUBN* gene, likely due to the protein’s length. When allele frequency specific to the Finnish population is incorporated into PRESCOTT, we observe significant adjustments to the scores, leading to erroneous evaluations in 10 cases. Based on our findings, we recommend prioritizing ESCOTT scores over PRESCOTT and refraining from population-specific allele frequency adjustments in contexts of unique genetic heritage, such as the Finnish population.

#### ESCOTT, PRESCOTT, iGEMME, and AlphaMissense on recessive and dominant inheritance modes

Recessive and dominant inheritance are the two primary modes through which phenotypes caused by mutations manifest. Most recessive mutations typically result in loss-of-function conditions. In contrast, dominant mutations are more complex, with mechanisms categorized as haploinsufficiency (HI), dominant-negative (DN), or gain-of-function (GF), which includes toxic gain-of-function and constitutive activation. The ESCOTT/PRESCOTT model does not differentiate between modes of inheritance. To illustrate how ESCOTT/PRESCOTT performs across different inheritance modes, we analyzed two genes: the *STAT1* gene, which involves GF, autosomal recessive (AR), and autosomal dominant (AD) mutations (Additional file 5: Table S3; list of mutations from Fig. 4A in [52]), and the *PTPN11* gene, which encompasses GF and DN mutations (Additional file 6: Table S4; list of mutations from Fig. 3A in [52]). These case studies [52] demonstrate that ESCOTT, PRESCOTT, and AlphaMissense face challenges in accurately classifying GF and AD mutations, while achieving perfect classification for DN and AR mutations. Systematic large-scale analyses are required to further evaluate classification performance across inheritance modes.

#### ESCOTT, PRESCOTT, iGEMME, and AlphaMissense on gain-of-function

In our focus on gain-of-function mutations, we assembled a dataset featuring pathogenic missense mutations with gain-of-function, sourced from thirteen human genes associated with autoinflammatory diseases. These genes include *ALPK1*,* CDC42*,* MEFV*,* PSTPIP1*,* NLRC4*,* NLRP1*,* NOD2*,* CEBPE*,* RIPK1*,* PLCG2*,* CARD14*,* STING*, and *IFIH1*. In Fig. 8F, we present a comparative analysis of these mutations, totaling 128, between ESCOTT, PRESCOTT, iGEMME, and AlphaMissense. ESCOTT/PRESCOTT successfully identifies over 72% of these mutations as pathogenic, significantly outperforming AlphaMissense, which classifies only the 42% as pathogenic (Fig. 8F, left side). iGEMME achieves a slightly higher performance, with an average accuracy of 74.5%, suggesting that incorporating structural information into the model may not be critical for scoring gain-of-function mutations. To explore this further, we analyzed the associated T_JET_, PC, and CV distributions for the corresponding 128 positions. As expected, the distributions revealed that gain-of-function mutations tend to occur at highly conserved positions (Additional file 1: Fig. S11 and Additional file 7), consistent with their localization at domain-domain interfaces, as previously reported in [52]. These interfaces are best identified using conservation signals alone. A similar trend was observed in a subset of 102 pathogenic mutations analyzed using EVE and ESM1b, where ESCOTT/PRESCOTT continues to demonstrate strong performance, correctly classifying 71% of the mutations, compared to AlphaMissense at 37%, EVE at 52%, and ESM1b at 49%. The complete list of the 128 mutations, along with the scores assigned by ESCOTT/PRESCOTT, AlphaMissense, iGEMME, ESM1b, and EVE, is available in the supplementary file “gain-of-function-mutations.csv”. Additionally, Fig. 8F (right side) details the predictions of ESCOTT/PRESCOTT, iGEMME and AlphaMissense for each specific protein. Notably, for the ALPK1 protein, which is represented by just two mutations, these mutations are wrongly predicted by AlphaMissense. The analysis of the 15 pathogenic mutations in the *MEFV* protein is particularly intriguing, as these mutations pose significant challenges for ESCOTT/PRESCOTT, iGEMME, and AlphaMissense models.

#### ESCOTT and PRESCOTT versus VARITY, gMVP, and other predictors in analyzing rare mutations

**Fig. 9:**
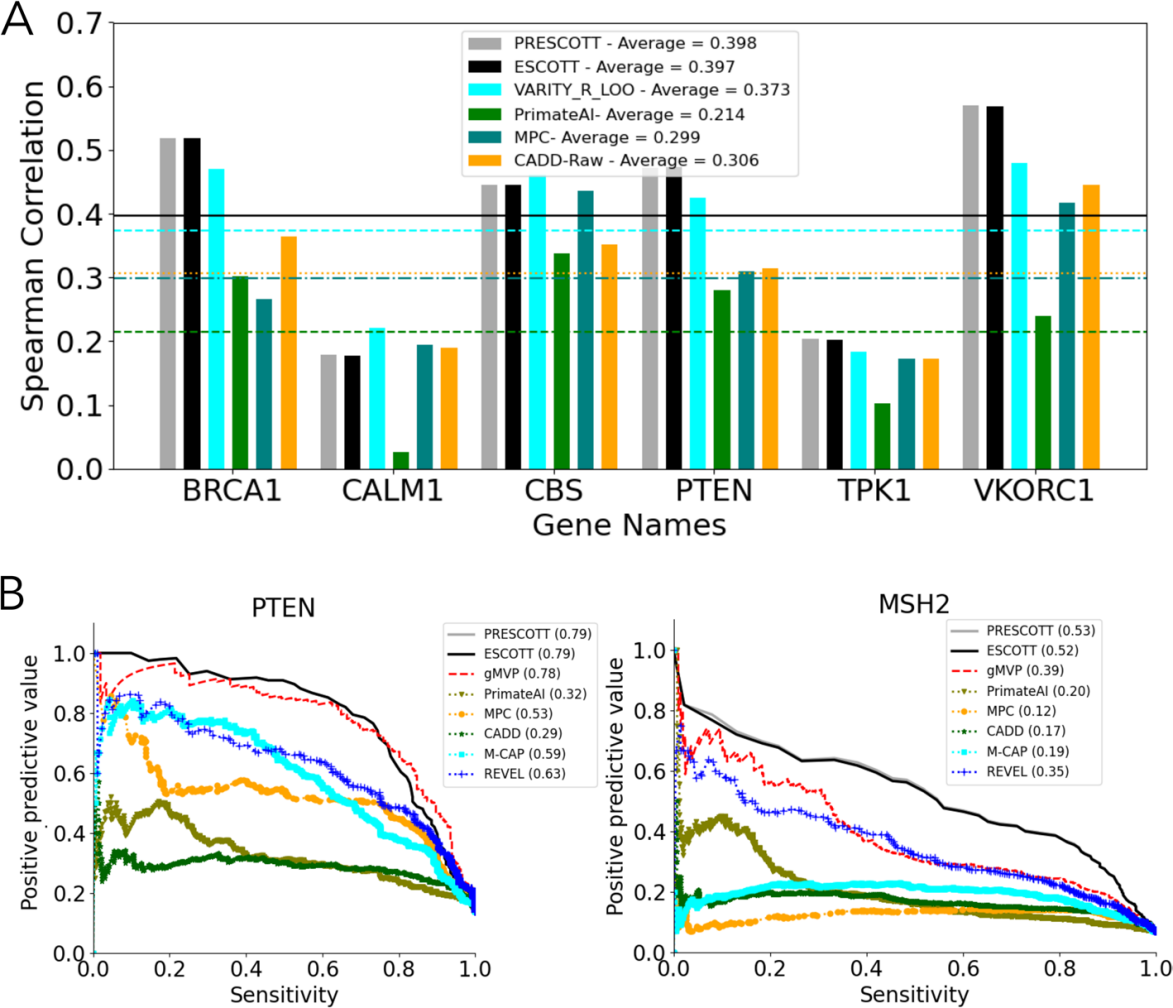
Assessing ESCOTT, PRESCOTT, VARITY, gMVP and other approaches with experimental variant effect maps. **A** Spearman correlation coefficients are computed over 10,020 experimental variant effects for six human disease related genes on VARITY, PrimateAI, MPC and CADD on a data set proposed in [9]. **B** Precision-recall curves on the two human proteins PTEN and MSH2. ESCOTT is compared to gMVP and other published methods on the two datasets of pathogenic mutations, for PTEN and MSH2, proposed in [56]

Several computational methods have been developed to specifically target rare missense variations, VARITY [9] being one of them. We compare the performance of ESCOTT against VARITY using a dataset provided in VARITY’s study. This dataset includes variant effect maps for six human genes related to diseases: BRCA1, CALM1, CBS, PTEN, TPK1, VKORC1. For this comparison, we focused on VARITY_R scores, where VARITY showed its strongest performance, and included results from other models like PrimateAI [53], MPC [54], and CADD-Raw [55]. VARITY’s superior performance among 16 reference computational methods is documented in Table S10 of their study. However, our analysis indicates that ESCOTT surpasses all these methods, VARITY included, as shown in Fig. 9A. Further, we compared ESCOTT versus gMVP [56], a method based on graph attention neural networks, over a dataset of variants for genes PTEN and MSH2 considered in [56]. Figure 9B confirms ESCOTT performance over gMVP and other predictors, REVEL [57], M-CAP [58], and MetaSVM [59], over two datasets of variants introduced in [56] (see the “Methods” section).

### The PRESCOTT online web server and its comprehensive human protein database

The PRESCOTT Online web server is expected to be a highly valuable tool in both research and clinical settings. It offers three distinct platforms: the ESCOTT web server, the PRESCOTT web server, and the ESCOTT Database. The ESCOTT Web Server allows users to perform sequence and structure-based analyses (ESCOTT) or purely sequence-based analyses (iGEMME) of proteins across all species. The PRESCOTT web server specializes in analyzing human proteins, integrating ESCOTT analysis with allele frequency data according to the PRESCOTT model. Users can access ESCOTT analysis through either the ESCOTT Web Server or the ESCOTT Database. The user can provide as input the latest gnomAD v4 dataset or custom allele frequency data. The ESCOTT Database stands out as a comprehensive repository, hosting 19,295 human proteins pre-analyzed using the ESCOTT model (see the “Methods” section). A key feature of PRESCOTT Online web server is its pathogenicity scoring system, meticulously designed to evaluate the potential of mutations to cause disease. For comprehensive analysis, users have the flexibility to download all results, including intermediate scores related to residue physico-chemical properties (PC), circular variance (CV), and evolutionary conservation, which are integral in deriving final ESCOTT and PRESCOTT scores.

#### General recommendations for using ESCOTT versus PRESCOTT

As a general rule, PRESCOTT should be used whenever allele frequency data is available, as it enhances the prediction of benign variants. However, exceptions should be considered due to the complexity of the problem. For cases where allele frequency data originates from populations with unique genetic heritage, we recommend using ESCOTT instead of PRESCOTT, as outlined above. Furthermore, our analysis of a limited set of pathogenic gain-of-function variants revealed that these are best predicted by iGEMME, which excludes structural information. Similarly, iGEMME may be preferable for revealing pathogenic mutations located in binding pockets, as these regions are typically highly conserved and thus represented by high T_JET_ scores. The score of such mutations could be affected by structural measures like CV and PC, making iGEMME better suited for their evaluation.

## Discussion

Missense variants involve single nucleotide substitutions and make up the overwhelming majority of VUS, which are of clinical interest in diagnosing disease-causing genes/mutations. The impact of these mutations on proteins is complex, affecting protein interactions, structural stability, and functional gains or losses. The manifestation of a missense variant as pathogenic can be attributed to genetic background, environment, and diet. The challenge lies in distinguishing potentially pathogenic variants from the vast majority of benign variants.

### Conceptual advances in PRESCOTT compared to GEMME

PRESCOTT models proteins by integrating information from three different biological scales: the evolutionary scale, the molecular/structural scale, and the population scale. Collectively, these factors enable PRESCOTT to optimally predict mutation effects and interpret variants. The combination of these scales in ESCOTT/PRESCOTT represents a conceptual evolution of the GEMME approach, introducing three key advancements:

- *Modeling of residue positions.* In GEMME, amino acid residues in a protein are modeled based solely on evolutionary conservation: the model details the global evolutionary conservation of a residue in the species tree and its local conservation within a phylogenetic clade. In ESCOTT/PRESCOTT, however, residues are modeled using a combination of sequence information (T_JET_) and structural information, describing the position of the residue within a structure (CV) and its physico-chemical properties (PC). This integration of evolutionary conservation with structural and physico-chemical features enables the model to account for essential factors such as whether a residue is buried in the structural core, located at a protein interface, or part of a binding site. These properties are crucial for understanding a mutation's impact on protein stability, dynamics, and function.
- *Redefinition of the epistatic effect of a mutation from its structural context.* The epistatic effect of a mutation in a query sequence q is assessed, in GEMME and other approaches, by measuring the importance of the positions that have mutated relative to q in natural sequences s carrying the mutation. The minimal effect across all natural sequences serves as a proxy. GEMME takes an evolutionary perspective, measuring the importance of a position through evolutionary conservation (T_JET_) and assessing the epistatic effect as a function of the evolutionary conservation of all residues in s that differ from q. In contrast, ESCOTT/PRESCOTT incorporates the structural context in which the mutation occurs, distinguishing between structured and coiled regions, and adjusting the modeling of positional importance accordingly. For coiled region, ESCOTT/PRESCOTT evaluates mutations similarly to GEMME, relying primarily on evolutionary conservation. However, for structured regions, ESCOTT/PRESCOTT integrates evolutionary conservation with structural and physico-chemical features to model a residue’s structural and functional importance with Maxscore. This represents a significant advancement over GEMME's purely evolutionary perspective. By incorporating the structural context, ESCOTT/PRESCOTT improves both the interpretability and predictive power of the model, enabling it to capture subtle mutational effects, such as those mediated through interactions at protein–protein interfaces. These enhancements make the model more applicable to real-world challenges and open a new perspective in modeling positional importance within a unified framework that could be further expanded to capture diverse mutational effects.
- *Incorporation of allele frequencies.* Unlike GEMME, PRESCOTT incorporates allele frequency data into its modeling. This addition enables a broader and more nuanced interpretation of mutational effects in human proteins, further enhancing the framework’s predictive and interpretive capabilities.

### The role of residue information in PRESCOTT

ESCOTT predictions of mutational effects towards hydrophilic amino acids show improved accuracy compared to hydrophobic ones (Additional file 1: Fig. S7), a trend consistent across other mutational effect predictors, including AlphaMissense, EVE, and ESM2 (not shown). This observation warrants further exploration across the five classes of DMS experiments—“Expression,” “Binding,” “Organismal Fitness,” “Activity,” and “Stability” [60]—as variations in amino acid distribution within these categories may yield deeper insights.

Hydrophilic amino acids (DEKNQ, excluding R), are generally expected to localize on the protein surface outside protein interfaces [30], whereas hydrophobic amino acids (LVYFWMC) preferentially localize at protein interfaces and within the structural core. Neutral amino acids (ATSG) are more likely to appear on the surface and less frequently at interfaces. These patterns [30] suggest that hydrophilic and neutral amino acids may have a lesser impact on a protein’s structural stability and functional activity compared to hydrophobic residues. Consequently, the challenge in predicting mutational effects likely arises from this complexity: determining whether mutated hydrophobic residues play critical structural or functional roles or have negligible effects. This intrinsic difficulty underscores the importance of incorporating residue-level context into predictive models.

Globally, ESCOTT offers a versatile framework for incorporating new models of the"importance of an amino acid"in a protein, potentially replacing the current MaxScore, which is simple and computationally efficient.

### The role of structural information in PRESCOTT

PRESCOTT underscores the importance of structural information in mutation analysis. Key mutations are often located in protein binding sites [27–29] or in the structural core [24], with more subtle long range allosteric effects also identified [61]. While the current model prioritizes simplicity for biologically justifiable improvements, integrating precise protein binding site predictions could enhance ESCOTT's ability to assess mutational effects. Similarly, incorporating domain-domain interface information into the framework presents a promising avenue for refinement, especially in light of our findings on gain-of-function mutations. Additionally, ESCOTT and PRESCOTT process unstructured protein regions by using evolutionary conservation (${T}_{JET}^{i}$) to evaluate residues in potentially disordered regions. However, more advanced modeling of important residues in intrinsically disordered regions could significantly improve predictions for these areas. These additions will be interpretable and useful for understanding the mechanical effects of mutations in proteins.

Among PRESCOTT’s four core amino acid measures—T_JET_, CV, PC, and allele frequency—T_JET_ and CV are defined at the positional level, while PC and allele frequency are defined at the residue level. Expanding the model to include mutation-dependent descriptions of T_JET_ and CV could unlock exciting possibilities, likely requiring 3D representations of residues. This enhancement would be particularly impactful in contexts where protein interfaces are explicitly incorporated into the model, enabling the classification of 3D mutational patterns and providing deeper insights into the structural and functional consequences of mutations.

### The role of allele frequency data in PRESCOTT

Population allele frequencies, a cornerstone in clinical diagnostics, are effectively utilized by both PRESCOTT and AlphaMissense. AlphaMissense is trained on allele frequencies and their known effects, whereas PRESCOTT integrates only allele frequency information to screen common variants into the final prediction stage, without relying on variant classification. PRESCOTT’s model, simple yet effective, combines ESCOTT scores based on evolution and structure with population frequencies, favoring specific human populations over a global approach [32]. In doing so, it indirectly takes into account the genetic background that extends beyond the sequence of a protein and that depends on external factors, such as the environment and diet of an individual carrying the mutation. According to our calculations, this population-specific model provides slightly better estimates of the pathogenic versus the benign status of a mutation than a global population model. Given the importance of population allele counting in the analysis, the small number of different populations that are registered today, and the small number of individuals that have been genetically screened, broader exome sequencing efforts are essential for a deeper understanding of genetic diseases and, more generally, of protein behavior. Importantly, by providing both ESCOTT and PRESCOTT scores, we offer to geneticists the choice to either consider the ESCOTT score or to rely on PRESCOTT for a fast and integrated prediction of variant pathogenicity based on gnomAD v4 or his/her allele frequency database of choice. Alternatively, based on ACMG dataset analysis reported in Fig. 8D and 8, AlphaMissense and ESCOTT could be used by a clinician as complementary assessments for a clinical decision on pathogenic variants, while PRESCOTT and AlphaMissense could be used on benign variant assessments.

As noted earlier, PRESCOTT’s approach to modeling allele frequency data can be seamlessly integrated with any predictor as a postprocessing step, unlocking the potential to improve predictions for human missense variants, as demonstrated with EVE.

### Limits of predictive models and future advances

While PRESCOTT and similar recent methods represent significant progress in mutation effect prediction, their variable performance across proteins—with Spearman correlation coefficients ranging from 0.135 for SCN5 A to 0.690 for P53—indicates that large-scale application is not yet feasible. Protein complexity arises at multiple levels: variants may differ in inheritance mode (recessive vs. dominant), structural context (disordered vs. structured regions), and experimental outcomes (binding, stability, expression, etc.). Despite these challenges, tools like PRESCOTT remain invaluable for future variant prioritization. PRESCOTT stands out for its interpretability, integrating data from evolutionary, molecular, and population scales. This multifaceted approach provides quantitative insights crucial for developing criteria to guide clinicians in making informed decisions. By combining these diverse components, PRESCOTT bridges the gap between genetic analysis and clinical practice, offering robust evidence to support healthcare applications.

The integration of advanced models for handling allele frequency data is expected to enhance PRESCOTT. Both the MPC (Missense badness, PolyPhen- 2, and Constraint) [54] and CCR (Constrained Coding Regions) [62] use population allele frequency to estimate average effect of missense variants in sub-genic regions. These models offer two distinct pathogenicity measures that help identify coding regions with a low frequency of missense variations. Such regions are known to contain a significant number of disease-related variants. These models are very interesting and differ significantly from PRESCOTT, which uses allele frequency information to exclude common variants, rather than for pathogenicity prediction like MPC and CCR. Implementing a strategy in PRESCOTT that focuses on regions with fewer pathogenic mutations, based on ESCOTT scores, could significantly enhance its capabilities. This strategy will be explored in future developments and systematically crossed with our analysis of sensitive regions. Additionally, incorporating allele frequency data from both humans and primates [63] could further improve the results.

### On loss-of-function versus gain-of-functions

The ESCOTT/PRESCOTT model does not differentiate between gain-of-function and loss-of-function mutational effects. While we explored numerous features to distinguish these two classes of mutations, we were unable to identify specific determinants. However, the observation that gain-of-function mutations are often enriched at domain-domain interfaces [21] presents a promising avenue for further investigation and may serve as a useful starting point for discriminating between these two classes of effects based on structural information.

The analysis of the MEFV gene is particularly challenging for ESCOTT/PRESCOTT and AlphaMissense. For this protein, the oligomeric state may significantly impact predictions and help explain associated challenges. Like most DMS (Deep Mutational Scanning) predictors, ESCOTT/PRESCOTT and AlphaMissense rely on single-protein analyses and do not account for protein–protein interactions. Among existing methods, only MuLAN [64] incorporates these interactions, allowing for analyses that go beyond the monomeric state. Using MuLAN—a deep learning model designed to predict changes in binding affinities within complexes—we can reconstruct MEFV's mutational landscapes with notable improvements over ESCOTT and AlphaMissense, particularly for pathogenic mutations linked to diseases. Incorporating protein interaction data into mutational landscape reconstructions could further enhance prediction accuracy and it appears as a promising direction in the field. Another critical aspect to improve MEFV predictions is its evolutionary context. The MEFV protein and its homologs likely co-evolved with virulence factors from pathogen-adapted species, leaving distinct evolutionary signals in the human sequence or a subset of homologs. Disentangling these signals requires identifying homologs, pinpointing residues under strong positive selection, and developing tailored strategies to address these co-evolutionary patterns.

## Conclusions

PRESCOTT represents a significant advancement in predicting the functional impact of missense mutations, integrating evolutionary, structural, and allele-frequency data across populations. By accurately reconstructing mutational landscapes, PRESCOTT identifies mutation-sensitive regions, allowing comparisons between landscapes associated to different isoforms, and categorizing variants confidently as pathogenic, benign, or uncertain. This method surpasses traditional approaches by leveraging comprehensive evolutionary conservation (T_JET_), structural information (described by circular variance and physico-chemical properties of residues), and allele frequency insights from diverse human populations. Crucially, PRESCOTT's ability to discern rare pathogenic mutations from benign polymorphisms is demonstrated through superior performance on ClinVar, ACMG, and ProteinGym datasets, achieving outstanding accuracy. Additionally, it effectively identifies gain-of-function and uniquely inherited pathogenic variants, underscoring its broad clinical relevance. PRESCOTT’s interpretable model, supported by intermediate structural and evolutionary scores, provides transparent, actionable insights for genomic medicine, experimental research, and drug discovery. The publicly accessible PRESCOTT Online server further broadens genomic analysis, making detailed predictions readily available for clinicians and researchers, along with access to a comprehensive database of precalculated analyses for 19,000 human proteins.

## Methods

### ProteinGym dataset

We used the ProteinGym DMS dataset (www.proteingym.org/substitutions-dms-level) to investigate the agreement between ESCOTT predictions and experimental data. The dataset contains 87 DMS experiments performed on 72 proteins. Of these 87 experiments, 76 involve single point mutations only, for a total of 266,473, and the other 11 experiments contain 1,271,579 multiple point mutations. DMS was developed to systematically quantify the effects of genetic variations on a large scale, with high efficiency and relatively low cost [65, 66]. Fitness competition, toxicity, protein stability, and binding affinity have been used to measure mutational effects for a protein-coding gene. The phenotypic values measured are then organized into large matrices of protein size per 20, i.e., the number of amino acids, reporting the measurements from each point mutation experiment.

### Multiple sequence alignments of ProteinGym dataset

We obtained MSAs from Colabfold method using uniref30_2103 and colabfold_envdb_202108 sequence databases. This method produced MSAs for all proteins in the ProteinGym dataset without any problem and we use it as reference dataset for our analyses.

### Structures generated for ProteinGym and the Human Proteins Dataset

For proteins in the ProteinGym dataset, we computed model structures with Alphafold version 2.1.2 [43], except for three of the proteins, BRCA1 HUMAN, PABP YEAST, and SRC HUMAN, whose model structures have been obtained from the AlphaFold Protein Structure Database [67]. Only the model with the highest rank was used as the reference structure. Since Alphafold implementation uses several databases such as Uniref90 [68], BFD [69, 70], and Mgnify [71], due to their large sizes, Alphafold computational time becomes prohibitively high for thousands of proteins and, for this reason, we used Colabfold, based on highly clustered databases, to generate a single model structure for proteins in the Human Proteins Dataset.

### The Human Proteins Dataset is used to define a validation set and a training set

We used 3013 clinically relevant human proteins to assess ESCOTT predictions against ClinVar labels. This dataset was used in [13] to evaluate performance of EVE method [13] against ClinVar labels (see below) and it was downloaded from evemodel.org/download/bulk. Structural models and MSAs have been generated for the entire set of 3013 proteins using Colabfold [21, 47, 48, 72, 73]. We calculated the entire single point mutational landscape for 3013 proteins (totalizing 33,771,588 missense variants) with ESCOTT using Colabfold MSAs, and conducted performance evaluation only on ClinVar mutations. Because of the comparison with EVE, ESM1b and AlphaMissense, we reduced the original number of 3,013 proteins considered by EVE to 2383. The reduction is due to several reasons: 1. no benign and pathogenic labels were both available in ClinVar, 2. no direct matching between ensemble identifiers and gene names was available, 3. no analysis was present in the AlphaMissense dataset. Note that ESM1b was run by us on all 2383 proteins.

For the purpose of calibrating default parameter values in ESCOTT, we randomly selected a subset of 500 proteins from our broader dataset of 2383 proteins, ensuring that none of these proteins are members of the ACMG list. Specifically, this subset was instrumental in setting the parameters such as “frequencyCutoff” and “scalingCoefficient” for the PRESCOTTscore-high-allele-frequencies algorithm (detailed further below), as well as establishing the two critical thresholds that distinguish benign, pathogenic, and VUS in the ESCOTT analysis. These thresholds were precisely calculated using data from 1849 pathogenic mutations and 2523 benign mutations, each linked to the aforementioned 500 proteins as classified by ClinVar. To ensure the integrity of our validation process, we excluded these 500 proteins from our final test dataset. Consequently, our validations were rigorously conducted on a separate group of 1883 human proteins. The specifics of the proteins in these two distinct datasets are available in our supplementary data files for reference.

### ACMG Actionable Genes Dataset (v3.1)

We considered the full set of 78 actionable genes from version 3.1 of the American College of Medical Genetics and Genomics (ACMG) dataset (Table 1 in [46]). The TTN_HUMAN protein was too big to be modeled and was excluded; the ACVL1_HUMAN protein was excluded because it did not belong to the EVE dataset; 12 more proteins were discharged because they had neither benign nor pathogenic labels. We remained with 64 proteins. We modeled structures of 61 of them with Colabfold [47–49, 73] and Alphafold [21]. For the remaining three (RYR1_HUMAN, RYR2_HUMAN and APOB_HUMAN) no full structural model existed.

### A new dataset of gain-of-function mutations

We focused our study on 13 genes known implicated in autoinflammatory diseases: *ALPK1*,* CDC42*,* MEFV*,* PSTPIP1*,* NLRC4*,* NLRP1*,* NOD2*,* CEBPE*,* RIPK1*,* PLCG2*,* CARD14*,* STING*, and *IFIH1*. These genes were carefully selected based on information from Infevers [74], augmented by thorough reviews of relevant literature and insights from unpublished experimental data. Our objective was to meticulously identify and select a dataset comprising over 100 missense pathogenic gain-of-function mutations, ensuring high confidence in their pathogenicity.

### The comprehensive Human Protein Database in PRESCOTT Online Web Server

The PRESCOTT Online web server features precomputed ESCOTT analyses for 19,295 human proteins, with model structures sourced from the AlphaFold Protein Structure Database (AFDB). The absence of an ESCOTT analysis for a human protein may mean that the model structure for the query is not available in AFDB, or that the Colabfold MSA may consist of only a dozen sequences, which is insufficient for calculating evolutionary conservation. In this case, the user can use the ESCOTT web server to calculate scores from her/his own model structure and MSA. It is important to note that AFDB does not include model structures for proteins exceeding 2700 amino acids in length. For analyzing these larger proteins, users are advised to employ Colabfold to create MSAs and generate structures of lower accuracy for ESCOTT web server analysis. In this regard, note that eight human proteins—APC, FBN1, BRCA2, FLNC, DESP, RYR1, RYR2, APOB—listed in the ACMG dataset are excluded from the ESCOTT database due to their length. Their analyses are instead provided in the dedicated ACMG folder.

### The gnomAD database

The Genome Aggregation Database (gnomAD) is currently the largest and most widely used publicly available collection of population variation from harmonized sequencing data (https://gnomad.broadinstitute.org/news/2023-11-gnomad-v4-0/) enabling variant analysis [32]. The gnomAD dataset is used in the majority of rare disease analysis pipelines in both diagnostic and research settings around the world. gnomAD follows the rigorous guidance for the evaluation and aggregation of variant evidence determined by ACMG and Association for Molecular Pathology (AMP) [75]. There are three available versions, gnomAD v2.1, which contains data from 125,748 exomes and 15,708 whole genomes, all mapped to the GRCh37/hg19 reference sequence, gnomAD v3.1, which contains 76,156 whole genomes (and no exomes), all mapped to the GRCh38 reference sequence, and gnomAD v4.0, which is nearly five times larger than the combined v2/v3 releases and consists of exome sequencing data from 730,947 individuals, and genome sequencing data from 76,215 individuals. Both callsets were aligned to build GRCh38 of the human reference genome. The results reported in this study refer to gnomAD v4, which includes a higher number of coding variants compared to the previous versions. It covers data from 8 populations: gnomAD collects data from eight different populations, African/African American, Admixed American, Ashkenazi Jewish, East Asian, European (Finnish), European (non-Finnish), South Asian, and Middle Eastern. As for the number of individuals in gnomAD v4.0, only one population has less than 10,000 individuals (Middle Eastern = 3031 individuals). In addition, there is only one population with less than 20,000 individuals (Ashkenazi Jewish = 14,804 individuals). The remaining populations have more than 20,000 individuals. gnomAD uses the ClinVar classification of variants into five categories: benign, likely benign, uncertain significance, likely pathogenic, and pathogenic. PRESCOTT has been run on the 1883 distinct proteins in gnomAD, comprising 7954 pathogenic mutations and 9276 benign ones (Table 2). These mutations are associated to their population origin. To compare PRESCOTT performance to EVE performance, we considered ClinVar labels from gnomAD and searched for the corresponding protein in the EVE data. If EVE computed mutational values for that mutation, we considered it.

**Table 2:** gnomAD v 4.0 (November 2023) database characteristics

Stars	Distinct proteins	Pathogenic	Benign
≥ 0	1883	7954	9276
≥ 1	1761	5351	9112
≥ 2	1133	2509	4039
≥ 3	22	335	365

### The ClinVar dataset and its stars subsets

The ClinVar database includes germline and somatic variants of any size, type or genomic location. Interpretations are submitted by clinical testing laboratories, research laboratories, locus-specific databases, expert panels and practice guidelines [76, 77]. gnomAD data are annotated by ClinVar labels as VUS, pathogenic or benign. Precisely, ClinVar labels “Pathogenic,” “Pathogenic/Likely pathogenic,” and “Likely pathogenic” were employed to define the set of pathogenic mutations in PRESCOTT. Similarly, we selected “Benign,” “Benign/Likely benign,” and “Likely benign” labels to form the dataset of benign mutations. Also, the mutations reported in ClinVar contain information about review status of each mutation (www.ncbi.nlm.nih.gov/clinvar/docs/review_status/), expressed with an integer number of “gold stars” indicating the number of sources that verified the effect of the mutation: 0 stars indicate mutations submitted without interpretation; 1 star indicates mutations provided by one submitter with interpretation and evidence; 2 stars indicate mutations submitted by two or more labs with the same interpretation; 3 stars indicate a review from external panels. The full ClinVar dataset has been referred to as “star 0,” indicating the fact that many alleles have been reported without interpretation. Three smaller datasets, “star 1,” “star 2,” and “star 3” are defined inclusively, e.g., “star 2” includes all mutations labeled with 2 and 3 stars. The mutations in gnomAD labeled with ClinVar annotations used in our analysis are described in Table 2.

Note that the amino acid mutations reported in ClinVar stand one nucleotide position away from the amino acid reported in the reference human protein. This means that instead of the full ESCOTT predicted matrix, PRESCOTT evaluates only some of the positions of the matrix, namely those that are supposed to appear in human populations, possibly causing diseases. The November 2021 ClinVar report on these mutations is used in our analysis to align with the version employed by EVE, ensuring a consistent basis for comparison.

### Datasets of variants used for comparing ESCOTT and PRESCOTT with VARITY, gMVP and other predictors

Comparison with VARITY has been performed on a dataset including variant effect maps for six human genes related to diseases, BRCA1, CALM1, CBS, PTEN, TPK1, and VKORC1, introduced in [9]. Comparison with gMVP [56] has been performed on the dataset of variants for genes PTEN and MSH2 considered in [56], which exclusively comprises single nucleotide variants derived from DMS experiments [78, 79], 1859 variants for PTEN and 5853 for MSH2.

### Details on the ESCOTT model

#### The ESCOTT model incorporates secondary structure information and knowledge of the protein core

We used Biotite Python package [80] and DSSP [81] to assign secondary structure of each amino acid in the protein structures. In ESCOTT, after the secondary structure assignments, we selected MaxScore when an amino acid is in any one of these secondary structural elements: H, E, G, B, I, S, and T. When the amino acid is in a coiled region, we performed a conditional selection. We selected MaxScore if the coil is shorter than 5 amino acids and we selected ${T}_{JET}$ score for all the other coils. This approach helped us to distinguish coils linking secondary structural elements such as helices or strands, from coils in disordered regions. Even if slightly different coil lengths are selected [4–6], our method produces similar results (not shown).

To compute whether a residue belongs or not to the protein core, we use its circular variance (CV), a measure of the vectorial distribution of a set of neighboring points around a fixed point in 3D space [26, 82]. For a given residue, CV reflects the density of the protein around it. The CV value of an atom *i* is computed as:

$$CV\left(i\right)=1-\frac{1}{{n}_{i}}\left|\sum_{j\ne i,{r}_{i}\le {r}_{c}}\frac{\overrightarrow{{r}_{ij}}}{\Vert {r}_{ij}\Vert }\right|$$

where *n*_*i*_ is the number of atoms distant by less than r_c_ Å from atom *i*. The CV value of a residue *j* is then computed as the average of the atomic CVs, over all the atoms of *j*. A low CV value indicates for a residue that it is located in a protruding region of the protein surface. CV values are scaled between 0 (most protruding residue of the protein) and 1 (least protruding residue of the protein) for the calculation of residue scores. The value r_c_ is set to 7 Å after an evaluation on best performance over the set of DMS experiments (Additional file 1: Fig. S12). This default value has been used in all validation testing reported and on the predictions of the 19,000 proteins.

An evaluation of the predictions on structured versus coiled regions along the sequence is reported in Additional file 1: Fig. S5 for all proteins in the ProteinGym dataset. As expected, the analysis shows that predictions are more precise on structured regions.

#### The ESCOTT model incorporates physico-chemical propensities of amino acids at the interface

Physico-chemical properties (PC) of amino acids are derived from propensities specific to every amino acid to be located at a protein interface, taken from [30]. The original values, ranging from 0 to 2.21, are scaled between 0 and 1 for the calculation of residue scores.

#### ESCOTT performance and impact of the alignments in ProteinGym

Changes in the underlying sequence datasets used as input for the proteins can induce slight performance variations. We evaluated two datasets: Uniref100 (with EVcouplings sequence selection approach [10]) and the highly clustered Colabfold [21, 47, 48, 72, 73], obtaining average Spearman correlations of 0.491 and 0.492, respectively, across 87 DMS experiments. Nevertheless, the primary reason for selecting Colabfold was its superior speed.

#### The ESCOTT program is built on iGEMME

ESCOTT relies on an improved version iGEMME of GEMME. In iGEMME, the characteristics of the input sequence dataset and default parameters are optimized in four ways compared to the original GEMME program:

1. When a user does not provide an MSA, the default method to obtain MSAs in the GEMME webserver is PSI-BLAST [83, 84]. In ESCOTT, we use the Colabfold method [73] due to its efficiency and clustering procedure.
2. The maximum number of sequences processed has been increased from 20,000 to 40,000.
3. A parameter that restricts the number of sequences due to computational load (CPU load or max_load parameter) was increased from 500,000 to 8,000,000.
4. The maximum heap size in the calls of the JET program was increased from 1024 to 8192 MB.

These parameters’ changes allow us to handle large proteins and MSA files containing thousands of sequences in ESCOTT. Additional file 1: Fig. S13 provides a comparison of ESCOTT with iGEMME and GEMME. The iGEMME average Spearman over the entire ProteinGym dataset of 0.472 (red) improves GEMME performance (0.464; violet).

### Details on the PRESCOTT model

#### PRESCOTT model and population frequency in gnomAD

For the analysis of human mutations, PRESCOTT considers the 8 populations in gnomAD independently and establishes, for each population, the PRESCOTT score by integrating to the ESCOTT score a term which is dependent on a popmax gnomAD population frequency. This is done with the following algorithm depending on two parameters, the “frequencyCutoff” and the “scalingCoefficient”:

**Figure:**
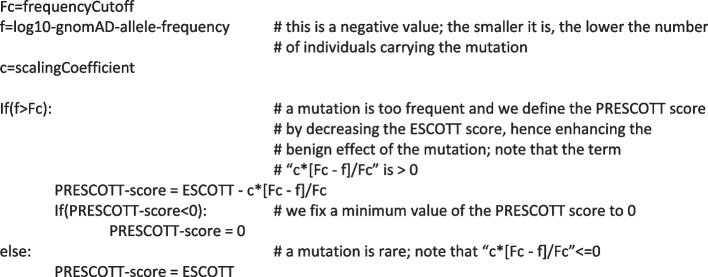
**Algorithm 1.** PRESCOTTscore-high-allele-frequencies

with default parameter values frequencyCutoff = − 4 (where − 4 = log_10_(0.0001)) and scalingCoefficient = 1. They have been determined based on best performance over the eight populations of the gnomAD v4.0.0 dataset (see Additional file 1: Fig. S14 A) and the training set of 500 proteins defined from the Human Protein dataset.

All tests reported in the article have been realized with these default values. After evaluating a mutation on each population, PRESCOTT chooses the popmax allele frequency, defined as the population maximum allele frequency in the continental populations (African/African American, Admixed American, Ashkenazi Jewish, East Asian, European/Finnish, European/non-Finnish, South Asian, and Middle Eastern) as recommended in [32]. Generally, if a variant is common in one population of healthy individuals, it can be assumed to be benign across all populations. Based on this idea, the algorithm enhances a PRESCOTT value towards 0 by calibrating it towards a “benign” prediction. It is important to note that this principle is idealized, as gnomAD heavily depletes data from individuals with severe early-onset diseases. However, it only mildly depletes or even includes data from individuals with phenotypes such as infertility, vision and hearing impairment, or conditions with late onset or reduced penetrance [32]. As a result, a clinician's understanding of potential biases—such as the Finnish case discussed in the manuscript or similar situations—can guide the decision to favor ESCOTT over PRESCOTT when appropriate.

It is also important to note that the Middle Eastern population, currently consisting of 3031 individuals, is relatively small compared to the cutoff threshold of − 4 (equivalent to 10,000 individuals) used in the PRESCOTT model. However, since the parameters (fc and c) derived from our training set of 500 proteins and testing set of 1883 proteins remain consistent even when excluding this population, we have opted to include it in the model. This decision ensures that, in the near future, users of the PRESCOTT software will benefit from these statistics, which are currently underrepresented. We anticipate that the availability of allele frequency data from Middle Eastern populations in gnomAD will increase over time, further enriching the model. Note that the PRESCOTT software informs the user of the population origin used in the calculations, facilitating the evaluation of the scores.

A symmetric treatment of high scores could enhance the prediction of pathogenic mutations. For this, we defined the following algorithm:

**Figure:**
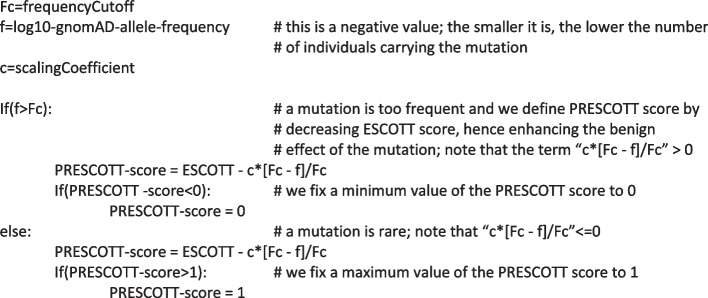
**Algorithm 2.** PRESCOTTscore-all-allele-frequencies

with default parameter values frequencyCutoff = − 3.5 and scalingCoefficient = 0.75, determined on a best performance on the gnomAD database (Additional file 1: Fig. S14B) and the training set of 500 proteins defined from the Human Protein dataset. The idea of this algorithm is that a mutation that is frequent in some population is most probably “benign” and, for this, it reduces the score, as in PRESCOTTscore-high-allele-frequencies. In contrast, if a mutation is rare in all populations, it enhances ESCOTT value towards a “pathogenic” prediction by augmenting the PRESCOTT value towards 1. Note that the lack of information in gnomAD due to a relatively small number of populations and individuals sequenced, introduces biases towards pathogenic diagnoses which will improve in the future, with higher population sampling. In Additional file 1: Fig. S14B, one can clearly see a small number of benign mutations that end-up with high PRESCOTT scores. This effect could be undesirable for clinicians, but interesting for suggesting candidates for experimentation. We believe that sufficient data are not yet present in public databases to safely produce predictions based on this hypothesis. The algorithm is implemented in the PRESCOTT package but it not used as a default method.

The frequency of gnomAD alleles in the Star > 0 set, considering all populations at once, was tested, but performance was slightly inferior, with an AUC of 0.94 obtained on the testing set of 1883 human proteins in gnomAD (compare to Fig. 7A).

### Comparison with EVE, ESM1b, and AlphaMissense

EVE results on the 87 ProteinGym experiments and on the 3013 human proteins were downloaded from www.proteingym.org/substitutions-dms-level and evemodel.org/download/bulk, respectively. Note that the label “EVE (ensemble)” for the ProteinGym dataset indicate that EVE scores were calculated from the approximate EVE posterior distribution by ensembling scores over 5 independently trained variational autoencoders (see Extended Data Fig. 2 in [13]). EVE (ensemble) was used for all 87, single and multiple, mutation experiments. EVE (ensemble) predictions cover the full set of mutations in DMS matrices. For the ~ 3000 proteins, EVE(ASM) results were used. They possibly contain missing regions. On the ProteinGym experiments, performance of all other tools cited here are taken from www.proteingym.org/substitutions-dms-level. ESM1b was downloaded from github.com/ntranoslab/esm-variants and run on all single point mutation predictions for 3013 proteins (on a Dell Precision 7920 Workstation with 40 CPUs and 64 GB RAM).

### Computational time performance of ESCOTT on the ProteinGym dataset

The implementation of the ESCOTT model is computationally very efficient. All proteins in the ProteinGym dataset of about 1.5 million mutations can be completed within 6.16 h (Additional file 1: Fig. S15). The most time consuming experiments are the highly dense HIS7_YEAST_Pokusaeva_2019, which contains 496,137 multiple point mutations [85] processed with a total computing time of almost an hour. HIS7_YEAST is 220 amino acid long and the MSA file used for the predictions contains a large number of sequences [20]: EVE computing time is a few orders of magnitude higher than iGEMME and ESCOTT methods.

### ESCOTT ranksorted scores

A direct comparison with EVE on the Human Protein dataset and ClinVar dataset asks for a normalization of ESCOTT scores in the range [0,1]. ESCOTT scores in the entire ESCOTT matrices have been normalized with ranksorting, using the command line “from scipy.stats import rankdata”. After the data is ranked, score positions in the ranked list are divided by the number of datapoints in the list to make values ranging in [0,1]. Due to the direct comparison with EVE scoring, we reversed the ranksorted scores so that values close to 1 will be intended as “pathogenic” and those close to 0 as “benign.” Note that ESCOTT (and iGEMME) low raw scores, approximately varying from − 10 to − 4, correspond to pathogenic mutations. The use of ranksorting is indicated in all corresponding analysis.

It's important to mention that ranksorting is applied to individual genes or proteins. In fact, the ESCOTT model employs evolutionary measures that differ across proteins, identifying some as less conserved and others as highly conserved. This leads to an internal re-evaluation of the score values assigned to each residue within a protein. Consequently, such reassessment amplifies the variations in score values within the protein itself.

PRESCOTT scores are computed from the ranksorted ESCOTT matrix. If provided with the matrix of raw ESCOTT scores, PRESCOTT first ranksorts them before adjusting the values based on allele frequencies.

### Classification of human variants as benign, pathogenic, and VUS by iGEMME and ESCOTT using PRESCOTT thresholds

The automated classification of human variants by iGEMME and ESCOTT relies on the same thresholds established through PRESCOTT analysis: variants with score ≤ 0.28 are categorized as benign, those ≥ 0.42 as pathogenic, and scores within this range are designed as VUS.

### Software

PRESCOTT source code is publicly available with open-source GPLv3 License. iGEMME and ESCOTT are a part of the PRESCOTT software. The user should provide an alignment file (mandatory argument) and a pdb file (optional argument) as input. If a pdb file is not provided, ESCOTT model evaluates the role of each residue in a sequence by considering its evolutionary conservation (T_JET_) as the default feature for the amino acid. Otherwise, it uses the two-component max value terms, respectively combining circular variance, CV, and physico-chemical properties, PC, with evolutionary conservation. As a result, complete matrices describing mutational effects are provided, even in absence of a structural model. In this case, they will correspond to iGEMME. A PRESCOTT docker image is available. It includes all necessary dependencies. We recommend using the Docker image for scenarios involving very long reference sequences or batch processing of multiple reference sequences. The user should make sure that, for each reference sequence, the reference sequence contains no gaps and that the corresponding MSA comprises a sufficient number of sequences.

### PRESCOTT Online Web Server

PRESCOTT Online Web Server is accessible at the link http://prescott.lcqb.upmc.fr. The user can interact with the graphical interface as explained in the online help. To illustrate the output files of PRESCOTT Online, consider gene name ALPK1. The ESCOTT output files are organized as follows:

1. Raw escott predictions (output file): ALPK1_normPred_evolCombi.txt.
2. Ranksorted (between 0 and 1) ESCOTT predictions in csv format (output file):
  ALPK1_normPred_evolCombiTransposedRanksorted.csv
3. T_JET_ (column named “trace”), PC and CV scores for each amino acid (output file from the run of the JET2 program): ALPK1_jet.res.

PRESCOTT uses ESCOTT raw data file (ALPK1_normPred_evolCombi.txt) and gnomAD frequency file downloaded from gnomAD v4.0.0 web page (gnomAD_v4.0.0_ENSG00000073331_ALPK1.csv) as input. Any other text file (.txt) of allele frequency data following the format explained in the help of the server is also accepted. Note that the ESCOTT raw scores file is automatically ranksorted before processing. However, the user can provide ranksorted ESCOTT scores directly and the automatic ranksorting step will be skipped. PRESCOTT provides two output files with prefix name “ALPK1_prescott”:

1. A table in csv format containing all scores for single point mutations: ALPK1_prescott.csv.
2. This file does not provide a direct access to the differences with ESCOTT scores.
3. A column csv file (column names: mutant, ESCOTT, protein, log10frequency, labels, position, Selected population, PRESCOTT) that gives precise information about all positions. Note that ESCOTT = ESCOTT score, PRESCOTT = PRESCOTT score, protein = protein name, labels = ClinVar label (if the Clinvar label exists in gnomad csv file, it is added to this column, else the column remains empty), log10frequency = log10(allele frequency), position = position in the sequence. File name is ALPK1_prescott-details.csv.

## Supplementary Information

Additional file 1: All supplementary figures.

Additional file 2: Table S1—Mann–Whitney paired u-tests for data in Fig. 3A, Fig. 5A and Fig. 7C.

Additional file 3: Data from the analysis of isoform- 1 and isoform- 8 of TP53 human gene.

Additional file 4: Table S2—Analysis of 22 missense mutations associated with the Finnish Disease Heritage.

Additional file 5: Table S3—Analysis of the STAT1 gene with ESCOTT, PRESCOTT, iGEMME and AlphaMissense.

Additional file 6: Table S4-Analysis of the PTPN11 gene with ESCOTT, PRESCOTT, iGEMME and AlphaMissense.

Additional file 7: Data from the analysis on gain-of-function mutations.

Additional file 8: Review history.

## Data Availability

Public data used in this work: ProteinGym DMS [86], ClinVar data [87], EVE DMS predictions [88], GnomAD v4 data [89], AlphaFold model structures of human proteins [90], AlphaMissense pathogenicity scores of missense variants [91]. Data generated in this work is provided in Zenodo repositories with licence CC-BY-SA-NC 4.0 International [92–95]. Software is made available with this work in Zenodo (https://zenodo.org/records/10442848) and as a Docker image (https://hub.docker.com/repository/docker/tekpinar/PRESCOTT-docker) under the license GPLv3 [96, 97].
