# Supplementary material for: PRESCOTT: a population aware, epistatic, and structural model accurately predicts missense effects
Source: Genome Biol. 2025 May 6;26:113. doi: 10.1186/s13059-025-03581-y (PMC12054230; doi:10.1186/s13059-025-03581-y)
Supplement: Supplementary file 1 — Additional file 1: All supplementary figures. [file 13059_2025_3581_MOESM1_ESM.pdf]

SUPPLEMENTARY MATERIALS

PRESCOTT: a population aware, epistatic and structural model accurately predicts missense effects

M.Tekpinar, L.David, T.Henry, A.Carbone

Supplementary Figures

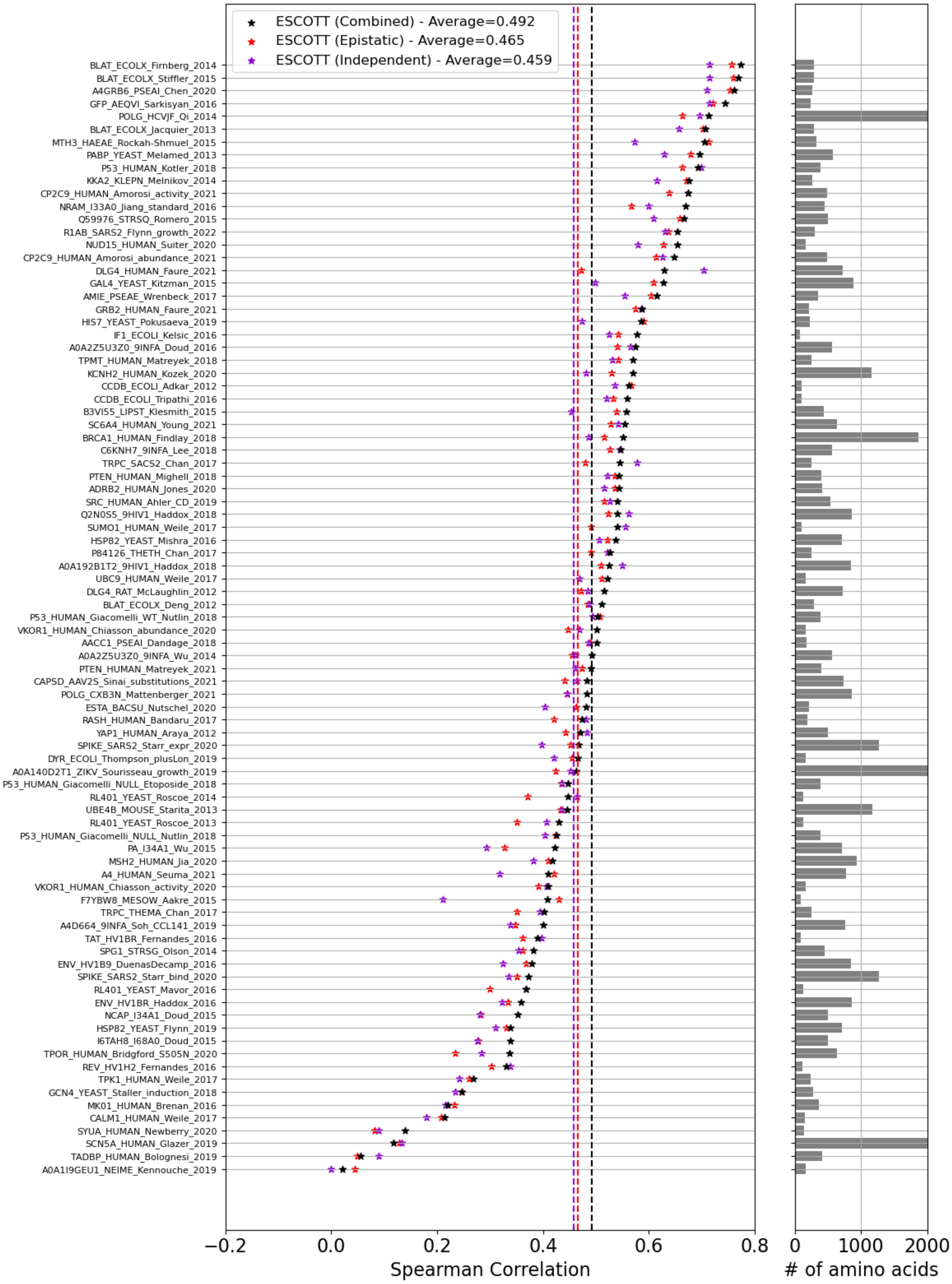

Fig S1. Analysis of the contribution of the independent and epistatic terms in ESCOTT. Stars indicate the Spearman correlation coefficient between predictions obtained with ESCOTT (black), the ESCOTT independent term only (red) and the ESCOTT epistatic term only (purple) on the 87 experimental data of the ProteinGym dataset. Dashed lines report averages of the three models over the full dataset. Horizontal bars on the right show the number of amino acids in each protein.

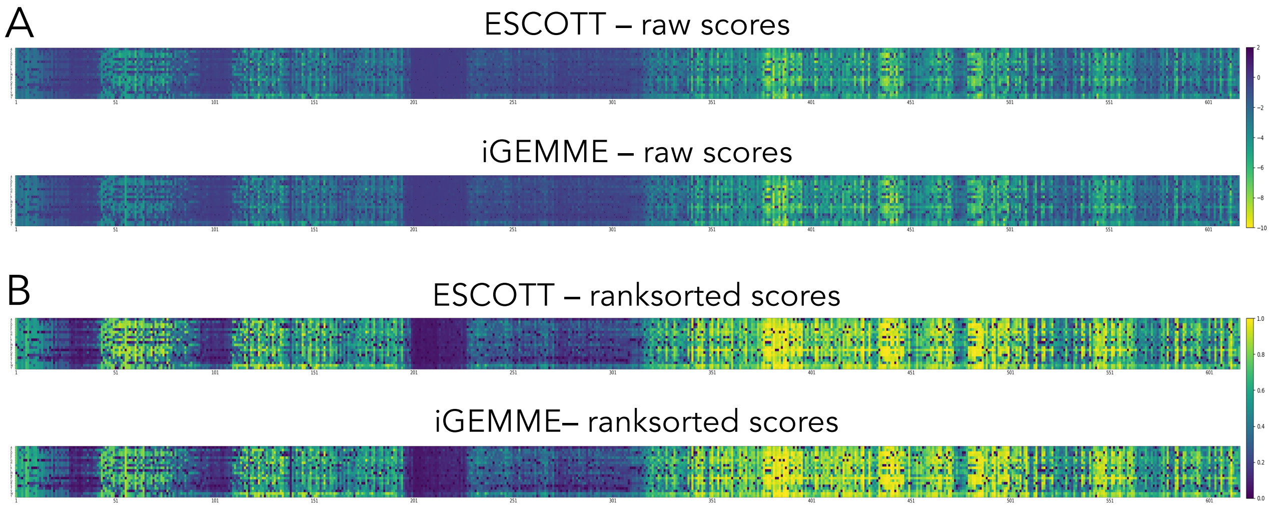

Fig S2. ESCOTT and iGEMME mutational matrices of the spastin protein. Comparison between raw (A) versus ranksorted (B) scores for the spastin protein full length predictions. Raw ESCOTT and iGEMME scores vary from -10 to 2 and ranksorted scores vary from 0 to 1. iGEMME and ESCOTT highlight essentially the same sensitive (lighter) regions of the protein. Ranksorted scores better emphasize these regions, with more contrasted score values for ESCOTT compared to iGEMME.

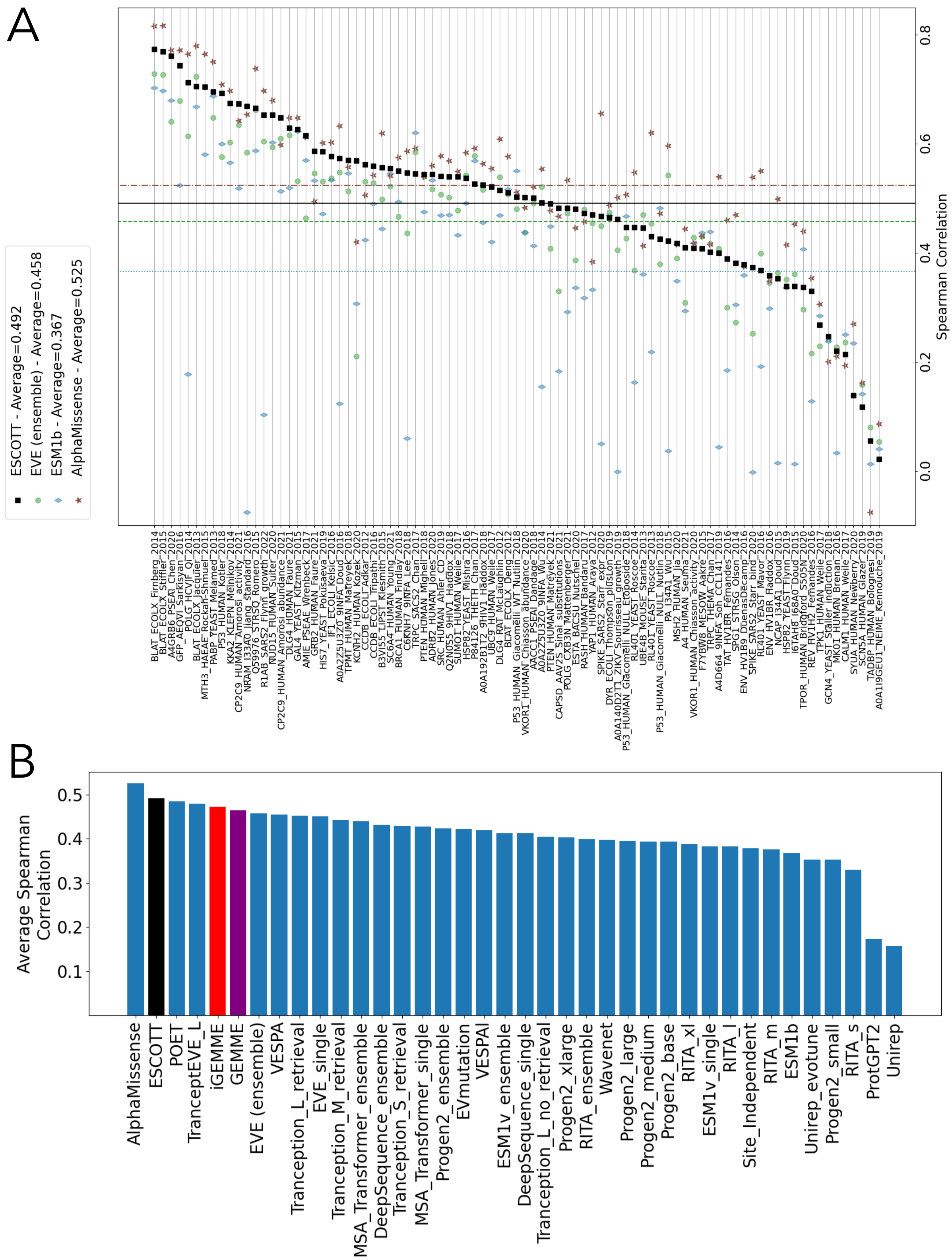

Fig S3. Comparison of ESCOTT, AlphaMissense, EVE (ensemble), ESM1b, and other methods on 87 deep mutational scanning experiments. A. ESCOTT (black squares), EVE (ensemble, green circle), ESMb1 (blue diamond), and AlphaMissense (brown stars) Spearman correlation coefficients of the predictions with experimental data from the 87 Deep Mutational Scanning experiments of the ProteinGym dataset. Lines indicate averages: solid black line for ESCOTT, dashed dotted brown line for AlphaMissense, dashed green line for EVE (ensemble), dotted blue line for ESM1b. **B.** Comparison of ESCOTT with existing methods. The list of methods includes EVE (ensemble), AlphaMissense, GEMME, and ESM1b. ESCOTT is black, iGEMME is red and GEMME is purple. Data for all other methods was obtained from https://www.proteingym.org/substitutions-dms-level, except for AlphaMissense and PoET which are reported from (18) and (77) respectively. TranscriptEVE_L code was not available at the time of writing.

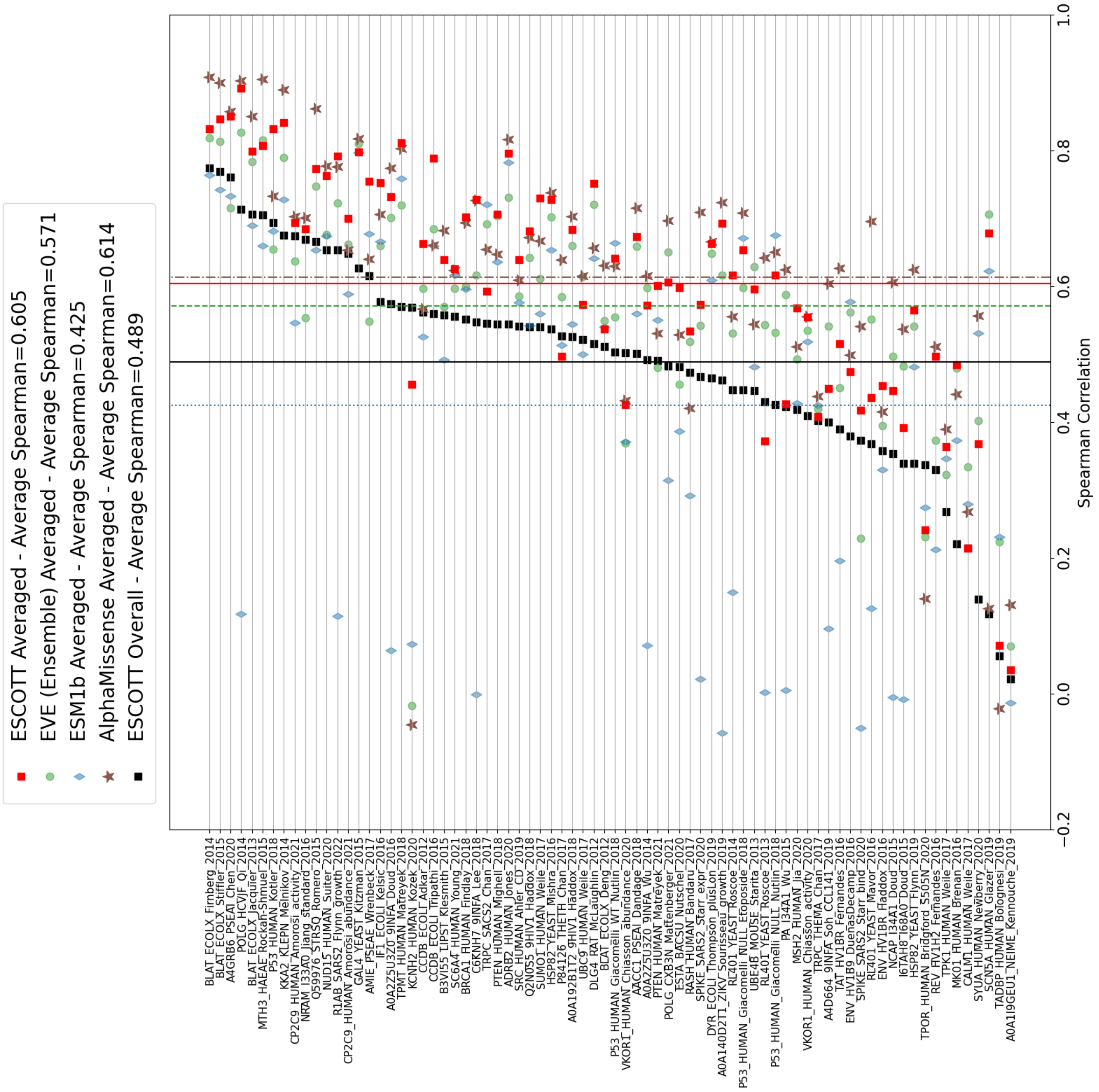

Fig S4. Spearman correlation coefficients for proteins in the ProteinGym dataset based on positional average scores: comparison of ESCOTT, EVE, ESM1b and AlphaMissense. Spearman correlation coefficients computed for the ESCOTT score matrices and the corresponding DMS matrices (black) versus Spearman correlation coefficients computed for the corresponding positional average vectors (red). Only proteins of the ProteinGym dataset with single mutation experiments are considered. Black squares correspond to the values shown in **Additional file 1: Fig. S3** for ESCOTT. Performance of EVE (ensemble, green circle), ESMb1 (blue diamond), and AlphaMissense (brown stars) is also shown.

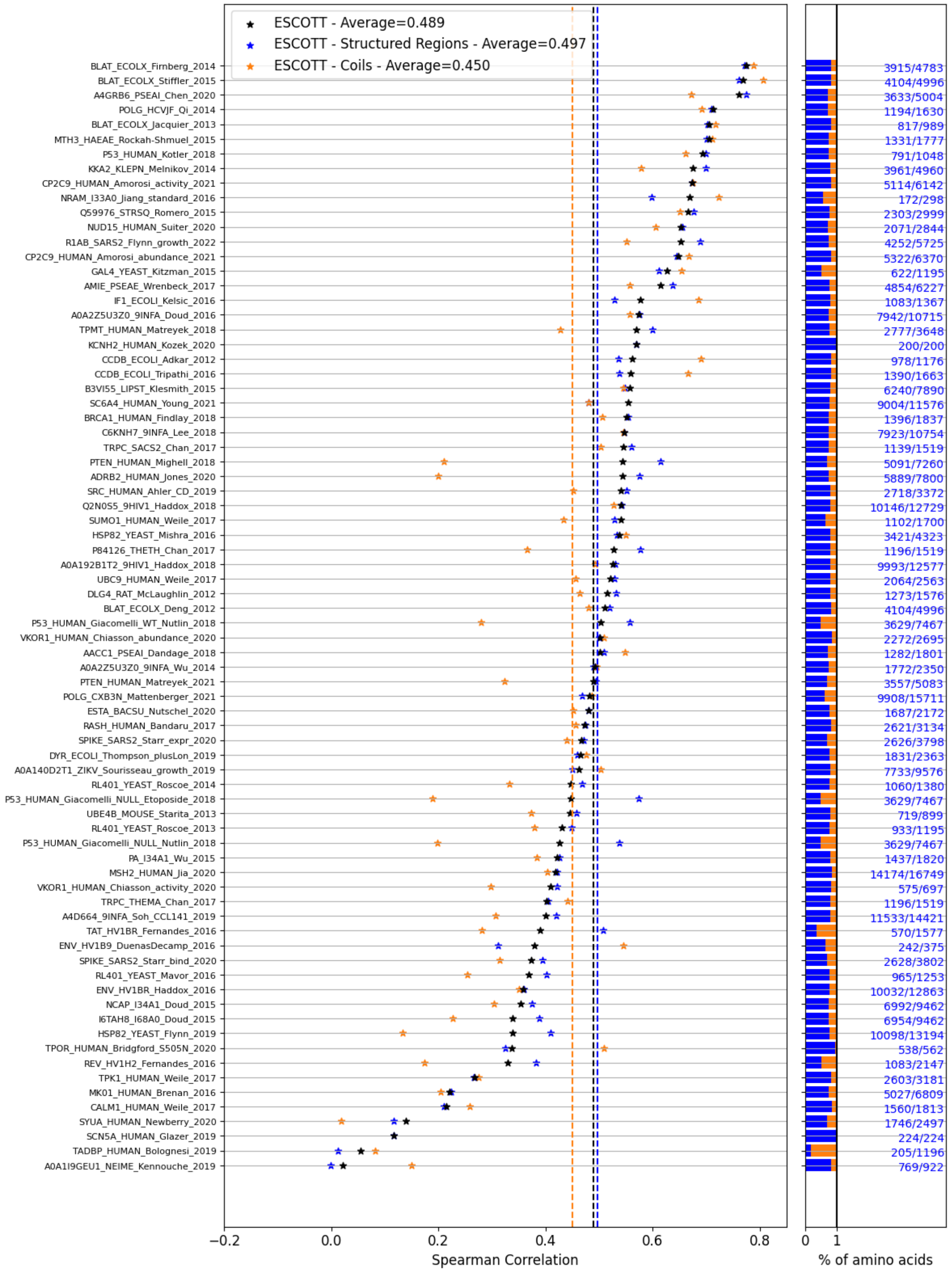

Fig S5. Comparison of ESCOTT and AlphaMissense for all mutations (black) versus mutations located in structured (blue) and coiled (orange) regions. Experimental measures and **PRESCOTT** scores are considered for the 76 **single point mutation experiments** in ProteinGym. Structured regions include H, E, G, B, I, S and T structural types as described in DSSP notation. Horizontal bars on the right show the percentage of mutations located in structured (blue) and coiled (orange) regions, for each protein. Note that some positions in a protein might have been discarded from counting because no experiment was reported on it. Dashed lines indicate averages.

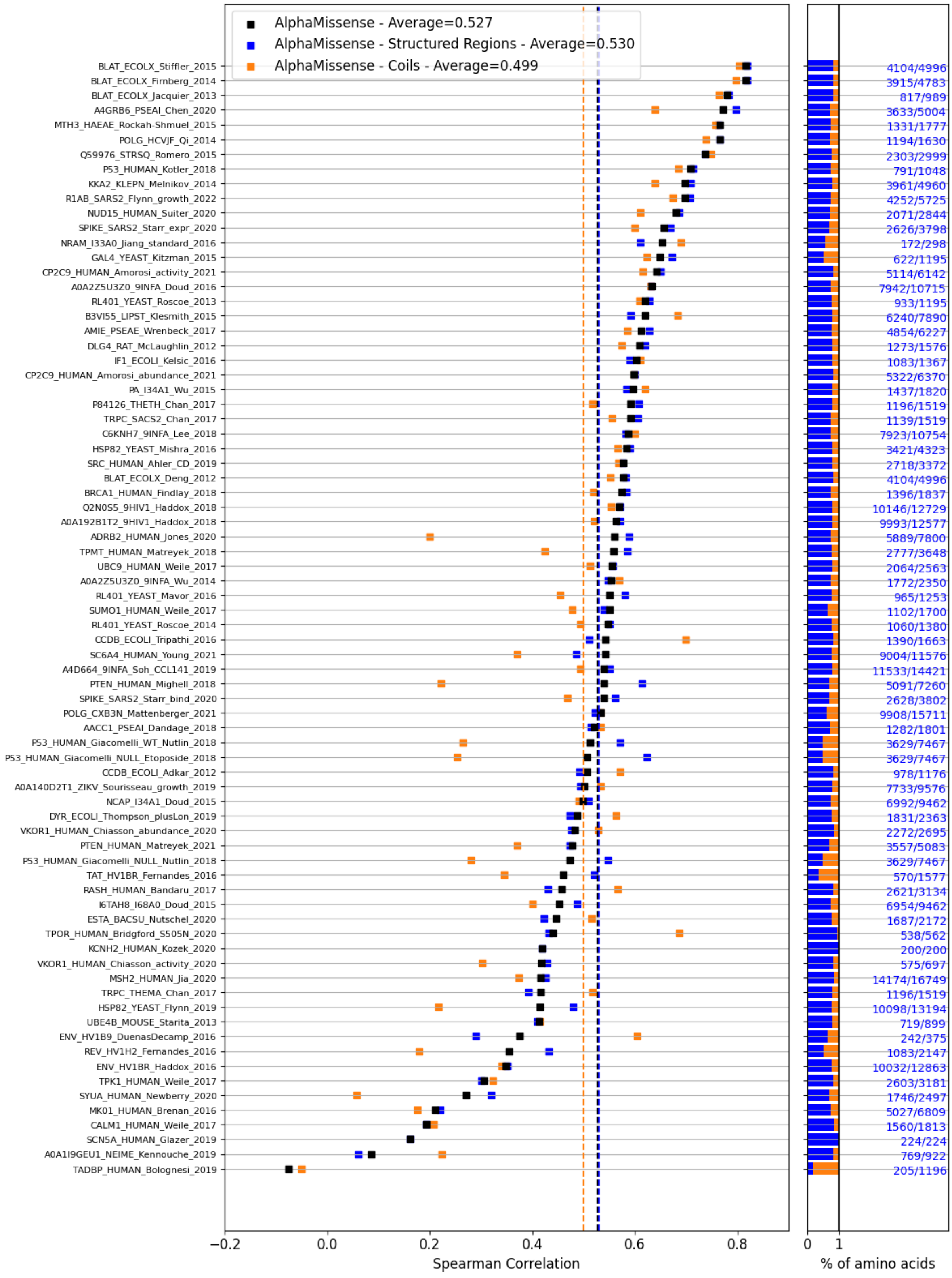

Fig S5 (continued). Experimental measures and **AlphaMissense** scores are considered for the 76 **single point mutation experiments** in ProteinGym.

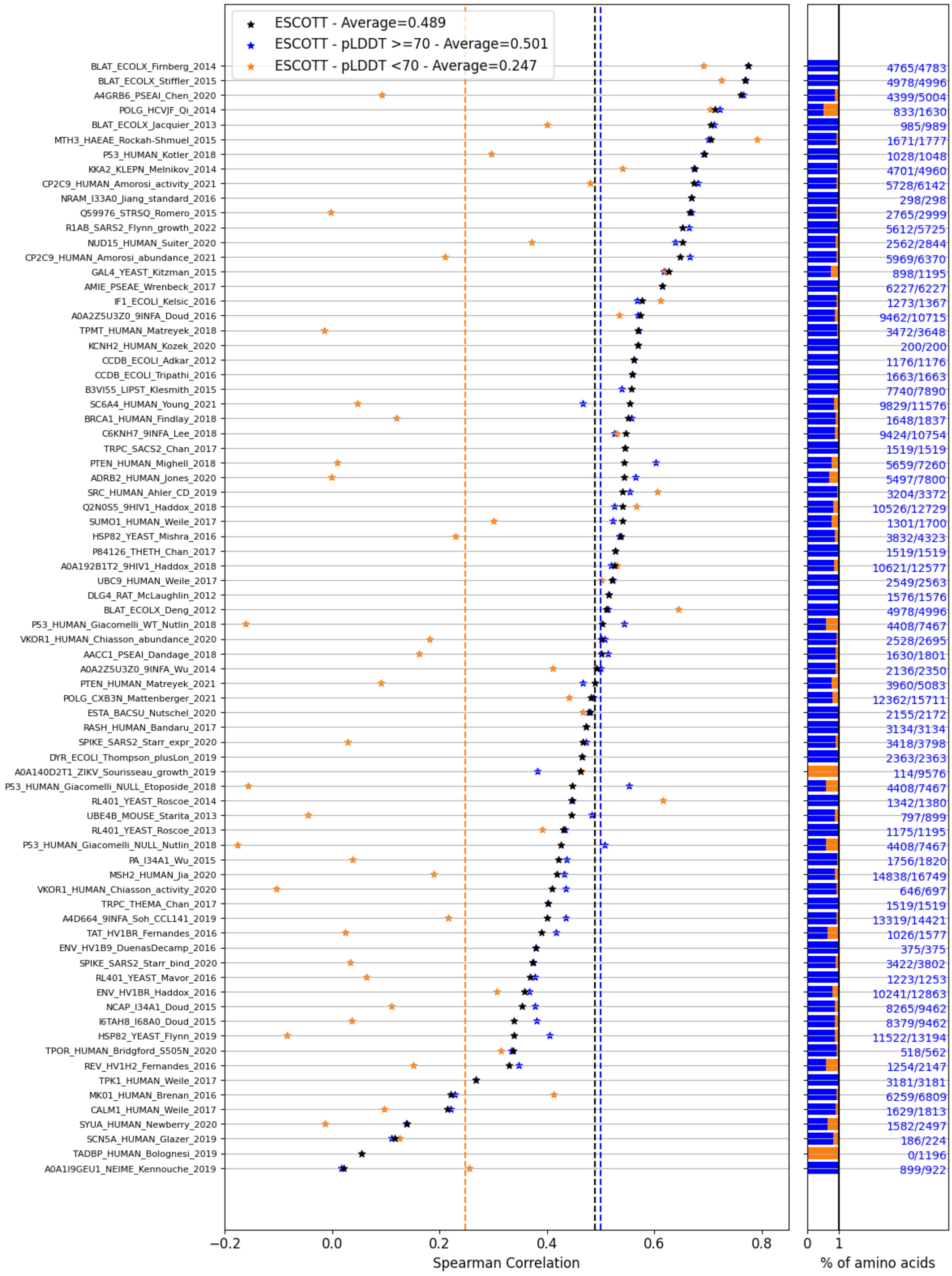

Fig S6. Comparison of ESCOTT and AlphaMissense for all mutations (black) versus mutations located in high confidence (blue) and low confidence (orange) regions. Experimental measures and **PRESCOTT** scores are considered for the **76 single point mutation experiments** in ProteinGym. High confidence regions refer to those regions that AlphaFold predicts with a pLDDT score >.7 and low confidence mutations are those predicted with a pLDDT score <.7. Horizontal bars on the right show the percentage of mutations located in high confidence (blue) and low confidence (orange) regions, for each protein. Note that some positions in a protein might have been discarded from counting because no experiment was reported on it. Dashed lines indicate averages.

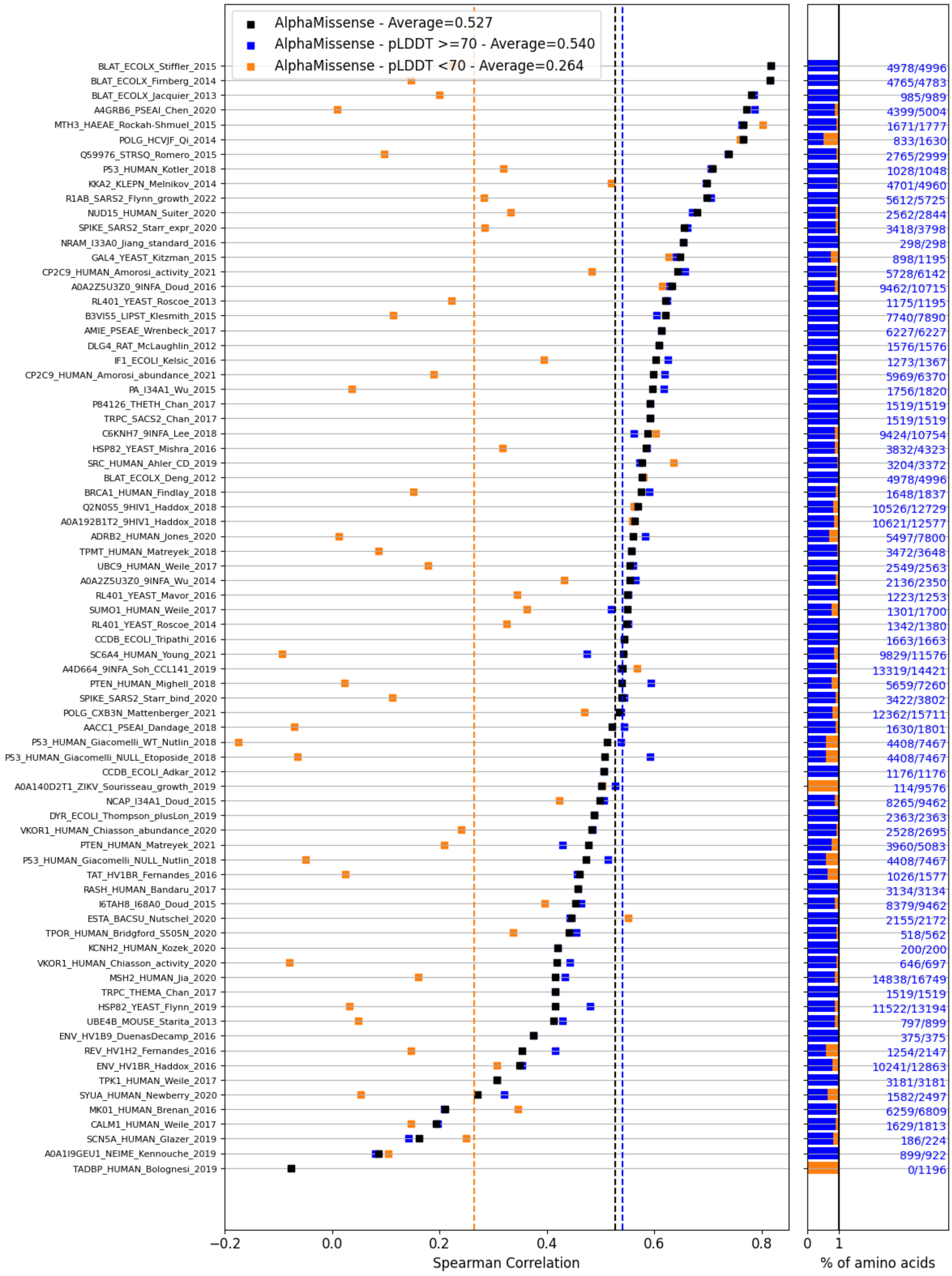

Fig S6 (continued). Experimental measures and **AlphaMissense** scores are considered for the **76 single point mutation experiments** in ProteinGym.

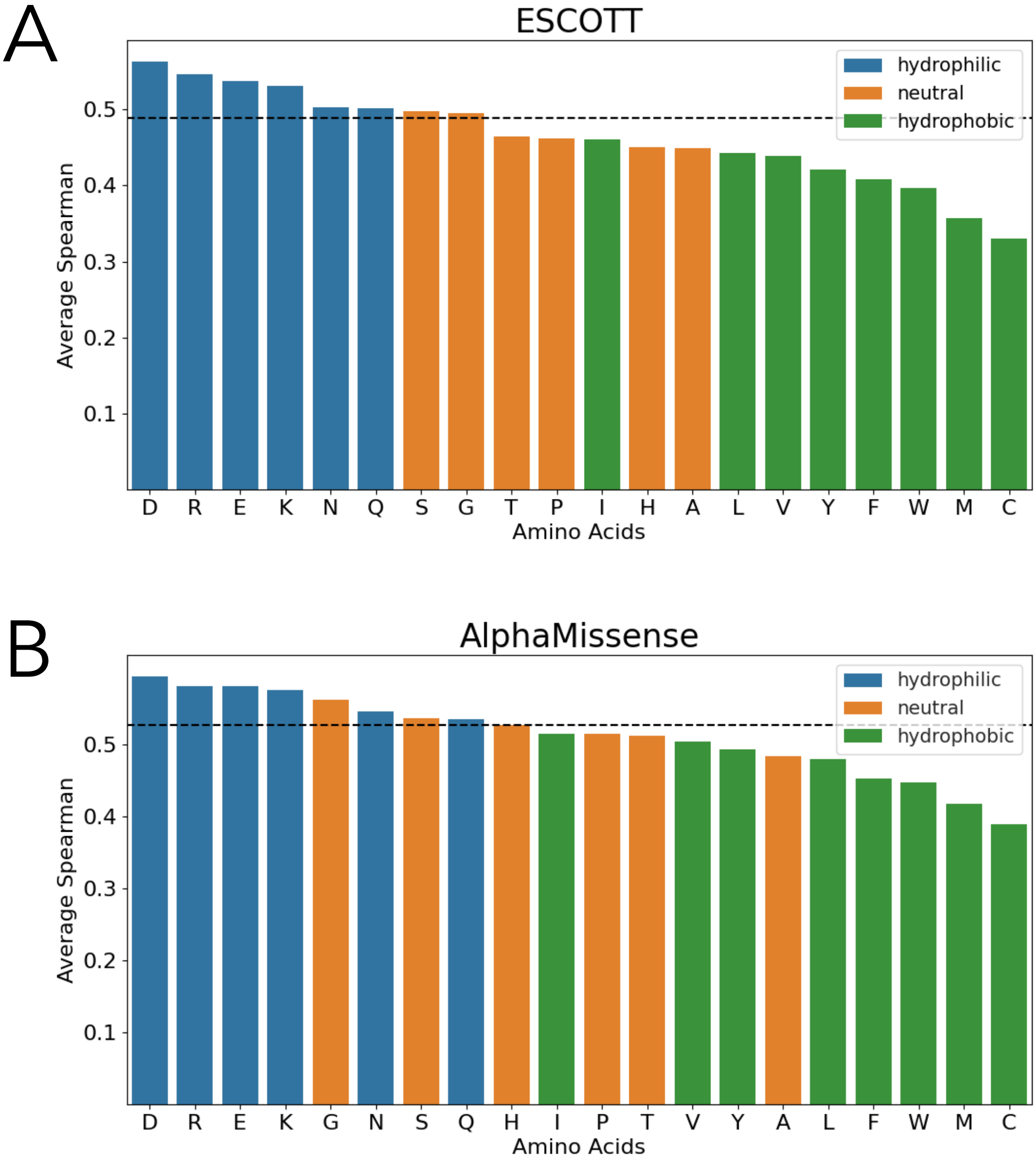

Fig S7. Average Spearman correlation for mutations towards the 20 specific amino acids. The amino acid bars are colored according to hydrophobicity. Colors follow the amino acid three-class partition by hydrophobicity used in https://weblogo.threeplusone.com/manual.html. **A.** ESCOTT analysis. The dashed line indicates the average Spearman correlation coefficient for ESCOTT (0.489) computed over the 76 single point mutation experiments. **B.** AlphaMissense analysis. The dashed line indicates the average Spearman correlation coefficient for AlphaMissense (0.527) computed over the 76 single point mutation experiments.

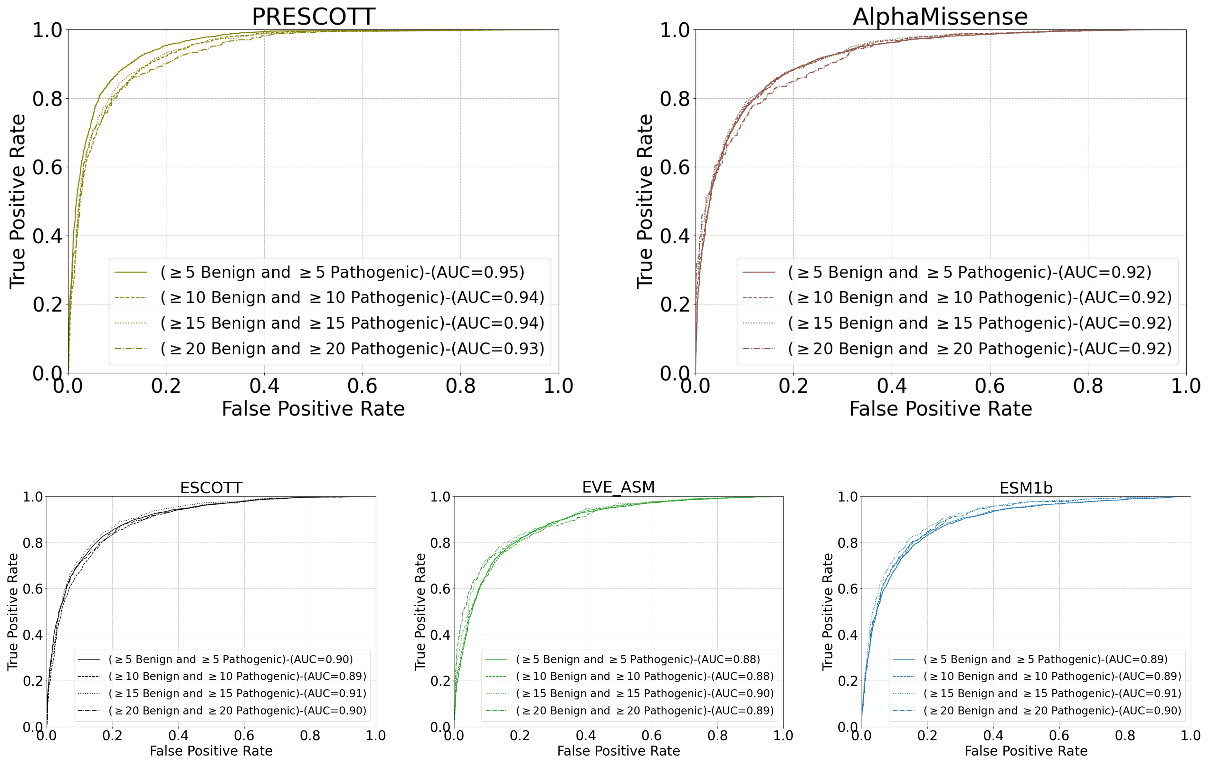

Fig S8. PRESCOTT's performance versus sample size, and comparison to other methods. Figure 7A analyses PRESCOTT 's performance across 1,883 proteins. Here, subsets with varying numbers of variants per protein are considered:

">=5 Benign and >=5 Pathogenic": 7,636 variants across 327 proteins;
">=10 Benign and >=10 Pathogenic": 3,734 variants across 96 proteins;
">=15 Benign and >=15 Pathogenic": 2,184 variants across 43 proteins;
">=20 Benign and >=20 Pathogenic": 1,642 variants across 26 proteins.

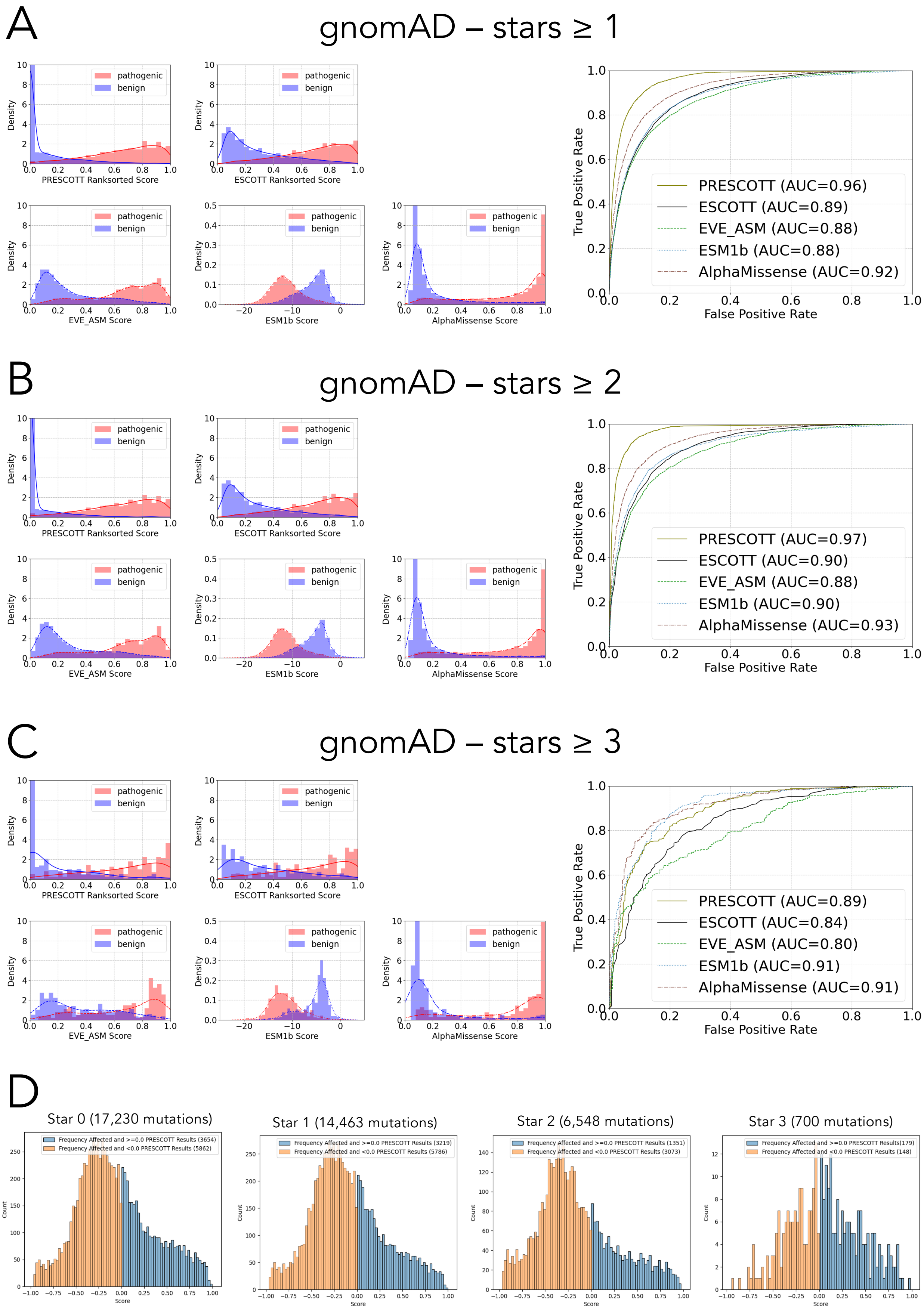

Fig S9. PRESCOTT performance on the dataset of 1,883 human proteins informed by gnomAD v4.0.0 population frequencies for different review statuses (stars). ABC. Compare to **Figure 7**. **D.** Distribution of PRESCOTT scores, prior to setting negative values to 0. The analysis includes only mutations whose scores were adjusted by allele frequency in PRESCOTT.

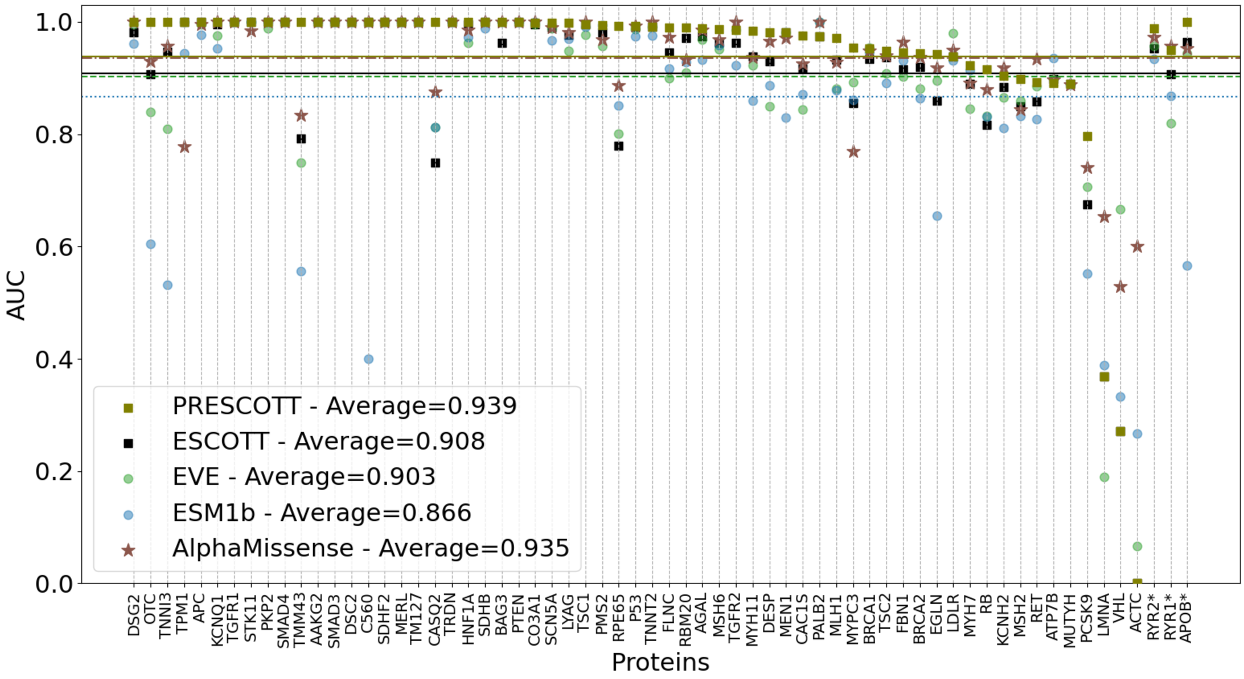

Fig S10. ACMG dataset analysis and comparison of PRESCOTT, ESCOTT, EVE and ESM1b for ACMG human proteins. Global comparative analysis, based on AUC scores, on 64 human proteins in the ACMG dataset of ESCOTT, PRESCOTT, EVE, ESM1b and AlphaMissense. For these proteins, where some are considered in **Figure 6A**, there is **at least 1 benign mutation** described in ClinVar.

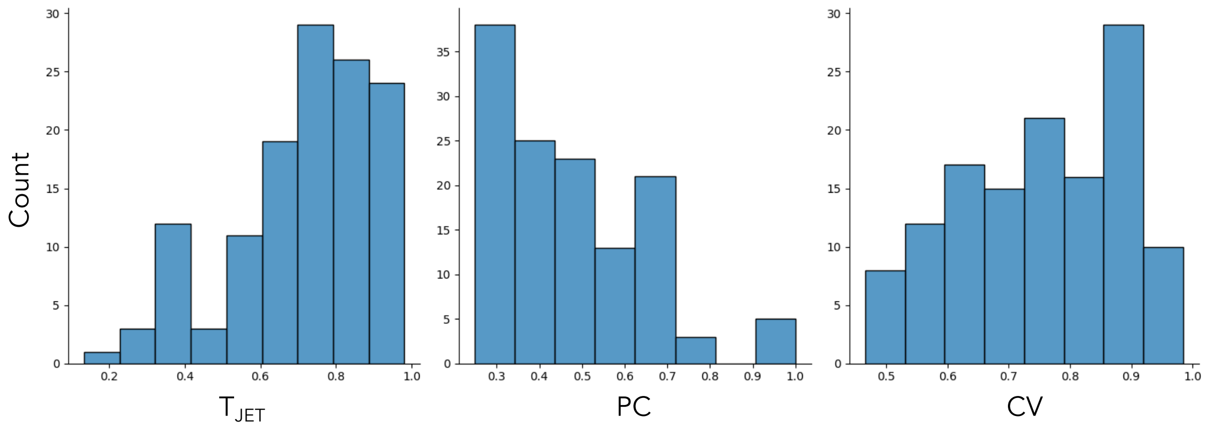

Fig S11. Feature analysis of the positions associated to gain-of-function mutations. Distribution of T_{JET}, PC and CV for the dataset of 128 gain-of-function mutations introduced in this work.

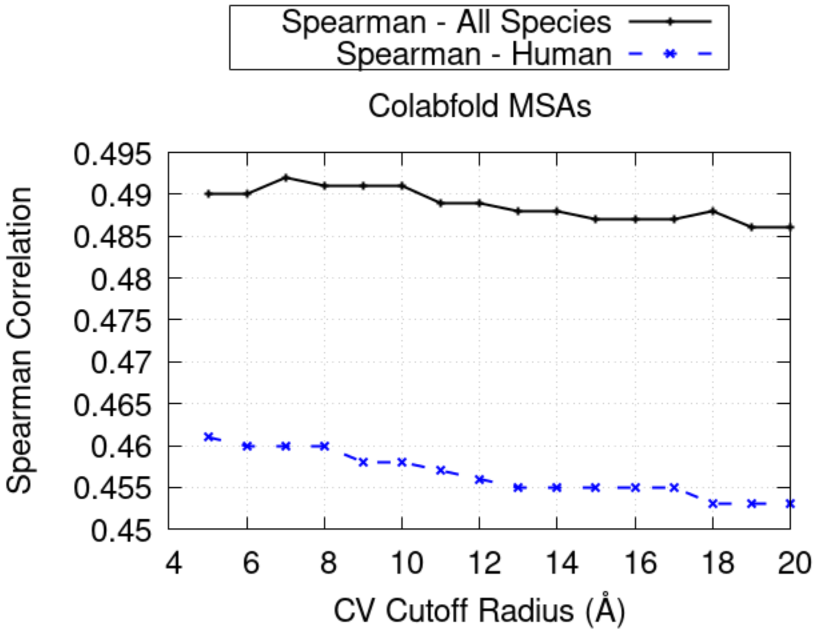

Fig S12. Analysis of ESCOTT performance based on the radius parameter used in the definition of CV. Spearman correlation coefficients computed for the entire set of DMS experiments (solid black line) and the human DMS experiments (dashed blue line) by varying values of the radius that defines the CV formula (x-axis).

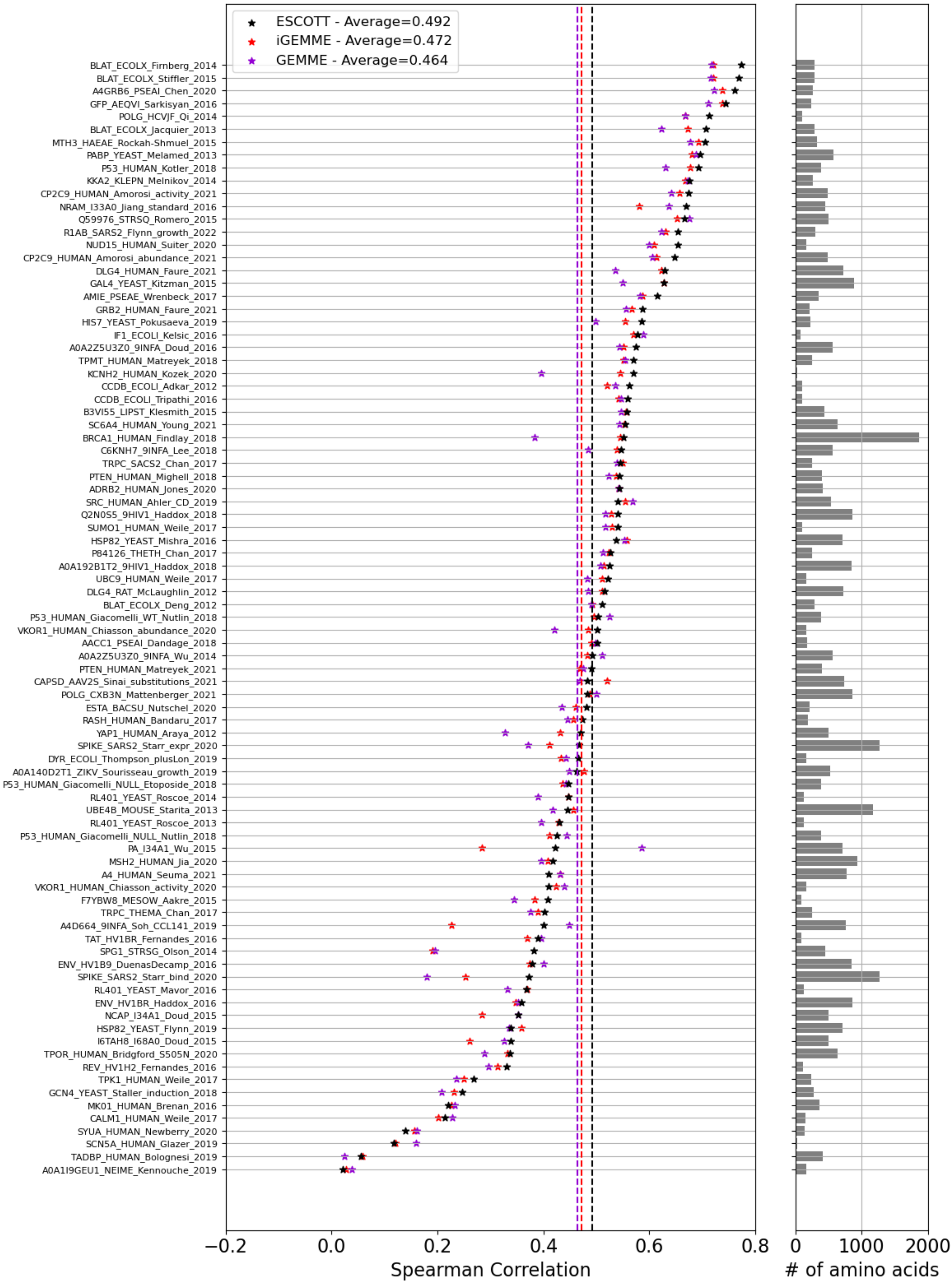

Fig S13. Comparison of ESCOTT, iGEMME and GEMME. Stars indicate the average Spearman correlation coefficient between predictions obtained with ESCOTT (black), iGEMME (red) and GEMME (purple) and the 87 experimental data of the ProteinGym dataset. Dashed lines indicate averages over the full dataset. Horizontal bars on the right show the number of amino acids in each protein.

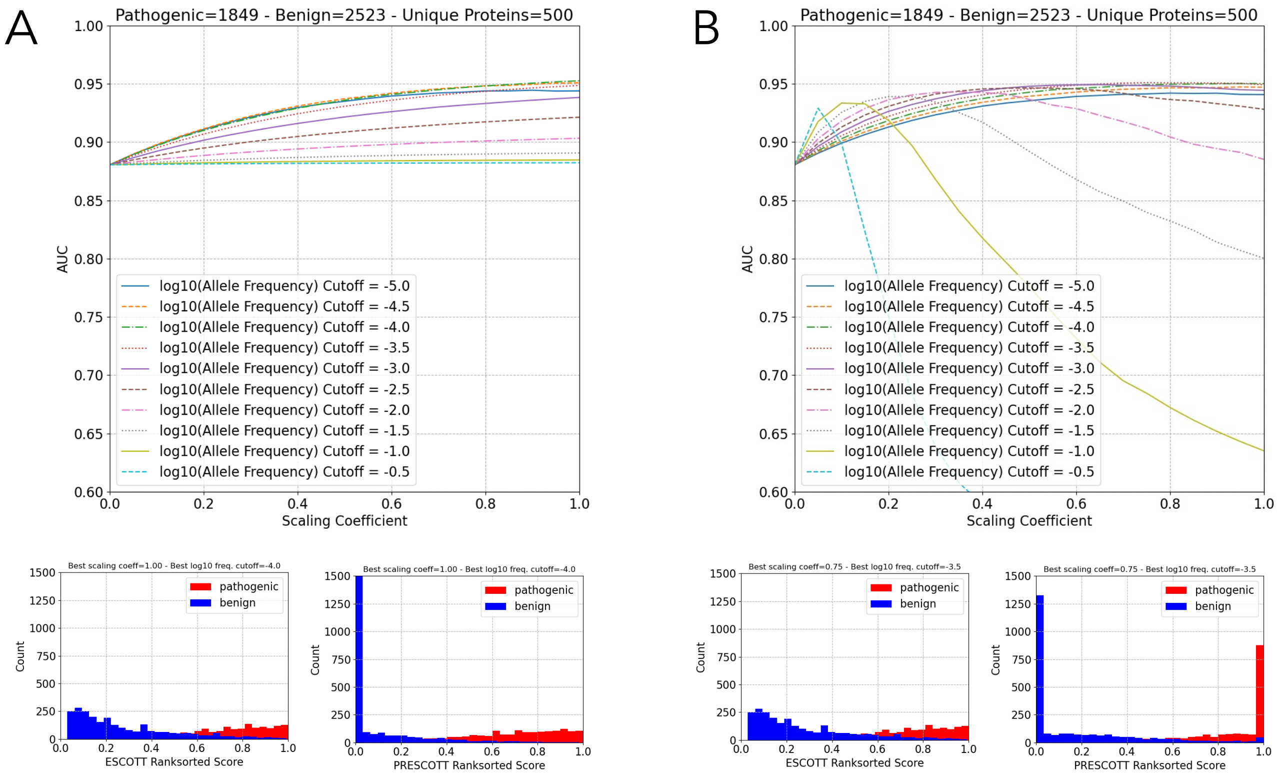

Fig S14. Analysis of the two parameters leading to an optimal PRESCOTT score. The analysis has been realised on 500 proteins containing 1849 pathogenic variants and 2523 benign ones. It is dedicated to fixing the default parameters for allele frequency cut-off and best scaling coefficient. **A.** Algorithm "PRESCOTTscore-high-allele-frequencies" in Methods. This model improves PRESCOTT scores for highly frequent mutations. Analysis of the allele frequency cut-off parameter (top) leading to a default value of -4 and a best scaling coefficient of 1. The distribution of ESCOTT (bottom left) and PRESCOTT (bottom right) scores is reported for pathogenic and benign variants in the 500 proteins. **B.** Algorithm "PRESCOTTscore-all-allele-frequencies" in Methods. This model improves PRESCOTT scores for both highly and lowly frequent mutations. The analysis of the allele frequency cut-off parameter (top) leads to a default value of -3.5 and a best scaling coefficient of 0.75. The distribution of ESCOTT (bottom left; same plot as in A) and PRESCOTT (bottom right) scores is reported for pathogenic and benign variants in the 500 proteins.

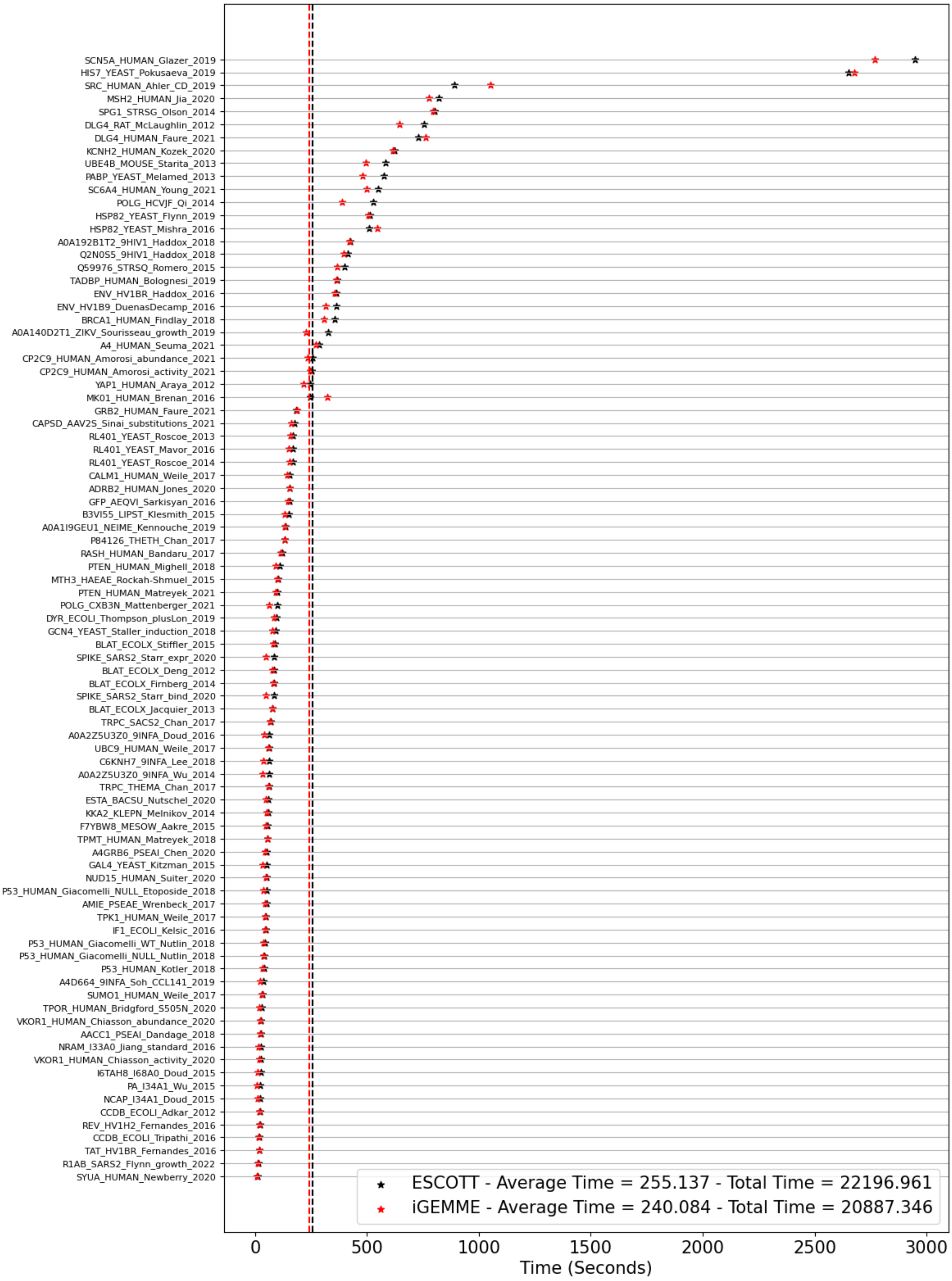

Fig S15. Computation time (in seconds) for all of the experiments in the ProteinGym dataset. The dashed lines indicate the average computing times for ESCOTT=320.531 secs (black) and iGEMME=268.784 secs (red) over 87 experiments.
