# Supplementary material for: PRESCOTT: a population aware, epistatic, and structural model accurately predicts missense effects
Source: Genome Biol. 2025 May 6;26:113. doi: 10.1186/s13059-025-03581-y (PMC12054230; doi:10.1186/s13059-025-03581-y)
Supplement: Supplementary file 2 — Additional file 2: Table S1—Mann–Whitney paired u-tests for data in Fig. 3A, Fig. 5A and Fig. 7C. [file 13059_2025_3581_MOESM2_ESM.docx]

| **Method 1** | **Method 2** | **p-value** | **statistic** |
| --- | --- | --- | --- |
| 1. Mann-Whitney paired u-test analysis of **Figure 3A** | | | |
| **ESCOTT** | EVE_ensemble | 0.0002 | 55.0000 |
| **ESCOTT** | ESM1b | 0.0008 | 92.0000 |
| **ESCOTT** | AlphaMissense | 0.5483 | 231.5000 |
| **EVE_ensemble** | ESM1b | 0.5119 | 227.5000 |
| **EVE_ensemble** | AlphaMissense | 0.0029 | 96.0000 |
| **ESM1b** | AlphaMissense | 0.0001 | 67.0000 |
| 1. Mann-Whitney paired u-test analysis of **Figure 6A** | | | |
| **ESCOTT Averaged** | EVE (Ensemble) Averaged | 0.0024 | 74.0000 |
| **ESCOTT Averaged** | ESM1b Averaged | 0.0089 | 89.5000 |
| **ESCOTT Averaged** | AlphaMissense Averaged | 0.0628 | 120.5000 |
| **EVE (Ensemble) Averaged** | ESM1b Averaged | 0.9019 | 196.5000 |
| **EVE (Ensemble) Averaged** | AlphaMissense Averaged | 0.1502 | 139.0000 |
| **ESM1b Averaged** | AlphaMissense Averaged | 0.0735 | 124.0000 |
| 1. Mann-Whitney paired u-test analysis of **Figure 7C** | | | |
| **PRESCOTT** | AlphaMissense | 0.0016 | 164.0000 |
| **PRESCOTT** | ESM1b | 0.0000 | 33.0000 |
| **PRESCOTT** | EVE | 0.0000 | 41.5000 |
| **PRESCOTT** | ESCOTT | 0.0000 | 5.5000 |
| **AlphaMissense** | ESM1b | 0.0000 | 74.0000 |
| **AlphaMissense** | EVE | 0.0000 | 92.0000 |
| **AlphaMissense** | ESCOTT | 0.0007 | 181.0000 |
| **ESM1b** | EVE | 0.1124 | 308.0000 |
| **ESM1b** | ESCOTT | 0.0005 | 173.0000 |
| **EVE** | ESCOTT | 0.0853 | 282.0000 |

**Table S1.** **Mann-Whitney paired u-tests for data in Figure 3A, Figure 5A and Figure 7C. A.** From the analysis of **Figure 3A**, ESCOTT emerges as a robust method, with significant performance advantages over EVE_ensemble and ESM1b, while maintaining parity with AlphaMissense. This highlights ESCOTT strong overall behavior and reliability across datasets, comparable only to AlphaMissense, which also consistently outperforms the other approaches. **B.** From the analysis of **Figure 6A**, ESCOTT demonstrates a better overall performance, as it shows statistically significant differences compared to EVE (Ensemble) and ESM1b, highlighting its robustness, while its comparable performance with AlphaMissense suggests it maintains competitive effectiveness even against strong alternatives. **C.** From the analysis of **Figure 7C**, PRESCOTT emerges as the strongest method, consistently showing statistically significant advantages over other approaches. AlphaMissense also demonstrates strong performance, particularly when compared to ESM1b and EVE. ESCOTT holds its ground against EVE but is clearly outperformed by PRESCOTT. This table highlights the robustness of PRESCOTT and AlphaMissense in this analysis. **C.**
