# Supplementary material for: PRESCOTT: a population aware, epistatic, and structural model accurately predicts missense effects
Source: Genome Biol. 2025 May 6;26:113. doi: 10.1186/s13059-025-03581-y (PMC12054230; doi:10.1186/s13059-025-03581-y)
Supplement: Supplementary file 4 — Additional file 4: Table S2—Analysis of 22 missense mutations associated with the Finnish Disease Heritage. [file 13059_2025_3581_MOESM4_ESM.docx]

| **Gene** | **Missense mutation** | **Mutation acronyme** | **UniprotID** | **ESCOTT score** | **PRESCOTT score** | **log10frequency**  **in gnomAD** | **Selected Population** | **AlphaMissense score** |
| --- | --- | --- | --- | --- | --- | --- | --- | --- |
| ***AGA*** | Cys163Ser | C163S | P20933 | 0.55 | 0.08 | -2.12 | European (Finnish) | 0.958 |
| ***PPT1*** | Arg122Trp | R122W | P50897 | 0.87 | 0.41 | -2.16 | European (Finnish) | 0.739 |
| ***KERA*** | Asn247Ser | N247S | O60938 | 0.82 | 0.41 | -2.37 | European (Finnish) | 0.882 |
| ***CRADD*** | Arg170His | R170H | P78560 | 0.21 | 0.00 | -2.23 | European (Finnish) | 0.082 |
| ***CLN8*** | Arg24Gly | R24G | Q9UBY8 | 0.91 | 0.65 | -2.97 | European (Finnish) | 0.615 |
| ***GSN*** | Asp214Asn | D214N | P06396 | 0.60 | 0.60 | No frequency |  | 0.719 |
| ***GLDC*** | Ser564Ile | S564I | P23378 | 0.84 | 0.52 | -2.73 | European (Finnish) | 0.960 |
| ***BCS1L*** | Ser78Gly | S78G | Q9Y276 | 0.48 | 0.08 | -2.41 | European (Finnish) | 0.253 |
| ***HYLS1*** | Asp211Gly | D211G | Q96M11 | 0.96 | 0.47 | -2.05 | European (Finnish) | 0.973 |
| ***OAT*** | Leu402Pro | L402P | P04181 | 0.94 | 0.59 | -2.59 | European (Finnish) | 0.995 |
| ***TWNK*** | Tyr508Cys | Y508C | Q96RR1 | 0.68 | 0.32 | -2.58 | European (Finnish) | 0.191 |
| ***FSHR*** | Ala189Val | A189V | P23945 | 0.62 | 0.16 | -2.17 | European (Finnish) | 0.386 |
| ***PCK1*** | Gly309Arg | G309R | P35558 | 0.94 | 0.43 | -1.95 | European (Finnish) | 0.997 |
| ***ZNHIT3*** | Ser31Leu | S31L | Q15649 | 0.96 | 0.55 | -2.36 | European (Finnish) | 0.743 |
| ***RS1*** | Glu72Lys | E72K | O15537 | 0.64 | 0.64 | -4.37 | European (Finnish) | 0.918 |
| ***SLC17A5*** | Arg39Cys | R39C | Q9NRA2 | 0.93 | 0.52 | -2.34 | European (Finnish) | 0.968 |
| ***CHCHD10*** | Gly66Val | G66V | Q8WYQ3 | 0.52 | 0.52 | -4.32 | European (Finnish) | 0.960 |
| ***TK2*** | Arg183Trp | R183W | O00142 | 0.74 | 0.51 | -3.09 | European (Finnish) | 0.293 |
| ***AICDA*** | Met139Thr | M139T | Q9GZX7 | 0.92 | 0.60 | -2.73 | European (Finnish) | 0.987 |
| ***HADHA*** | Glu510Gln | E510Q | P40939 | 0.73 | 0.32 | -2.36 | European (Finnish) | 0.986 |
| ***POLG*** | Trp748Ser | W748S | P54098 | 0.45 | 0.00 | -2.20 | European (Finnish) | 0.766 |
| ***CUBN*** | Pro1297Leu | P1297L | O60494 | 0.77 | 0.39 | -2.50 | European (Finnish) | Not computed |

**Table S2. Analysis of 22 missense mutations associated with the Finnish Disease Heritage.** The table lists 22 missense mutations linked to FDH as reported by (Uusimaa *et al* 2022). ESCOTT scores (fifth column) are available in our online database for the human proteome: ESCOTT database (<http://prescott.lcqb.upmc.fr/prescott_data_main.php>). The only exception is mutation P1297L in the *CUBN* gene, due to protein’s length (3,623aa). AlphaMissense scores were retrieved from the AlphaFold Database (AFDB), where gene *CUBN* is not reported. No allele frequency is reported in the gnomAD database for the GSN gene. Mutations for ESCOTT and PRESCOTT are classified with an upper bound of 0.28 for benign mutations and a lower bound of 0.42 for pathogenic ones. Mutations for AlphaMissense are classified with thresholds 0.34 and 0.56 established in (Cheng et al. 2023). Color code for predictions: pathogenic (orange), VUS (dark yellow), benign (light yellow).
