# Supplementary material for: PRESCOTT: a population aware, epistatic, and structural model accurately predicts missense effects
Source: Genome Biol. 2025 May 6;26:113. doi: 10.1186/s13059-025-03581-y (PMC12054230; doi:10.1186/s13059-025-03581-y)
Supplement: Supplementary file 5 — Additional file 5: Table S3—Analysis of the STAT1 gene with ESCOTT, PRESCOTT, iGEMME and AlphaMissense. [file 13059_2025_3581_MOESM5_ESM.docx]

| **Mutation** | **Mode** | **ESCOTT** | **PRESCOTT** | **AlphaMissense** | **iGEMME** |
| --- | --- | --- | --- | --- | --- |
| D165H | GF | 0.55 | 0.55 | 0.957 | 0.58 |
| D165G | GF | 0.55 | 0.55 | 0.905 | 0.58 |
| Y170N | GF | 0.86 | 0.86 | 0.725 | 0.86 |
| C174R | GF | 0.27 | 0.27 | 0.203 | 0.27 |
| N179K | GF | 0.24 | 0.24 | 0.657 | 0.23 |
| M202V | GF | 0.18 | 0.18 | 0.386 | 0.17 |
| A267V | GF | 0.5 | 0.5 | 0.8919 | 0.49 |
| Q271P | GF | 0.81 | 0.81 | 0.96 | 0.83 |
| R274Q | GF | 0.56 | 0.56 | 0.326 | 0.53 |
| R274G | GF | 0.68 | 0.68 | 0.851 | 0.66 |
| R274W | GF | 0.58 | 0.58 | 0.527 | 0.56 |
| K278E | GF | 0.31 | 0.31 | 0.61 | 0.36 |
| Q285R | GF | 0.38 | 0.38 | 0.325 | 0.49 |
| K286I | GF | 0.48 | 0.48 | 0.964 | 0.56 |
| T288A | GF | 0.15 | 0.15 | 0.226 | 0.19 |
| E320Q | AD | 0.55 | 0.55 | 0.979 | 0.59 |
| G384D | GF | 0.54 | 0.54 | 0.99 | 0.68 |
| T385M | GF | 0.82 | 0.82 | 0.68 | 0.89 |
| Q463H | AD | 0.82 | 0.82 | 0.999 | 0.9 |
| L600P | AR | 0.99 | 0.99 | 0.999 | 1.0 |
| K637R | AD | 0.06 | 0.06 | 0.085 | 0.08 |
| K637E | AD | 0.23 | 0.23 | 0.749 | 0.32 |
| L706S | AD | 0.56 | 0.56 | 0.999 | 0.55 |

**Table S3. Analysis of the STAT1 gene with ESCOTT, PRESCOTT, iGEMME and AlphaMissense.** 23 mutations with demonstrated pathogenic effect in STAT1. Inheritance modes: gain of function (GF; dark green), autosomal recessive (AR; fluorescent green), autosomal dominant (AD; light green). Predictions: pathogenic (dark yellow), VUS (light yellow), benign (white). Mutations for ESCOTT, PRESCOTT and iGEMME are classified with an upper bound of 0.28 for benign mutations and a lower bound of 0.42 for pathogenic ones. Mutations for AlphaMissense are classified with thresholds 0.34 and 0.56 established in (Cheng et al. 2023).
