# Supplementary material for: PRESCOTT: a population aware, epistatic, and structural model accurately predicts missense effects
Source: Genome Biol. 2025 May 6;26:113. doi: 10.1186/s13059-025-03581-y (PMC12054230; doi:10.1186/s13059-025-03581-y)
Supplement: Supplementary file 6 — Additional file 6: Table S4-Analysis of the PTPN11 gene with ESCOTT, PRESCOTT, iGEMME and AlphaMissense. [file 13059_2025_3581_MOESM6_ESM.docx]

| **Mutation** | **Mode** | **ESCOTT** | **PRESCOTT** | **AlphaMissense** | **iGEMME** |
| --- | --- | --- | --- | --- | --- |
| T42A | GF | 0.38 | 0.38 | 0.973 | 0.33 |
| N58D | GF | 0.59 | 0.59 | 0.994 | 0.52 |
| N58H | GF | 0.46 | 0.46 | 0.978 | 0.41 |
| N58K | GF | 0.55 | 0.55 | 0.999 | 0.48 |
| D61N | GF | 0.13 | 0.13 | 0.994 | 0.13 |
| D61G | GF | 0.52 | 0.52 | 0.999 | 0.43 |
| D61A | GF | 0.68 | 0.68 | 0.999 | 0.6 |
| Y62D | GF | 0.62 | 0.62 | 0.994 | 0.49 |
| Y62C | GF | 0.13 | 0.13 | 0.804 | 0.13 |
| Y63C | GF | 0.39 | 0.39 | 0.975 | 0.35 |
| E69Q | GF | 0.44 | 0.44 | 0.990 | 0.43 |
| A72S | GF | 0.35 | 0.35 | 0.916 | 0.29 |
| A72G | GF | 0.7 | 0.7 | 0.993 | 0.6 |
| E76D | GF | 0.44 | 0.44 | 0.998 | 0.4 |
| Q79R | GF | 0.44 | 0.44 | 0.964 | 0.4 |
| D106A | GF | 0.45 | 0.45 | 0.985 | 0.41 |
| E139D | GF | 0.42 | 0.42 | 0.988 | 0.44 |
| Q256R | GF | 0.4 | 0.4 | 0.894 | 0.39 |
| L261F | GF | 0.18 | 0.18 | 0.507 | 0.18 |
| L261H | GF | 0.31 | 0.31 | 0.507 | 0.27 |
| L262R | GF | 0.45 | 0.45 | 0.926 | 0.44 |
| R265Q | GF | 0.19 | 0.19 | 0.994 | 0.24 |
| Y279S | DN | 0.86 | 0.86 | 0.999 | 0.88 |
| I282V | GF | 0.57 | 0.57 | 0.981 | 0.58 |
| F285S | GF | 0.89 | 0.89 | 0.999 | 0.88 |
| F285L | GF | 0.78 | 0.78 | 0.999 | 0.8 |
| N308D | GF | 0.76 | 0.76 | 0.996 | 0.78 |
| N308S | GF | 0.33 | 0.33 | 0.518 | 0.35 |
| G409A | GF | 0.15 | 0.15 | 0.261 | 0.19 |
| T411M | GF | 0.12 | 0.12 | 0.065 | 0.12 |
| A461T | GF | 0.16 | 0.16 | 0.999 | 0.2 |
| A461S | DN | 0.52 | 0.52 | 0.972 | 0.58 |
| G464A | DN | 0.91 | 0.91 | 0.999 | 0.92 |
| T468M | DN | 0.83 | 0.83 | 0.994 | 0.84 |
| T468P | DN | 0.99 | 0.99 | 0.996 | 0.99 |
| P491T | GF | 0.49 | 0.49 | 0.896 | 0.5 |
| P491S | GF | 0.29 | 0.29 | 0.798 | 0.27 |
| P491H | GF | 0.17 | 0.17 | 0.961 | 0.16 |
| P491L | GF | 0.28 | 0.28 | 0.943 | 0.27 |
| R498W | DN | 0.92 | 0.92 | 0.999 | 0.93 |
| R498L | DN | 0.94 | 0.94 | 0.999 | 0.95 |
| R501K | GF | 0.83 | 0.83 | 0.997 | 0.85 |
| S502T | GF | 0.69 | 0.69 | 0.984 | 0.68 |
| S502A | GF | 0.52 | 0.52 | 0.869 | 0.53 |
| S502L | GF | 0.51 | 0.51 | 0.998 | 0.51 |
| G503R | GF | 0.66 | 0.66 | 0.999 | 0.66 |
| G503E | GF | 0.21 | 0.21 | 0.999 | 0.24 |
| G503V | GF | 0.78 | 0.78 | 0.999 | 0.78 |
| G503A | GF | 0.72 | 0.72 | 0.998 | 0.72 |
| M504V | GF | 0.71 | 0.71 | 0.983 | 0.73 |
| Q506P | DN | 0.92 | 0.92 | 0.999 | 0.93 |
| T507K | DN | 0.43 | 0.43 | 0.996 | 0.49 |
| Q510R | DN | 0.92 | 0.92 | 0.999 | 0.93 |
| Q510E | DN | 0.76 | 0.76 | 0.943 | 0.81 |
| Q510P | DN | 0.96 | 0.96 | 0.998 | 0.96 |
| Q510H | DN | 0.91 | 0.91 | 0.999 | 0.92 |
| T553M | GF | 0.06 | 0 | 0.07 | 0.06 |
| L560F | GF | 0.1 | 0.02 | 0.125 | 0.12 |

**Table S4. Analysis of the PTPN11 gene with ESCOTT, PRESCOTT, iGEMME and AlphaMissense.** 58 mutations with demonstrated pathogenic effect. Inheritance modes: gain of function (GF; dark green), dominant negative (DN; light green). Predictions: pathogenic (dark yellow), VUS (light yellow), benign (white). Mutations for ESCOTT, PRESCOTT and iGEMME are classified with an upper bound of 0.28 for benign mutations and a lower bound of 0.42 for pathogenic ones. Mutations for AlphaMissense are classified with thresholds 0.34 and 0.56 established in (Cheng et al. 2023).
