# Supplementary material for: PRESCOTT: a population aware, epistatic, and structural model accurately predicts missense effects
Source: Genome Biol. 2025 May 6;26:113. doi: 10.1186/s13059-025-03581-y (PMC12054230; doi:10.1186/s13059-025-03581-y)
Supplement: Supplementary file 8 — Additional file 8: Review history. [file 13059_2025_3581_MOESM8_ESM.docx]

Review History

**First round of review**

**Reviewer 1**

**Were you able to assess all statistics in the manuscript, including the appropriateness of statistical tests used?** Yes: The stats are overall appropriate in this article. Areas for improvement and clarification are detailed in the comments to author.

**Were you able to directly test the methods?** Yes

**Comments to author:**

In this article, the authors proposed a new computational approach, called PRESCOTT, to predict the effects of missense variants. PRESCOTT integrates three major biological information: evolutionary conservation, protein structural importance, and population allele frequency. The authors demonstrated the superior performance of PRESCOTT (and its precursor ESCOTT) to other metrics in predicting pathogenic variants using a few mutation datasets. They also built a user-friendly website to facilitate the application of their methods as well as the sharing of precomputed results. While the proposed framework overall appears promising, a series of improvements and/or clarifications need to be made.

1. Based on the Methods section, the TJET value comes from iGEMME, but it is not clear 1) in the Main text that TJET is a metric developed previously and 2) what improvement iGEMME has been made over GEMME. It would be helpful to make these explicit to the readers, highlighting which aspects of the proposed method are new and which build on prior work.
2. Among the three components in PRESCOTT, two are mutation-specific (conservation and allele frequency) while the protein structural fractures are at the residue level. What are the authors' perspectives on adding mutation-specific information to CV and PC - for instance, CV(mutatnt)-CV(wild-type)? Would that be technically feasible under the current framework?
3. The authors focused on two protein structural features - the core and the interface. Albeit important, functional regions are not necessarily located at the core or the interface, like certain ligand/small molecule biding pockets. While some of the important motifs can be captured by TJET, non-core/interface regions would still be deprioritized by CV and PV.
4. The selection of CV and PV as the sole measurements for each feature could benefit from incorporating additional or more recent metrics. For example, the solvent-accessible surface area (SASA) score can complement CV; and for PV, there are many new approaches predicting protein interaction interfaces - I would highly encourage the authors to consider incorporating these advancements.
5. While this may not be the authors' intension, the introduction on CV and PV referenced their utilities in measuring loss-of-function (pg3 line 1-2,5-6). Are such metrics also able to capture gain-of-function, especially given that the authors later highlighted the strength of their approaches in predicting gain-of-function effects?
6. The authors illustrated in Figure 1F "for a position i occupied by residue P in q, the distance within the tree to the closest sequence, say s, displaying R at position i. The intuition is that if s is located far from q in the tree, the P-to-R mutation should require a large number of mutations in q for R to be "accepted" and therefore P-to-R should probably be deleterious". I wander whether there are cases where multiple sequences/subtrees display R, and will that create more chances for R to be "accepted"? Would considering the number of sequences/subtrees and their distances (rather than the min distance) improve the estimation of TJET?
7. PRESCOTT selects the popmax allele frequency based on the assumption that "if a variant is common in one population, it can be assumed to be benign across all populations". However, this assumption may not hold well - for instance, the Finnish population has a unique genetic heritage due to its historical isolation and population bottlenecks, which has led to the enrichment of certain pathogenic variants, known as the Finnish Disease Heritage (FDH). FDH comprises nearly 40 rare monogenic diseases that are over-represented in Finland but rare elsewhere [PMID: 36285626]. Relatedly, how often / what are the cases where ESCOTT outperforms PRESCOTT?
8. It is exciting that the proposed approach can be applied to a diverse range of "large proteins, intrinsically disordered regions, splicing variants, mutated sequences, and artificially generated sequences". Could the authors provide some insights into the relative performance (like AUCs) of PRESCOTT/ESCOTT within each of these categories? This would help illustrate the strengths and limitations of the approach across diverse use cases.
9. Similarly, the authors tested the performance of ESCOTT/PRESCOTT on 32 human protein DMS experiments and its generality across species on all the 87 DMS experiments. Does ESCOTT/PRESCOTT perform better in human proteins vs other species, or any specific classes of proteins? For example, the authors already observed variable performance across different proteins - with Spearman correlation coefficients ranging from 0.135 for SCN5A to 0.690 for P53 - understanding the underlying reasons would be important for the reader to better interpret the approach as well as the results.
10. In the comparison of performance, the authors used average values of Spearman correlation coefficient. It would be useful to supply a measurement of the significance of improvement. For instance, the authors could consider using a paired u-test to evaluate the differences shown in Figure 3A, 5A, etc.
11. It is interesting that some regions not well modeled by AlphaFold are recognized by both ESCOTT and AlphaMissense. I wonder how low-quality structural regions are handled when computing structural features (CV and PV)?
12. As the author stated about Table 1, "AlphaMissense outperforms all methods. Yet it is important to note that its advantage partly stems from being explicitly finetuned to distinguish between pathogenic and benign effects. In contrast, ESCOTT, along with the other comparative methods, does not utilize classified data in its modeling process". Is there a particular reason for the many other approaches avoid training on pathogenic vs benign effects? If so, it would be critical to elaborate on scenarios where such a strategy is superior to AlphaMissense (which may fail to recognize certain pathogenic features that were not included in its training data?)
13. The authors showed that ESCOTT outperforms AlphaMissense in predicting "sensitive regions", which is encouraging. But this seems to hold true only for the 28 DMS (Figure 5A) but not all DMS, as shown in Figure S3A? It may be worth clarifying in the main text.
14. The section "Some global observations lead to an improved interpretation of ESCOTT scores" provides important information about the properties of the proposed approach. I would suggest 1) include AlphaMissense in the comparisons shown in Figure S4 and S5 and 2) elaborating in the Discussion on possible underlying reasons for these properties, e.g., why it predicts mutations toward hydrophilic residues (D, R, E, K, N, Q) particularly well?
15. It is impressive that PRESCOTT achieved a high AUC on both the ACMG proteins and the 1,883 gnomAD proteins. I wonder how sensitive PRESCOTT's performance is, compared to other methos, to the sample size (i.e., the number of variants evaluated per protein)?
16. The ability of ESCOTT/PRESCOTT to identify gain-of-function is particularly exciting. But I assume that the final score does not distinguish between loss- and gain-of-function (?) It would be interesting to explore whether some intermediate scores could indicate loss- vs gain-of-function effects.
17. For the gain-of-function evaluation, the authors created a new dataset of gain-of-function mutations, integrating several data sources including "unpublished experimental data". For greater transparency, I would suggest performing a separate analysis using only published evidence.
18. In the section "Classification of Human Variants as Benign, Pathogenic …", more details and statistics are expected to describe how categories shift under PRESCOTT compared to ESCOTT. Additionally, it would be desirable to show that variants reclassified from benign to pathogenic are biologically significant - e.g., are enriched for certain functional annotations.
19. In the same section, the authors stated that "ESCOTT's accurate predictions for pathogenic variants are achieved without relying on allele". This could be confusing, given that that PRESCOTT is designed to improve ESCOTT in classifying pathogenic vs. benign variants. It would be helpful to provide insights into how one should choose between ESCOTT and PRESCOTT when their predicted categories differ.
20. The authors found that the analysis of pathogenic mutations in the MEFV protein is particularly challenging for both ESCOTT/PRESCOTT and AlphaMissense systems. Could the author discuss potential reasons and ways of improvement in Discussion?
21. In the Methods section, the selection of certain thresholds could benefit from further justification or explanation: 1) N=500 proteins for calibrating default parameter values in ESCOTT; 2) α=0.6 as the linear coefficient in the ESCOTT model; additionally, it is not very clear how the relative frequencies of Yi is defined.
22. Given the frequencyCutoff=-4, the minimum sample size to reach a low frequency of 0.0001 is 10,000 individuals. Therefore, it would be more logical to consider only populations with more than 10,000 individuals. For example, in the Middle Eastern population with N=3031, the occurrence of just one mutant allele would result in a "high" frequency and make it downweighed for pathogenicity. However, such variants are still likely to be deleterious (especially if it remains low frequency in other larger populations). I would thus suggest excluding the Middle Eastern population from the analysis avoid potential bias in the results.
23. While the fixation of PRESCOTT values at a min of 0 makes it easier for score interpretation, it would be worth illustrating to the readers how often or to what degree the scores could fall below 0 (in relation to allele frequency if this restriction were not applied).
24. The two sub-models of PRESCOTT - PRESCOTTscore-high-allele-frequencies and PRESCOTTscore-all-allele-frequencies - may provide complementary information. It would be valuable to perform similar evaluation tests to compare the performance/different utilities of these two models. Perhaps relatedly, the authors mentioned that "The frequency of gnomAD alleles in the Star >0 set, considering all populations at once, was tested, but performance was slightly inferior (0.94)". But it is unclear what specific performance metric was being measured in this context.
25. The section "Analysis of the independent and epistatic modules building ESCOTT" in the Methods is interesting - It would be worth elaborating on and moving it to the main text.

**Reviewer 2**

**Were you able to assess all statistics in the manuscript, including the appropriateness of statistical tests used?** Yes: I have been able to review this manuscript to the best of my abilities, especially in terms of protein/molecular structure, evolutionary conservation, (systematic) missense variant interpretation. software development/engineering, general scientific method, etc. However, I would recommend having at least one reviewer with a stronger expertise on machine learning model development (page 14 and 15), to provide a full picture on the soundness of all parts of this paper.

**Were you able to directly test the methods?** Yes

**Comments to author:**

Tekpinar, et al. introduces a novel method for predicting the effects of missense mutations, elegantly wrapped in three resources: the ESCOTT web server, ESCOTT Database and PRESCOTT web server. Their method integrates evolutionary conservation, molecular structures, and population variant frequency. Doing so it surpasses existing state-of-the-art computational approaches, including AlphaMissense and ESM1b in a comprehensive comparison evaluated on DMS experiments (e.g. Proteingym). Throughout the manuscript it quickly becomes clear that the largest similarities lie between PRESCOTT and AlphaMissense. Tekpinar et al. can surpass predictive metrics compared to AlphaMissense (although by a slim margin) likely due to the author's different way of incorporating evolutionary conservation and in the way they incorporate population frequency of variants. There are two central novelties in Tekpinar et al.'s approach: Firstly they provide a understandable non-black box type of approach as to why a variant is marked as benign-vus-pathogenic. Secondly, they outperform all other state-of-the-art methods by a small margin.

I would like the congratulate the authors with the tremendous amount of work that is contained within the manuscript and the extra effort to make it usable for the clinical and research community through their webserver. Additionally, I am very happy the authors thought of reproducibility by using publicly available containerization, code and data repositories. I do have the following unclarities that I hope the authors can address followed by a few minor comments.

Impact of structure and absence of structure on PRESCOTT predictions. Fig 4 shows a strong correlation that when structural confidence (by AlphaFold's pLDDT) goes down, so do the pathogenic predictions for all compared models here. However, AlphaMissense seems to still predict pathogenicity in these areas, the yellow dots, which do not correspond to ESCOTT or the other models. I am curious how ESCOTT/PRESCOTT improves on AlphaMissense in the regions without high confidence structures versus the regions with. This comparison would be more informative then just an overal comparison as provided in the manuscript as this would show in what situation PRESCOTT/ESCOTT improves beyond AlphaMissense. In addition, the manuscript could use a better description on how the authors exactly incorporate the pLDDT confidence values of predicted protein structures.

Using gnomAD to infer if variation is benign. Page 4: Line 22-23 "As a general rule, if a variant is common in one population, it can be assumed to be benign across all populations." Is not true for historical population bottlenecks that inflated variant frequencies in some populations (s.a. the Finnish) and it does not consider pathogenicity through recessive inheritance. The authors should clarify how PRESCOTT/ESCOTT, if at all, deals with pathogenicity through different modes of inheritence.

Interpretability of the ESCOTT/PRESCOTT scores. Figure 2 D&F: Unsure what the score scale means in D and F, between ESCOTT and PRESCOTT, would be very helpful for non-CS/Math oriented clinical experts to better describe this so they can understand it in context for their patients; e.g. what does a score of 0 or of 1 indicate PRESCOTT and ESCOTT? Is that scoring (0,1) different in the ESCOTT/PRESCOTT score scale? I am also unclear what table F exactly tries to convey because of this and cannot accurately understand from this table the impact of adding allele frequency on the scoring. Adding a row that show what you expect it to be for a perfect Mutation scoring would help here.
I have the following minor comments:
* The manuscript could use an overall restructuring for better readability. the dataset, model development, and comparisons to other computational methods are scattered throughout the manuscript rather than being organized in fewer, more focused sections and PRESCOTT and it's capabilities is mentioned very often and need not be repeated so often. A larger font in the figures (on y/x axis and text throughout) could also greatly improve the readability.
* Please add the UniProt identifiers to their respective protein to the gain of function mutations .csv file so no confusion can be caused regarding isoform/canonical sequence should future researchers require this for comparison.
* ESCOTT is mentioned that it can be applied to splicing variants in line 61 page 4, but thereafter no mention is made on how ESCOTT can help with evaluating such variants.
* Page 13, the note on ClinVar is a bit confusing and seems to indicate that PRESCOTT cannot deal with One-Based Vs. Zero-Based Genome Coordinate Systems, but could also mean this is only for the sake of comparison to EVE? Clarification would help here.
* Use of word doctors throughout manuscript (p4 line 14 and p16 line 10) where the authors are referring to target users. I believe a better word for inclusivity would be clinicians (as, worldwide, not all GCs or clinical researchers are also MDs/doctors)
* Minor Docker/gitlab issues: I could not get the docker / gitlab make a test-run in a fluent way. There were a few minor issues in the way. Firstly, There's no latest tag in dockerhub, making ` docker pull tekpinar/prescott-docker` or ` docker pull tekpinar/prescott-docker:latest` result in an error. Would recommend to add a `latest` tag in dockerhub and specify the version within the manuscript that was used for the results (I think. v.1.6.0?). Had to use `docker pull tekpinar/prescott-docker:v1.6.0`. Next I cloned the repository from gitlab but it would be helpful to add Docker details for end users to run your example. Instead of the command `prescott -e ../data/MLH1_normPred_evolCombi.txt -g ../data/gnomAD_v4.0.0_MLH1_HUMAN_ENSG00000076242.csv -s ../data/MLH1.fasta` I had to modify the docker run executed within the cloned PRESCOTT project as
`docker run --mount src="$(pwd)/data",target=/data,type=bind tekpinar/prescott-docker:v1.6.0 prescott -e /data/MLH1_normPred_evolCombi.txt -g /data/gnomAD_v4.0.0_MLH1_HUMAN_ENSG00000076242.csv -s /data/MLH1.fasta`. Would like to note this is cherry-on-top type of a minor comment, the authors really did an excellent job on their documentation and reproducibility.
* Page 15, line 22: states Mustafa - likely a typo Based on author's name.

**Authors Response**

Dear Dr Cosgrove,

Thank you for giving us the opportunity to address the reviewer comments. We would like to extend our sincere gratitude to you and the reviewers for their thoughtful and insightful feedback. The reviews were particularly stimulating, leading to additional analyses that have enhanced our results, improved clarity and, we hope, increased the overall impact of our work. We wish to emphasize our deep appreciation for the reviewers' perceptive remarks and their thorough understanding of the subject. Engaging with their comments and incorporating their suggestions was both a rewarding challenge and a pleasure.

We have carefully followed all recommendations from the two reviewers and provided a detailed, point-by-point response to each comment. As a result, the manuscript has undergone significant improvements. Below, we highlight the most important changes:

as requested in your letter:

1. we have uploaded **PRESCOTT software** **in** **zenodo**, as you requested, and we have linked it to the following DOI number: <https://doi.org/10.5281/zenodo.14506196>
2. We updated the software to render accessible to the user, PRESCOTT intermediate scores, T_JET_, CV and PC, as well as the population origin of the allele frequency data used in score adjustment. The update was applied to the webserver as well.
3. The **abstract** is under 100 words.
4. The **software licence** is cited in the article “open source CC-BY-NC-SA 4.0 license.”
5. The section “**Availability of data and material**” includes all links to data and software generated and developed in this study. See **pp 24**.

to answer to various comments from the referees, we added new Figures or improved existing ones:

6. We provided a new case study on **isoforms** showing that ESCOTT/PRESCOTT is able to distinguish mutational effects in isoforms. A case study on the human *TP53* shows the finding and highlights the power of ESCOTT compared to ESM1b, to highlight, not only differences on the borders of the splicing but also along the shorter form of the protein. This case study was considered previously in the ESM1b article and we could compare to it. The new **Figure 5**  describes the *TP53* analysis.
7. We further analysed the **gain-of-function data** **with iGEMME**, providing new results, and replaced **Figure 8F** by adding iGEMME performance.
8. We added a new panel to **Figure S5**, devoted to AlphaMissense performance on structured regions and coiled regions for the 76 single point mutation DMS experiments in ProteinGym.
9. We introduced a new analysis on the prediction of mutational effects for **disordered regions** by considering the **lpDDT metric**. This analysis, run for ESCOTT and AlphaMissense, supports the one based on secondary structures that was already present in the manuscript. See the new **Figure S6**.
10. We added a new panel (**B**) to **Figure S7** reporting the performance of AlphaMissense on mutational effects towards **hydrophilic vs hydrophobic** amino acids. We show that AlphaMissense behaves as ESCOTT.
11. We performed an analysis of PRESCOTT’s performance on subsets of the 1883 human proteins, defined by varying number of variants per proteins. We added the new **Figure S8**.
12. We performed an analysis on the distribution of PRESCOTT scores, prior to setting negative values to 0. We added a new panel **D** in **Figure S9**.
13. The new **Figure S11** illustrates the analysis of T_JET_, CV and PC the positions associated to gain-of-function mutations.
14. We augmented the sizes of characters in **Figure 8**.
15. **Figure 2F** was improved.

to answer to various comments from the referees, we added new Tables:

16. The new **Table S1** adds new statistical tests on performance comparison.
17. We analysed the case of the unique **genetic heritage of the Finnish population**. For this, we analysed the complete list of 22 missense mutations associated to FDH with ESCOTT/PRESCOTT and AlphaMissense. The new **Table S2** reports the comparison.
18. We also developed two further case studies of **gain-of-function on the *STAT1* and *PTPN11* genes**. We used them to compare to AlphaMissense, show the difficulties of both methods to predict gain-of-function mutations but also other recessive and dominant variants. We added two new tables, **Table S3** describing *STAT1* analysis and **Table S4** dedicated to *PTPN11.*

We supplied several other analyses and among them:

19. We discussed ESCOTT on **different inheritance modes**. A direction which was completely missing before. We added a new paragraph to the text in **pp 12**.
20. The *Discussion* section has significantly evolved to include further points required by the reviewers. It improved on the previous text on a number of directions.
21. A few **new references** have been added to the text:

Uusimaa J, Kettunen J, Varilo T, Järvelä I, Kallijärvi J, Kääriäinen H, et al. The Finnish genetic heritage in 2022–from diagnosis to translational research. Disease Models & Mechanisms. 2022;15(10):dmm049490.

Hijikata A, Tsuji T, Shionyu M, Shirai T. Decoding disease-causing mechanisms of missense mutations from supramolecular structures. Scientific reports. 2017;7(1):8541.

Paul S, Kollasch A, Notin P, Marks D. Combining Structure and Sequence for Superior Fitness Prediction. In: NeurIPS 2023 Generative AI and Biology (GenBio) Workshop. Available from: https://openreview.net/forum?id=8PbTU4exnV

We hope these revisions meet your expectations and address all concerns satisfactorily.

We look forward to your positive decision.

Best wishes,

Alessandra Carbone

GBIO-D-24-00449
PRESCOTT: a population aware, epistatic and structural model accurately predicts missense effect
Mustafa Tekpinar; Laurent David; Thomas Henry; Alessandra Carbone
Genome Biology

Dear Prof. Carbone,

Thank you very much for submitting your manuscript entitled 'PRESCOTT: a population aware, epistatic and structural model accurately predicts missense effect' to Genome Biology. I am extremely sorry that peer review has taken so long. We had some trouble getting reviewers to agree, but I fully appreciate that we should have turned this around much, much faster. Your manuscript has now been seen by two referees and their comments are accessible below. 

As you will see, the reports are broadly favorable, so we are interested in publishing the manuscript, but we feel that the issues raised must be addressed in full, in the form of a revised manuscript, before we make a firm commitment to publication.  In particular, both reviewers ask you explore the conditions under which ESCOTT/PRESCOTT outperform AlphaMissense, particularly in low quality structural regions, and they both query how the method works in bottlenecked populations. Referee 1 asks that you demonstrate the relative performance of ESCOTT and PRESCOTT with different categories of variant or mutation. Referee 2 has raised a minor issue with getting the software to run.  It seems to us to be essential that all of the referees’ concerns are fully addressed, in the form of a revised manuscript, before we can reach a final decision on publication. 


We are considering your manuscript as a 'Method' article. This is our format for publishing articles that describe a methodological innovation that is a significant advance over published methods and likely to be of broad utility, but that do not provide significant biological insights. When revising the manuscript, please ensure the manuscript conforms to our style for Methods articles (see <https://genomebiology.biomedcentral.com/submission-guidelines/preparing-your-manuscript/method>); specifically, the abstract should be under 100 words. Please note that if we decide to publish your manuscript we will require that the source code is made publicly available under an open source license compliant with Open Source Initiative, with the license clearly stated in the manuscript. The source code should be deposited in a public repository, such as for instance github, with the accession links included in the manuscript. We also ask that the version of source code used in the
manuscript is deposited in a DOI-assigning repository, such as zenodo, with the link also included. All this information should be listed in a separate Availability of Data and Materials section of the manuscript.

If you are able to fully address these points, we would encourage you to submit a revised manuscript to Genome Biology.  Once you have made the necessary corrections, please submit online at:
<https://www.editorialmanager.com/gbio/>

Your username is: [alessandra.carbone@lip6.fr](mailto:alessandra.carbone@lip6.fr)
<https://www.editorialmanager.com/gbio/l.asp?i=637211&l=14XY13M2>

Please include a cover letter with a point-by-point response to the comments, describing any additional experiments that were carried out and including a detailed rebuttal of any criticisms or requested revisions that you disagreed with. Please also ensure that all changes to the manuscript are indicated in the text by highlighting or using track changes.

We hope to receive your revised manuscript within the next six weeks or so. Please let us know if the delay is likely to be longer than six weeks, or if you have any problems or questions.

I look forward to receiving your revised manuscript.

Best wishes,

Andrew

Andrew Cosgrove, PhD
Genome Biology
<https://genomebiology.biomedcentral.com/>

Reviewer reports:

Reviewer #1: In this article, the authors proposed a new computational approach, called PRESCOTT, to predict the effects of missense variants. PRESCOTT integrates three major biological information: evolutionary conservation, protein structural importance, and population allele frequency. The authors demonstrated the superior performance of PRESCOTT (and its precursor ESCOTT) to other metrics in predicting pathogenic variants using a few mutation datasets. They also built a user-friendly website to facilitate the application of their methods as well as the sharing of precomputed results. While the proposed framework overall appears promising, a series of improvements and/or clarifications need to be made.

We are sincerely grateful for your detailed and insightful review of our work. Your comments provided valuable perspectives that encouraged us to refine our analyses and, we hope, enhance the clarity of the manuscript. We hope these improvements address your observations as intended and further increase your interest in PRESCOTT.

1.   Based on the Methods section, the TJET value comes from iGEMME, but it is not clear 1) in the Main text that TJET is a metric developed previously

T_JET_ has been developed back in 2009, in a context of prediction of protein binding sites for a protein with no knowledge of its partners. This has been stated explicitly in the text now, **pp3**:

“Given the diverse evolutionary and physico-chemical characteristics of protein binding sites, previous studies (23,24) have shown that combining PC with evolutionary conservation provides a more accurate model for identifying critical residues at interfaces. The concept of evolutionary conservation, called T_JET_ and introduced in (23), is utilized by ESCOTT and illustrated in **Figure 1C**.”

and 2) what improvement iGEMME has been made over GEMME.

The improvement of iGEMME over GEMME is due to a new parameterisation of GEMME. This was already explained in the *Methods* section “The ESCOTT program is built on iGEMME”, **pp19** (bottom). Now, we listed explicitly the **four parameters** that we changed:

“ESCOTT relies on an improved version iGEMME of GEMME. In iGEMME, the characteristics of the input sequence dataset and default parameters are optimized in four ways compared to the original GEMME program:

1. when a user does not provide an MSA, the default method to obtain MSAs in the GEMME webserver is PSI-BLAST *(84,85)*. In ESCOTT, we use the Colabfold method *(74)* due to its efficiency and clustering procedure.

2. the maximum number of sequences processed has been increased from 20000 to 40000.

3. a parameter that restricts the number of sequences due to computational load (CPU load or max_load parameter) was increased from 500,000 to 8,000,000.

4. the maximum heap size in the calls of the JET program was increased from 1,024MB to 8,192MB.”

It would be helpful to make these explicit to the readers, highlighting which aspects of the proposed method are new and which build on prior work.

ESCOTT/PRESCOTT introduces several conceptually new characteristics compared to the previous GEMME approach. We discussed them **in the *Discussion* section, pp13**:

- *Modelling of residue positions.* In GEMME, amino acid residues in a protein are modeled based solely on evolutionary conservation: the model details the global evolutionary conservation of a residue in the species tree and its local conservation within a phylogenetic clade. In ESCOTT/PRESCOTT, however, residues are modeled using a combination of sequence information (T_JET_) and structural information, describing the position of the residue within a structure (CV) and its physico-chemical properties (PC). This integration of evolutionary conservation with structural and physico-chemical features enables the model to account for essential factors such as whether a residue is buried in the structural core, located at a protein interface, or part of a binding site. These properties are crucial for understanding a mutation's impact on protein stability, dynamics, and function.

- *Redefinition of the epistatic effect of a mutation from its structural context.* The epistatic effect of a mutation in a query sequence q is assessed, in GEMME and other approaches, by measuring the importance of the positions that have mutated relative to q in natural sequences s carrying the mutation. The minimal effect across all natural sequences serve as a proxy. GEMME takes an evolutionary perspective, measuring the importance of a position through evolutionary conservation (T_JET_) and assessing the epistatic effect as a function of the evolutionary conservation of all residues in s that differ from q. In contrast, ESCOTT/PRESCOTT incorporates the structural context in which the mutation occurs, distinguishing between structured and coiled regions, and adjusting the modeling of positional importance accordingly. For coiled region, ESCOTT/PRESCOTT evaluates mutations similarly to GEMME, relying primarily on evolutionary conservation. However, for structured regions, ESCOTT/PRESCOTT integrates evolutionary conservation with structural and physico-chemical features to model a residue’s structural and functional importance with Maxscore. This represents a significant advancement over GEMME's purely evolutionary perspective. By incorporating the structural context, ESCOTT/PRESCOTT improves both the interpretability and predictive power of the model, enabling it to capture subtle mutational effects, such as those mediated through interactions at protein-protein interfaces. These enhancements make the model more applicable to real-world challenges, and opens a new perspective in modeling positional importance within a unified framework that could be further expanded to capture diverse mutational effects.

- *Incorporation of allele frequencies.* Unlike GEMME, PRESCOTT incorporates allele frequency data into its modeling. This addition enables a broader and more nuanced interpretation of mutational effects in human proteins, further enhancing the framework’s predictive and interpretive capabilities.

2.   Among the three components in PRESCOTT, two are mutation-specific (conservation and allele frequency) while the protein structural fractures are at the residue level. What are the authors' perspectives on adding mutation-specific information to CV and PC - for instance, CV(mutatnt)-CV(wild-type)? Would that be technically feasible under the current framework?

Not exactly. Note that:

- T_JET_ and CV are defined at the **positional level**
- PC and allele frequency are defined at the **residue level**.

In particular, “evolutionary conservation” (T_JET_) is **not** defined at the residue level.

The remark is very interesting but we believe that any analysis on this direction should imply a serious study including structural information. Our technical framework does not, as it stands, to immediately include 3D/spatial informations, which probably will be determinant to a satisfactory answer. Note that in ESCOTT, PC and CV are very fast measures to compute. In particular, PC highlights the residues that are expected to be at the interface, since we know, by available statistics, that residues lying at the binding interfaces are more sensitive to mutations. On the other hand, there is no attempt of using predicted interfaces but rather to use PC as a proxy for them. Even if a rough measure, PC positively contributes to ESCOTT predictions.

**In the discussion, pp14, we added a paragraph in this direction**: “Among PRESCOTT’s four core amino acid measures—T_JET_, CV, PC, and allele frequency—T_JET_ and CV are defined at the positional level, while PC and allele frequency are defined at the residue level. Expanding the model to include mutation-dependent descriptions of T_JET_ and CV could unlock exciting possibilities, likely requiring 3D representations of residues. This enhancement would be particularly impactful in contexts where protein interfaces are explicitly incorporated into the model, enabling the classification of 3D mutational patterns and providing deeper insights into the structural and functional consequences of mutations.”

3.  The authors focused on two protein structural features - the core and the interface. Albeit important, functional regions are not necessarily located at the core or the interface, like certain ligand/small molecule biding pockets. While some of the important  motifs can be captured by TJET, non-core/interface regions would still be deprioritized by CV and PV.

The performance of iGEMME compared to ESCOTT is reported in **Figure S13** on 87 Deep Mutational Scanning experiments. Here, we demonstrated that including PC and CV the results, globally, improve the performance, on essentially all proteins.

Nonetheless, in principle, the referee is right. Pockets, are usually very conserved, so expected to be represented by high T_JET_ scores. If this is the signal that is pursued, the user could run iGEMME instead of ESCOTT. Remember that ESCOTT with no structure (structures are optional in the ESCOTT system) is iGEMME, that is the improved version of GEMME that works on T_JET_ only. We added a remark on this observation within the new paragraph devoted to “**General recommendations for using ESCOTT versus PRESCOTT”, pp12.**

4.  The selection of CV and PV as the sole measurements for each feature could benefit from incorporating additional or more recent metrics. For example, the solvent-accessible surface area (SASA) score can complement CV; and for PV, there are many new approaches predicting protein interaction interfaces - I would highly encourage  the authors to consider incorporating these advancements.

Introducing new knowledge, like protein interfaces or domain-domain interfaces into the system, will be done in the future. It is definitly some direction worth improving. Globally, we believe that the framework introduced in this article will stimulate new models of the “importance of an amino acid” in a protein which could replace our simple and fast to compute MaxScore.

Please see our Discussion, pp13-14.

5.    While this may not be the authors' intension, the introduction on CV and PV referenced their utilities in measuring loss-of-function (pg3 line 1-2,5-6). Are such metrics also able to capture gain-of-function, especially given that the authors later highlighted the strength of their approaches in predicting gain-of-function effects?

This is an intriguing question. Differentiating between loss-of-function and gain-of-function predictions was not our primary intention, in pp3. However, we examined iGEMME's performance on the 128 gain-of-function mutations and found it comparable to ESCOTT and PRESCOTT, whereas AlphaMissense showed a distinct distribution, identifying only 45 mutations as pathogenic.

| **method** | **pathogenic** | **ambiguous** | **benign** |
| --- | --- | --- | --- |
| **iGEMME** | 91 | 14 | 23 |
| **ESCOTT** | 87 | 17 | 24 |
| **PRESCOTT** | 86 | 16 | 26 |
| **AlphaMissense** | 45 | 19 | 64 |

Notably, across these 128 mutations, there is no clear evidence to suggest that CV and PC play a decisive role in predicting gain-of-function effects. We have updated **Figure 8F** to include iGEMME results. We discussed this point in pp12.

We updated the file “gain-of-function-mutations.csv” by including iGEMME scores.

6.  The authors illustrated in Figure 1F "for a position i occupied by residue P in q, the distance within the tree to the closest sequence, say s, displaying R at position i. The intuition is that if s is located far from q in the tree, the P-to-R mutation should require a large number of mutations in q for R to be "accepted" and therefore P-to-R should probably be deleterious". I wander whether there are cases where multiple sequences/subtrees display R, and will that create more chances for R to be "accepted"? Would considering the number of sequences/subtrees and their distances (rather than the min distance) improve the estimation of TJET?

We rewrote the text by adding more information, right from the beginning, about the method. We explained that the notion of tree used in **Figure 1FG** is for explaining the intuition because the computation is done on sets. See **pp.4.**

By reading the referee comment, I think there is a misunderstanding on the usage of trees in T_JET_ calculation and in ESCOTT. The measure “min distance” has been defined for all **pairs** of sequences and was designed to avoid the construction of MSAs/distance trees.

Notice that in our definition **all sequences** are considered in **pairs**, and **all of them** contribute to the min distance measure. The aim here is to find the **closest** sequence s to the query q, that is a sequence s with the minimal change measured by the additive importance of the residues in s changed wrt q.

**Note.** Our calculations of the “min distance” are **not** based on subtrees with sampled sequences. This was done in T_JET_  and the referee might have been confused on this point. Indeed, the purpose here is not to “forget” sequences and avoid errors (this is the main aim of using the MSA sampling in JET) but rather to consider all possible sequences to find the one that underlies the minimal constraints in changing the amino acid P into R.

7.   PRESCOTT selects the popmax allele frequency based on the assumption that "if a variant is common in one population, it can be assumed to be benign across all populations". However, this assumption may not hold well -  for instance, the Finnish population has a unique genetic heritage due to its historical isolation and population bottlenecks, which has led to the enrichment of certain pathogenic variants, known as the Finnish Disease Heritage (FDH). FDH comprises nearly 40 rare monogenic diseases that are over-represented in Finland but rare elsewhere [PMID: 36285626]. Relatedly, how often / what are the cases where ESCOTT outperforms PRESCOTT?

gnomAD database reports allele frequencies from individuals which are healthy and free from significant diseases. Strictly speaking, this is largely accurate, as described in:

Gudmundsson, Sanna, et al. "Variant interpretation using population databases: Lessons from gnomAD." *Human Mutation* 43.8 (2022): 1012–1030.

where the authors also explain: *"While we expect individuals with severe early‐onset disease to be heavily depleted from gnomAD, individuals with conditions that would not prohibit participation in common disease research are likely included at mildly depleted or similar levels to the general population, particularly for phenotypes like infertility, vision and hearing impairment, and conditions with late onset or reduced penetrance."*

In the Method section, we added a sentence to this end:

“It is important to note that this principle is idealized, as gnomAD heavily depletes data from individuals with severe early-onset diseases. However, it only mildly depletes or even includes data from individuals with phenotypes such as infertility, vision and hearing impairment, or conditions with late onset or reduced penetrance (32). As a result, a doctor's understanding of potential biases—such as the Finnish case discussed in the manuscript or similar situations—can guide the decision to favor ESCOTT over PRESCOTT when appropriate.”

This point has been clarified also in a **new section** “ESCOTT and PRESCOTT on unique genetic heritage”, pp 11, where **a new analysis** of pathogenic variants, collectively known as the Finnish Disease Heritage (FDH), has been conducted with ESCOTT, PRESCOTT and AlphaMissense. We provide the following **new Table S1**:

| **Gene** | **Missense mutation** | **Mutation acronyme** | **UniprotID** | **ESCOTT score (>0.42)** | **PRESCOTT** | **log10frequency**  **in gnomAD** | **Selected Population** | **AlphaMissense** |
| --- | --- | --- | --- | --- | --- | --- | --- | --- |
| **AGA** | Cys163Ser | C163S | P20933 | 0.55 | 0.08 | -2.12 | European (Finnish) | 0.958 |
| **PPT1** | Arg122Trp | R122W | P50897 | 0.87 | 0.41 | -2.16 | European (Finnish) | 0.739 |
| **KERA** | Asn247Ser | N247S | O60938 | 0.82 | 0.41 | -2.37 | European (Finnish) | 0.882 |
| **CRADD** | Arg170His | R170H | P78560 | 0.21 | 0.00 | -2.23 | European (Finnish) | 0.082 |
| **CLN8** | Arg24Gly | R24G | Q9UBY8 | 0.91 | 0.65 | -2.97 | European (Finnish) | 0.615 |
| **GSN** | Asp214Asn | D214N | P06396 | 0.60 | 0.60 | No frequency |  | 0.719 |
| **GLDC** | Ser564Ile | S564I | P23378 | 0.84 | 0.52 | -2.73 | European (Finnish) | 0.960 |
| **BCS1L** | Ser78Gly | S78G | Q9Y276 | 0.48 | 0.08 | -2.41 | European (Finnish) | 0.253 |
| **HYLS1** | Asp211Gly | D211G | Q96M11 | 0.96 | 0.47 | -2.05 | European (Finnish) | 0.973 |
| **OAT** | Leu402Pro | L402P | P04181 | 0.94 | 0.59 | -2.59 | European (Finnish) | 0.995 |
| **TWNK** | Tyr508Cys | Y508C | Q96RR1 | 0.68 | 0.32 | -2.58 | European (Finnish) | 0.191 |
| **FSHR** | Ala189Val | A189V | P23945 | 0.62 | 0.16 | -2.17 | European (Finnish) | 0.386 |
| **PCK1** | Gly309Arg | G309R | P35558 | 0.94 | 0.43 | -1.95 | European (Finnish) | 0.997 |
| **ZNHIT3** | Ser31Leu | S31L | Q15649 | 0.96 | 0.55 | -2.36 | European (Finnish) | 0.743 |
| **RS1** | Glu72Lys | E72K | O15537 | 0.64 | 0.64 | -4.37 | European (Finnish) | 0.918 |
| **SLC17A5** | Arg39Cys | R39C | Q9NRA2 | 0.93 | 0.52 | -2.34 | European (Finnish) | 0.968 |
| **CHCHD10** | Gly66Val | G66V | Q8WYQ3 | 0.52 | 0.52 | -4.32 | European (Finnish) | 0.960 |
| **TK2** | Arg183Trp | R183W | O00142 | 0.74 | 0.51 | -3.09 | European (Finnish) | 0.293 |
| **AICDA** | Met139Thr | M139T | Q9GZX7 | 0.92 | 0.60 | -2.73 | European (Finnish) | 0.987 |
| **HADHA** | Glu510Gln | E510Q | P40939 | 0.73 | 0.32 | -2.36 | European (Finnish) | 0.986 |
| **POLG** | Trp748Ser | W748S | P54098 | 0.45 | 0.00 | -2.20 | European (Finnish) | 0.766 |
| **CUBN** | Pro1297Leu | P1297L | O60494 | 0.77 | 0.39 | -2.50 | European (Finnish) | Not computed |

ESCOTT systematically identifies pathogenic mutations across all cases except one (in the *CRADD* gene). In contrast, AlphaMissense misclassifies five pathogenic missense mutations as benign (*CRADD, BCS1L, TWNK, HADHA,* and *POLG*), one as VUS (*FSHR*), and does not provide a score for the *CUBN*gene, likely due to the protein’s length. When allele frequency specific to the Finnish population is incorporated into PRESCOTT, we observe significant adjustments to the scores, leading to erroneous evaluations in 10 cases, as pointed out by the referee. Based on these findings, **we recommend prioritizing ESCOTT scores over PRESCOTT** and refraining from population-specific allele frequency adjustments in contexts of unique genetic heritage, such as the Finnish population.

8.   It is exciting that the proposed approach can be applied to a diverse range of "large proteins, intrinsically disordered regions, splicing variants, mutated sequences, and artificially generated sequences". Could the authors provide some insights into the relative performance (like AUCs) of PRESCOTT/ESCOTT within each of these categories? This would help illustrate the strengths and limitations of the approach across diverse use cases.

To provide such a thorough analyses with large scale tests would imply a huge amount of work due to the extensive data available. We decided to focus on three analyses that remain feasible and highlight the interests on the approach:

“It can be applied to large proteins, intrinsically disordered regions, and splicing variants.”

The sentence has been changed accordingly in **pp6**.

**Analysis of disordered vs ordered regions using the pLDDT measure**. The performance of ESCOTT and AlphaMissense was evaluated on the DMS matrices for predictions restricted to regions with pLDDT >.7, a proxy for ordered regions, and <.7, a proxy for disordered regions. Two new figures, included as **Figure S6,** demonstrate a significant drop in performance for both ESCOTT and AlphaMissense when applied to these regions.

This new analysis of regions with varying pLDDT values complements the original analysis of coiled regions vs regions with secondary structures, as presented in the first version of our manuscript (**Figure S5**). Additionally, we **have updated Figure S5** to include AlphaMissense’s performance on regions defined by secondary structures, which was missing in the initial version.

The new analysis is reported in **pp.8.**

**Analysis of long proteins**. Our citation of short vs. long proteins concerns a limitation of some existing predictors, such as the EVE approach, which cannot fully process long proteins or handle them effectively. In contrast, ESCOTT does not face this issue. For the human proteome, we successfully generated predictions for all proteins, including very long ones exceeding several thousand amino acids. These can be analyzed using the server or, more efficiently, with the Docker image, which includes all necessary dependencies. In general, we recommend using the Docker image for scenarios involving:

- Very long reference sequences (>3,500 residues),
- Poor representation of the reference sequence within the MSA,
- Positions with many gaps,
- Insufficient sequences in the MSA, or
- Overly long sequence names.

This recommendation has been added to the manuscript, **pp 22-23.**

**Analysis of isoforms**. We showed that ESCOTT/PRESCOTT can answer positively to the following question: Can it help predict the impact of variants classified as VUS in ClinVar across different isoforms? We reconsidered this challenge proposed in

Brandes, Nadav, et al. "Genome-wide prediction of disease variant effects with a deep protein language model." *Nature Genetics* 55.9 (2023): 1512-1522.

for ESM1b, by performing an extensive analysis of two isoforms for the P53 protein. We compared the primary isoform of P53 with isoform 8. For ESM1b, the authors mention that most differences are identified by ESM1b on the “borders” of the spliced variant and showed the comparison between isoforms in Figure 4B of their article. With ESCOTT, we analysed all the landscape of single mutations, including all missense mutations present in ClinVar. Our findings align closely with the signals identified by ESM1b at the “borders” of the spliced variant (see Figure 4 in (18)). Unlike ESM1b, ESCOTT identifies also many score differences throughout the full length of isoform-8, not just at its borders. A comprehensive list of relevant variants for the isoform-8 is provided in the supplemental excel file, which includes ESCOTT scores and ClinVar labels (pathogenic, likely pathogenic, benign, likely benign, or VUS).

This analysis is reported in a **new paragraph**, pp 10, and in the new **Figure 5C**.

In this analysis, human variants are classified as Benign, Pathogenic, or VUS by ESCOTT using the thresholds derived from PRESCOTT. To this end, we added a **new paragraph** in the *Methods* section (**pp. 22**): “Classification of Human Variants as Benign, Pathogenic, and VUS by iGEMME and ESCOTT using PRESCOTT thresholds”

9.    Similarly, the authors tested the performance of ESCOTT/PRESCOTT on 32 human protein DMS experiments and its generality across species on all the 87 DMS experiments. Does ESCOTT/PRESCOTT perform better in human proteins vs other species, or any specific classes of proteins? For example, the authors already observed variable performance across different proteins - with Spearman correlation coefficients ranging from 0.135 for SCN5A to 0.690 for P53 - understanding the underlying reasons would be important for the reader to better interpret the approach as well as the results.

Here is the table on different species:

| **Species** | **ESCOTT** | **AlphaMissense** |
| --- | --- | --- |
| **Human** | 0.460 | 0.460 |
| **Viruses** | 0.474 | 0.540 |
| **Prokaryotes** | 0.553 | 0.584 |
| **Other Eukaryotes** | 0.503 | 0.564 |
| **All** | 0.492 | 0.525 |

These values are now reported in **a new sentence**, pp. 7.

We agree with the reviewer about the importance of understanding the data. The real challenge in variant prediction lies in the complexity of the data. While performance variations between methods are relatively minor, success consistently depends on the specific set of proteins/experiments being analyzed—some proteins/experiments inherently pose greater difficulties. Future developments must focus on addressing the complexities inherent in disordered regions, and diverse experimental categories [Paul et al. NeurIPS 2023], where current methods, including ESCOTT, face significant limitations. We added **a new paragraph** in the *Discussion* to this end.

10.  In the comparison of performance, the authors used average values of Spearman correlation coefficient. It would be useful to supply a measurement of the significance of improvement. For instance, the authors could consider using a paired u-test to evaluate the differences shown in Figure 3A, 5A, etc.

We applied the Mann-Whitney paired u-test, ranking Spearman correlation coefficients, and report the p-values in SI. The outcomes can be coupled with the Spearman correlation coefficients that we used in the paper. Here are the results of the paired u-test for **Figure 3A**:

| **Method 1** | **Method 2** | **p-value** | **statistic** |
| --- | --- | --- | --- |
| **ESCOTT** | EVE_ensemble | 0.0002 | 55.0000 |
| **ESCOTT** | ESM1b | 0.0008 | 92.0000 |
| **ESCOTT** | AlphaMissense | 0.5483 | 231.5000 |
| **EVE_ensemble** | ESM1b | 0.5119 | 227.5000 |
| **EVE_ensemble** | AlphaMissense | 0.0029 | 96.0000 |
| **ESM1b** | AlphaMissense | 0.0001 | 67.0000 |

where we observe that our conclusions are coherent with the paired u-test:

- **ESCOTT consistently outperforms both EVE_ensemble and ESM1b**, showing statistically significant differences, which emphasizes its superior predictive capabilities in these comparisons. Also, **AlphaMissense stands out as significantly better than EVE_ensemble and ESM1b,** confirming its reliability in comparison to these methods.
- **AlphaMissense matches ESCOTT’s performance,** as no significant difference is observed between the two, suggesting they are both strong performers.
- **EVE_ensemble and ESM1b show no significant differences in performance,** indicating they are similarly positioned in this evaluation.

ESCOTT emerges as a robust method, with significant performance advantages over EVE_ensemble and ESM1b, while maintaining parity with AlphaMissense. This highlights its strong overall behavior and reliability across datasets, comparable only to AlphaMissense, which also consistently outperforms the other approaches.

For **Figure 6A** (previously Figure 5A):

| **Method 1** | **Method 2** | **p-value** | **statistic** |
| --- | --- | --- | --- |
| **ESCOTT Averaged** | EVE (Ensemble) Averaged | 0.0024 | 74.0000 |
| **ESCOTT Averaged** | ESM1b Averaged | 0.0089 | 89.5000 |
| **ESCOTT Averaged** | AlphaMissense Averaged | 0.0628 | 120.5000 |
| **EVE (Ensemble) Averaged** | ESM1b Averaged | 0.9019 | 196.5000 |
| **EVE (Ensemble) Averaged** | AlphaMissense Averaged | 0.1502 | 139.0000 |
| **ESM1b Averaged** | AlphaMissense Averaged | 0.0735 | 124.0000 |

- **ESCOTT stands out** as performing significantly better than both EVE (Ensemble) and ESM1b in this analysis.
- **ESCOTT and AlphaMissense perform comparably,** with no significant difference observed between them.
- **EVE (Ensemble), ESM1b, and AlphaMissense** show no statistically significant differences in performance among themselves.

**ESCOTT demonstrates a better overall performance**, as it shows statistically significant differences compared to EVE (Ensemble) and ESM1b, highlighting its robustness, while its comparable performance with AlphaMissense suggests it maintains competitive effectiveness even against strong alternatives.

For **Figure 7C**:

| **Method 1** | **Method 2** | **p-value** | **statistic** |
| --- | --- | --- | --- |
| **PRESCOTT** | AlphaMissense | 0.0016 | 164.0000 |
| **PRESCOTT** | ESM1b | 0.0000 | 33.0000 |
| **PRESCOTT** | EVE | 0.0000 | 41.5000 |
| **PRESCOTT** | ESCOTT | 0.0000 | 5.5000 |
| **AlphaMissense** | ESM1b | 0.0000 | 74.0000 |
| **AlphaMissense** | EVE | 0.0000 | 92.0000 |
| **AlphaMissense** | ESCOTT | 0.0007 | 181.0000 |
| **ESM1b** | EVE | 0.1124 | 308.0000 |
| **ESM1b** | ESCOTT | 0.0005 | 173.0000 |
| **EVE** | ESCOTT | 0.0853 | 282.0000 |

PRESCOTT emerges as the strongest method, consistently showing statistically significant advantages over other approaches. AlphaMissense also demonstrates strong performance, particularly when compared to ESM1b and EVE. ESCOTT holds its ground against EVE but is clearly outperformed by PRESCOTT. This table highlights the robustness of PRESCOTT and AlphaMissense in this analysis.

For each figure we added the reference to the **new analysis** in the main text. The three tables above have been included as a supplementary material as **Table S1**.

11.   It is interesting that some regions not well modeled by AlphaFold are recognized by both ESCOTT and AlphaMissense. I wonder how low-quality structural regions are handled when computing structural features (CV and PV)?

The ESCOTT/PRESCOTT model differentiates between coiled regions (longer than 5 amino acids), considered a proxy for disorder, and regions with defined **secondary structures**. Notably, we did not explicitly use **AlphaFold’s lpDDT** **metric** to define disordered regions. In the ESCOTT model, coiled regions longer than 5aa are handled exclusively with T_JET_, whereas regions with secondary structures are handled using a combination of T_JET,_ CV and PC. This distinction is now more thoroughly explained in the Methods section, with an explicit definition of MaxScore notion, **pp3**.

**In the original manuscript**, we illustrated ESCOTT’s and AlphaMissense’s ability to extract meaningful signals in disordered regions using the **SPASTIN protein** as a case study, where both methods identified sensitive regions within disordered areas (**Figure 4**). **To expand on this,** we have **now added a second example focusing on the P53 protein**, accompanied by a new **Figure 5**. In this analysis, we examined three disordered regions annotated by InterPro, aswell as larger regions with low pLDDT scores predicted by AlphaFold. Both methods consistently highlighted the same regions as potentially sensitive to mutations (indicated in light tones in **Figure 5AB**). These regions include five motifs previously identified in the literature and annotated in InterPro: TAD I, TAD II, the bipartite nuclear localisation signal, the nuclear export signal, the [KR]-[STA]-K motif. Both methods successfully capture all these motifs. **This expanded analysis is detailed** **in pp7**.

12.   As the author stated about Table 1, "AlphaMissense outperforms all methods. Yet it is important to note that its advantage partly stems from being explicitly finetuned to distinguish between pathogenic and benign effects. In contrast, ESCOTT, along with the other comparative methods, does not utilize classified data in its modeling process". Is there a particular reason for the many other approaches avoid training on pathogenic vs benign effects? If so, it would be critical to elaborate on scenarios where such a strategy is superior to AlphaMissense (which may fail to recognize certain pathogenic features that were not included in its training data?)

AlphaMissense generates predictions based on allele frequencies, sequences and structural information. On human proteins, ESCOTT achieves comparable performance while relying solely on sequence and structural data. In ESCOTT, allele frequency is used as an additional layer to enhance predictions, and our results confirm its utility.

Although AlphaMissense is trained on allele frequency data and could theoretically predict some positions more effectively due to its training, our analysis (**Figure 7A** and **Figure 7C**) does not support this hypothesis.

We previously overlooked mentioning that the PRESCOTT model can be coupled can be coupled to any predictor as a postprocessing step, enabling all methods to potentially improve their predictions for human missense variants. This important observation has now been emphasised in the *Discussion* section. Note that we had shown the improvement on EVE though.

13.   The authors showed that ESCOTT outperforms AlphaMissense in predicting "sensitive regions", which is encouraging. But this seems to hold true only for the 28 DMS (Figure 5A) but not all DMS, as shown in Figure S3A? It may be worth clarifying in the main text.

We added a sentence to this end in **pp 8.**

14.    The section "Some global observations lead to an improved interpretation of ESCOTT scores" provides important information about the properties of the proposed approach. I would suggest 1) include AlphaMissense in the comparisons shown in Figure S4 and S5 and 2) elaborating in the Discussion on possible underlying reasons for these properties, e.g., why it predicts mutations toward hydrophilic residues (D, R, E, K, N, Q) particularly well?

We have addressed the referee's suggestions as follows:

1. We incorporated AlphaMissense analyses into both **Figure S5** (Formerly **Figure S4**) and **Figure S7B** (formerly **Figure S5**). Additionally, we included AlphaMissense in the new analysis described in **Figure S6**.
2. In the *Discussion*, we expanded on the observation regarding mutations toward hydrophilic versus hydrophobic amino acids. Interestingly, existing predictors, including AlphaMissense, EVE, DeepSequence, ESM2, MSA-Transformer, and others (not shown), exhibit a similar pattern: they perform better on mutations toward hydrophilic amino acids compared to hydrophobic ones. This general trend across predictors has now been explicitly noted in the *Discussion*. We also propose new directions for investigation, such as analyzing this observation across the five classes of DMS experiments, “Expression”, “Binding”, “Organismal Fitness”, “Activity” and “Stability” [Paul et al 2023, NeurIPS 2023] which may exhibit variations in amino acid distribution. Exploring this observation further could be important for advancing and refining predictive methods.

15.  It is impressive that PRESCOTT achieved a high AUC on both the ACMG proteins and the 1,883 gnomAD proteins. I wonder how sensitive PRESCOTT's performance is, compared to other methods, to the sample size (i.e., the number of variants evaluated per protein)? 

Here is the analysis:

We inserted it as **Figure S8** and discussed **the new analysis** **in pp.9**.

16.   The ability of ESCOTT/PRESCOTT to identify gain-of-function is particularly exciting. But I assume that the final score does not distinguish between loss- and gain-of-function (?) It would be interesting to explore whether some intermediate scores could indicate loss- vs gain-of-function effects.

This is correct. The final score does not distinguish between what is gain and what is loss-of-function. This question is very interesting, and, before the first submission, we did explore many features for distinguishing the two classes of mutational effects but, unfortunately, we could not come up with specific determinants. Note that the observation on the localisation of gain-of-function mutations highlighted in [Hijikata et al., Scientific Report, 2017] which have been found enriched in domain-domain interfaces, might be a good starting point to discriminate the two classes of effects.

A sentence has been added in the *Discussion* section and in pp 10.

Since iGEMME demonstrated slightly better performance than ESCOTT on gain-of-function mutations (see new plots in Figure 8F), we investigated the associated T_JET_, PC, and CV distributions for the corresponding 128 positions. As expected, the distributions revealed that gain-of-function mutations tend to occur at highly conserved positions (see plots below), aligning with their localization at domain-domain interfaces, as previously reported by Hijikata et al. (*Scientific Reports*, 2017). We reported this analysis in **pp 12** and in **the new** **Figure S11,** here below:

17.  For the gain-of-function evaluation, the authors created a new dataset of gain-of-function mutations, integrating several data sources including "unpublished experimental data". For greater transparency, I would suggest performing a separate analysis using only published evidence.

Our analysis of gain-of-function mutations was extended to two additional human proteins, STAT1 and PTPN11, as discussed in

Hijikata, Atsushi, et al. "Decoding disease-causing mechanisms of missense mutations from supramolecular structures." *Scientific reports* 7.1 (2017): 8541.

Detailed results are presented in **the new** **Tables S3** and **S4**. STAT1 and PTPN11 include 17 and 45 previously identified GoF variants, respectively. Both case studies highlight the challenge faced by ESCOTT/PRESCOTT and AlphaMissense in classifying GoF mutations. Systematic large-scale analyses are needed to improve classification by mode.

These case studies, along with other variants (23 for STAT1 and 58 PTPN11, including GoF) are discussed on **pp.11,** in the section “ESCOTT, PRESCOTT, iGEMME and AlphaMissense on recessive and dominant inheritance modes”.

18.   In the section "Classification of Human Variants as Benign, Pathogenic …", more details and statistics are expected to describe how categories shift under PRESCOTT compared to ESCOTT. Additionally, it would be desirable to show that variants reclassified from benign to pathogenic are biologically significant - e.g., are enriched for certain functional annotations.

There appears to be a misunderstanding. No variants are reclassified from benign to pathogenic using PRESCOTT. In fact, all variants that have their scores adjusted by PRESCOTT can only see a **decrease** in their score due to the inclusion of allele frequency data from gnomAD. Specifically:

1. Variants classified as pathogenic by ESCOTT may be reclassified as benign or VUS when analyzed with PRESCOTT.
2. A variant can only be classified as pathogenic by ESCOTT.

These **two points** have now been **explicitly clarified** in **pp.10.**

19.  In the same section, the authors stated that "ESCOTT's accurate predictions for pathogenic variants are achieved without relying on allele". This could be confusing, given that that PRESCOTT is designed to improve ESCOTT in classifying pathogenic vs. benign variants. It would be helpful to provide insights into how one should choose between ESCOTT and PRESCOTT when their predicted categories differ.

We understand the concern. Thanks for pointing it out.

As a global strategy, one should always use PRESCOTT whenever allele frequency data is available, to improve on the prediction of benign variants. As discussed in the new text now, when allele frequency comes from populations with unique genetic heritage, we suggest the user not to use PRESCOTT but prefer ESCOTT instead. From our analysis of very few examples of pathogenic gain-of-function variants, we observed that they are best predicted by iGEMME, by excluding structural information, hence, one should preferentially use iGEMME.

This **raccomendation is now explicitly written** in **pp.12-13.**

20.    The authors found that the analysis of pathogenic mutations in the MEFV protein is particularly challenging for both ESCOTT/PRESCOTT and AlphaMissense systems. Could the author discuss potential reasons and ways of improvement in Discussion?

The analysis of the MEFV gene is particularly challenging for ESCOTT/PRESCOTT and AlphaMissense. For this protein, the oligomeric state may significantly impact predictions and help explain associated challenges. Like most DMS (Deep Mutational Scanning) predictors, ESCOTT/PRESCOTT and AlphaMissense rely on single-protein analyses and do not account for protein-protein interactions. Among existing methods, only MuLAN (Lombardi and Carbone 2024, BioRxiv) incorporates these interactions, allowing for analyses that go beyond the monomeric state. Using MuLAN—a deep learning model designed to predict changes in binding affinities within complexes—we can reconstruct MEFV's mutational landscapes with notable improvements over ESCOTT and AlphaMissense, particularly for pathogenic mutations linked to diseases. Incorporating protein interaction data into mutational landscape reconstructions could further enhance prediction accuracy and it appears as a promising direction in the field. Another critical aspect to improve MEFV predictions is its evolutionary context. The MEFV protein and its homologs likely co-evolved with virulence factors from pathogen-adapted species, leaving distinct evolutionary signals in the human sequence or a subset of homologs. Disentangling these signals requires identifying homologs, pinpointing residues under strong positive selection, and developing tailored strategies to address these co-evolutionary patterns. See **pp.15**.

21.  In the Methods section, the selection of certain thresholds could benefit from further justification or explanation: 1) N=500 proteins for calibrating default parameter values in ESCOTT;

We wished to select a relatively small number of proteins to learn from and maintain a much larger number of proteins for validation. The total number of these proteins is limited and 500 seemed a good compromise. We repeated the random selection several times, with N=500, obtaining the same parameters.

2) α=0.6 as the linear coefficient in the ESCOTT model;

The section “Analysis of the independent and epistatic terms in ESCOTT” is now moved from the methods to the Results and inserted just after the description of the ESCOTT model. It explains to the reader why 0.6 has been chosen, supported by the analysis on **Figure S1**. See **pp.4 and 5**.

additionally, it is not very clear how the relative frequencies of Yi is defined.

As for the relative frequency of Yi, we have added the formula in the text now**, pp 3.**

22.   Given the frequencyCutoff=-4, the minimum sample size to reach a low frequency of 0.0001 is 10,000 individuals. Therefore, it would be more logical to consider only populations with more than 10,000 individuals. For example, in the Middle Eastern population with N=3031, the occurrence of just one mutant allele would result in a "high" frequency and make it downweighed for pathogenicity. However, such variants are still likely to be deleterious (especially if it remains low frequency in other larger populations). I would thus suggest excluding the Middle Eastern population from the analysis avoid potential bias in the results.

The best parameters for the model that we obtain by excluding the middle eastern population remain unchanged, both for the training and for the testing datasets as reported in detail below. The dataset of 2,383 proteins considered in the article, randomly divided in two subsets of 500 and 1,883 proteins, was used for training and testing under this new condition:

Training on 500 proteins:

**With** Middle Eastern data

The best AUC = 0.95

The best scaling coefficient for log10 allele frequency = 1.00

The best cutoff for allele frequency = -4.0

**Without** Middle Eastern data

The best AUC = 0.95

The best scaling coefficient for log10 allele frequency = 1.00

The best cutoff for allele frequency = -4.0

Testing on 1883 proteins:

**With** Middle Eastern data

The best AUC = 0.95

The best scaling coefficient for log10 allele frequency = 1.00

The best cutoff for allele frequency = -4.0

**Without** Middle Eastern data

The best AUC = 0.95

The best scaling coefficient for log10 allele frequency = 1.00

The best cutoff for allele frequency = -4.0

and here, we report the corresponding plots, to be compared with the ones in **Figure 7AB** in the article:

Since there was no contribution of the Middle Eastern dataset, we prefer not to change the code by eliminating the Middle Eastern populations. Instead, we prefer to leave the code as it is, keeping in mind that, in a near futur, the number of allele frequencies coming from these populations will augment and the user will be able to profitably use our software also benefitting from such statistics, today poorly represented.

A sentence to explain this choice has been added, **pp 20-21.**

To augment the user visibility on the procedure followed by PRESCOTT, now, **the PRESCOTT output file (prescott-details.csv file)** contains information on the population allele frequency (‘Selected Population’ column) used in the calculation. The code is updated as well as the docker image.

23.  While the fixation of PRESCOTT values at a min of 0 makes it easier for score interpretation, it would be worth illustrating to the readers how often or to what degree the scores could fall below 0 (in relation to allele frequency if this restriction were not applied). 

We considered the set of 1,883 human proteins used in the article with available gnomAD data. We illustrated the distribution of raw scores, possibly negative on those mutations that have been affected in the new **Figure S9D**. For each starred subset, we also analyzed the distribution of PRESCOTT scores, prior to setting negative values to 0. **Figure S9D** presents this analysis, focusing exclusively on mutations whose scores were adjusted by allele frequency in PRESCOTT. Notably, more than half of the mutations in each subset are affected, with approximately one-third of these adjusted to scores within the range [-1, 0], underscoring the significant impact of allele frequency data on variant classification. This trend becomes even more pronounced when applying the threshold for predicting benign mutations.

We added a sentence in **pp.9**.

24.  The two sub-models of PRESCOTT - PRESCOTTscore-high-allele-frequencies and PRESCOTTscore-all-allele-frequencies - may provide complementary information. It would be valuable to perform similar evaluation tests to compare the performance/different utilities  of these two models.

We apologize for the misleading labels in the legend of **Figure S11**, which we correctly labelled now. Indeed, the two algorithms analysed in the figure are “PRESCOTTscore-high-allele-frequencies” and “PRESCOTTscore-all-allele-frequencies”. Their performance was discussed in the text together with the reasons for not using it. Note that we implemented both algorithms in PRESCOTT code and the user will be able to use PRESCOTTscore-all-allele-frequencies when sufficient data will become available. The code is also available for testing and potential modifications.

Perhaps relatedly, the authors mentioned that "The frequency of gnomAD alleles in the Star >0 set, considering all populations at once, was tested, but performance was slightly inferior (0.94)". But it is unclear what specific performance metric was being measured in this context.

We used the same performance method. We rewrote the sentence in the text by adding extra information now, pp 21:

“The frequency of gnomAD alleles in the Star >0 set, considering all populations at once, was tested, but performance was slightly inferior, with an AUC of 0.94 obtained on the testing set of 1883 human proteins in gnomAD (compare to **Figure 7A**).”

25.  The section "Analysis of the independent and epistatic modules building ESCOTT" in the Methods is interesting - It would be worth elaborating on and moving it to the main text.

We moved it in the main text. We agree that it is better placed and improves the reading. Thank you.

Reviewer #2: Tekpinar, et al. introduces a novel method for predicting the effects of missense mutations, elegantly wrapped in three resources: the ESCOTT web server, ESCOTT Database and PRESCOTT web server. Their method integrates evolutionary conservation, molecular structures, and population variant frequency. Doing so it surpasses existing state-of-the-art computational approaches, including AlphaMissense and ESM1b in a comprehensive comparison evaluated on DMS experiments (e.g. Proteingym). Throughout the manuscript it quickly becomes clear that the largest similarities lie between PRESCOTT and AlphaMissense. Tekpinar et al. can surpass predictive metrics compared to AlphaMissense (although by a slim margin) likely due to the author's different way of incorporating evolutionary conservation and in the way they incorporate population frequency of variants. There are two central novelties in Tekpinar et al.'s approach: Firstly they provide a understandable non-black box type of approach as to why a variant is marked as benign-vus-pathogenic. Secondly, they outperform all other state-of-the-art methods by a small margin.

I would like the congratulate the authors with the tremendous amount of work that is contained within the manuscript and the extra effort to make it usable for the clinical and research community through their webserver. Additionally, I am very happy the authors thought of reproducibility by using publicly available containerization, code and data repositories. I do have the following unclarities that I hope the authors can address followed by a few minor comments.

We sincerely thank you for your thoughtful and encouraging feedback on our work. Your remarks were particularly stimulating, prompting additional analyses that have not only enhanced our results but also improved the clarity of the presentation. We hope these revisions have further increased your interest in PRESCOTT.

Impact of structure and absence of structure on PRESCOTT predictions. Fig 4 shows a strong correlation that when structural confidence (by AlphaFold's pLDDT) goes down, so do the pathogenic predictions for all compared models here.

As mentioned above in answering to the first referee, in this version of the manuscript, we analysed disordered vs ordered regions **using the pLDDT metric**. Before, in our manuscript, we used secondary structure predictions and colied regions as proxy for disorder.

The performance of ESCOTT and AlphaMissense was evaluated on the DMS matrices for predictions restricted to regions with pLDDT >.7, a proxy for ordered regions, and <.7, a proxy for disordered regions. Two new figures, included as **Figure S6,** demonstrate **a significant drop in performance for both ESCOTT and AlphaMissense** when applied to these regions.

This new analysis of regions with varying pLDDT values complements the original analysis of coiled regions vs regions with secondary structures, as presented in the first version of our manuscript (**Figure S5**). Additionally, we **have updated Figure S5** to include AlphaMissense’s performance on regions defined by secondary structures, which was missing in the initial version.

The **new analysis is reported** in pp.8.

However, AlphaMissense seems to still predict pathogenicity in these areas, the yellow dots, which do not correspond to ESCOTT or the other models.

We believe this is an impression due to the yellow-green-blue color scale.

To answer to this remark, we included a new analysis of the P53 protein, accompanied by **the new** **Figure 5,** where we examined three disordered regions annotated by InterPro, as well as larger regions with low pLDDT scores predicted by AlphaFold. **Both methods consistently highlighted the same regions as potentially sensitive to mutations** (indicated in light tones in **Figure 5AB**). These regions include five motifs previously identified in the literature and annotated in InterPro: TAD I, TAD II, the bipartite nuclear localisation signal, the nuclear export signal, the [KR]-[STA]-K motif. Both methods successfully capture all these motifs. This is in agreement with the considerations on the SPASTIN protein already present in the original manuscript. **This expanded analysis** is detailed in **pp6.**

I am curious how ESCOTT/PRESCOTT improves on AlphaMissense in the regions without high confidence structures versus the regions with. This comparison would be more informative then just an overal comparison as provided in the manuscript as this would show in what situation PRESCOTT/ESCOTT improves beyond AlphaMissense. In addition, the manuscript could use a better description on how the authors exactly incorporate the pLDDT confidence values of predicted protein structures.

In ESCOTT/PRESCOTT model, we do **not** use pLDDT confidence values, which is a measure identifying the confidence on the 3D structure of the protein. In ESCOTT/PRESCOTT, we consider the 2D structure of the protein and we evaluate a residue to belong to a “structured” region with Biotite Python package (77) and DSSP (78), through which we assign a secondary structure to each amino acid in the protein structure. Hence, given a structure, we use the notion of coiled structure vs uncoiled one, defined, these latter, as a stretch of unstructured residues longer than 5aa. This step is done to determine which are the uncoiled regions to be evaluated/scored based on T_JET_ only, excluding CV and PC scores. This is better explained in **pp 7**.

We have added a **new analysis of unstructured regions based on pLDDT**, as suggested by the reviewer, which is presented in the **new Figure S6**. This analysis compares the performance of ESCOTT and AlphaMissense across all single point mutation DMS experiments. Overall, both analyses reveal a consistent trend: prediction performance is higher in structured regions compared to coiled regions (**Figure S5**) and higher in regions with high pLDDT compared to regions with low pLDDT (**Figure S6**). The trend is much more pronounced when using the pLDDT metric. This is expected, as the secondary structure-based metric used in ESCOTT includes regions classified as coiled but with high pLDDT confidence from AlphaFold. In contrast, the pLDDT metric better captures truly disordered regions, where predictions of mutational effects tend to be less accurate.

Using gnomAD to infer if variation is benign. Page 4: Line 22-23 "As a general rule, if a variant is common in one population, it can be assumed to be benign across all populations." Is not true for historical population bottlenecks that inflated variant frequencies in some populations (s.a. the Finnish) and it does not consider pathogenicity through recessive inheritance. The authors should clarify how PRESCOTT/ESCOTT, if at all, deals with pathogenicity through different modes of inheritence.

This point has been clarified in a **new section** “ESCOTT and PRESCOTT on unique genetic heritage”, pp 11, where **a new analysis** of pathogenic variants, collectively known as the Finnish Disease Heritage (FDH), has been conducted with ESCOTT, PRESCOTT and AlphaMissense. See the answer to the first referee that had a similar concern. We provide the results in a **new Table S1 as a supplementary material**.

We also added **a new section** “ESCOTT, PRESCOTT, iGEMME and AlphaMissense on recessive and dominant inheritance modes” where we discussed the analysis of two new proteins, STAT1 (P42224) and PTPN11 (Q06124), that have been considered previously in

Hijikata, Atsushi, et al. "Decoding disease-causing mechanisms of missense mutations from supramolecular structures." *Scientific reports* 7.1 (2017): 8541.

to study the correlation of inheritance mode and 3D structure.

23 STAT1 mutations with demonstrated pathogenic effect have been classified in three modes: autosomal recessive (AR), autosomal dominant (AD), and gain-of-function (GF):

| **Mutation** | **Mode** | **ESCOTT** | **PRESCOTT** | **AlphaMissense** | **iGEMME** |
| --- | --- | --- | --- | --- | --- |
| D165H | GF | 0.55 | 0.55 | 0.957 | 0.58 |
| D165G | GF | 0.55 | 0.55 | 0.905 | 0.58 |
| Y170N | GF | 0.86 | 0.86 | 0.725 | 0.86 |
| C174R | GF | 0.27 | 0.27 | 0.203 | 0.27 |
| N179K | GF | 0.24 | 0.24 | 0.657 | 0.23 |
| M202V | GF | 0.18 | 0.18 | 0.386 | 0.17 |
| A267V | GF | 0.5 | 0.5 | 0.8919 | 0.49 |
| Q271P | GF | 0.81 | 0.81 | 0.96 | 0.83 |
| R274Q | GF | 0.56 | 0.56 | 0.326 | 0.53 |
| R274G | GF | 0.68 | 0.68 | 0.851 | 0.66 |
| R274W | GF | 0.58 | 0.58 | 0.527 | 0.56 |
| K278E | GF | 0.31 | 0.31 | 0.61 | 0.36 |
| Q285R | GF | 0.38 | 0.38 | 0.325 | 0.49 |
| K286I | GF | 0.48 | 0.48 | 0.964 | 0.56 |
| T288A | GF | 0.15 | 0.15 | 0.226 | 0.19 |
| E320Q | AD | 0.55 | 0.55 | 0.979 | 0.59 |
| G384D | GF | 0.54 | 0.54 | 0.99 | 0.68 |
| T385M | GF | 0.82 | 0.82 | 0.68 | 0.89 |
| Q463H | AD | 0.82 | 0.82 | 0.999 | 0.9 |
| L600P | AR | 0.99 | 0.99 | 0.999 | 1.0 |
| K637R | AD | 0.06 | 0.06 | 0.085 | 0.08 |
| K637E | AD | 0.23 | 0.23 | 0.749 | 0.32 |
| L706S | AD | 0.56 | 0.56 | 0.999 | 0.55 |

Dark yellow: pathogenic; light yellow: ambiguous; white: benign.

58 PTPN11 mutations with pathogenic effect have been classified in two modes: dominant negative (DN) and gain-of-function (GF):

| **Mutation** | **Mode** | **ESCOTT** | **PRESCOTT** | **AlphaMissense** | **iGEMME** |
| --- | --- | --- | --- | --- | --- |
| T42A | GF | 0.38 | 0.38 | 0.973 | 0.33 |
| N58D | GF | 0.59 | 0.59 | 0.994 | 0.52 |
| N58H | GF | 0.46 | 0.46 | 0.978 | 0.41 |
| N58K | GF | 0.55 | 0.55 | 0.999 | 0.48 |
| D61N | GF | 0.13 | 0.13 | 0.994 | 0.13 |
| D61G | GF | 0.52 | 0.52 | 0.999 | 0.43 |
| D61A | GF | 0.68 | 0.68 | 0.999 | 0.6 |
| Y62D | GF | 0.62 | 0.62 | 0.994 | 0.49 |
| Y62C | GF | 0.13 | 0.13 | 0.804 | 0.13 |
| Y63C | GF | 0.39 | 0.39 | 0.975 | 0.35 |
| E69Q | GF | 0.44 | 0.44 | 0.990 | 0.43 |
| A72S | GF | 0.35 | 0.35 | 0.916 | 0.29 |
| A72G | GF | 0.7 | 0.7 | 0.993 | 0.6 |
| E76D | GF | 0.44 | 0.44 | 0.998 | 0.4 |
| Q79R | GF | 0.44 | 0.44 | 0.964 | 0.4 |
| D106A | GF | 0.45 | 0.45 | 0.985 | 0.41 |
| E139D | GF | 0.42 | 0.42 | 0.988 | 0.44 |
| Q256R | GF | 0.4 | 0.4 | 0.894 | 0.39 |
| L261F | GF | 0.18 | 0.18 | 0.507 | 0.18 |
| L261H | GF | 0.31 | 0.31 | 0.507 | 0.27 |
| L262R | GF | 0.45 | 0.45 | 0.926 | 0.44 |
| R265Q | GF | 0.19 | 0.19 | 0.994 | 0.24 |
| Y279S | DN | 0.86 | 0.86 | 0.999 | 0.88 |
| I282V | GF | 0.57 | 0.57 | 0.981 | 0.58 |
| F285S | GF | 0.89 | 0.89 | 0.999 | 0.88 |
| F285L | GF | 0.78 | 0.78 | 0.999 | 0.8 |
| N308D | GF | 0.76 | 0.76 | 0.996 | 0.78 |
| N308S | GF | 0.33 | 0.33 | 0.518 | 0.35 |
| G409A | GF | 0.15 | 0.15 | 0.261 | 0.19 |
| T411M | GF | 0.12 | 0.12 | 0.065 | 0.12 |
| A461T | GF | 0.16 | 0.16 | 0.999 | 0.2 |
| A461S | DN | 0.52 | 0.52 | 0.972 | 0.58 |
| G464A | DN | 0.91 | 0.91 | 0.999 | 0.92 |
| T468M | DN | 0.83 | 0.83 | 0.994 | 0.84 |
| T468P | DN | 0.99 | 0.99 | 0.996 | 0.99 |
| P491T | GF | 0.49 | 0.49 | 0.896 | 0.5 |
| P491S | GF | 0.29 | 0.29 | 0.798 | 0.27 |
| P491H | GF | 0.17 | 0.17 | 0.961 | 0.16 |
| P491L | GF | 0.28 | 0.28 | 0.943 | 0.27 |
| R498W | DN | 0.92 | 0.92 | 0.999 | 0.93 |
| R498L | DN | 0.94 | 0.94 | 0.999 | 0.95 |
| R501K | GF | 0.83 | 0.83 | 0.997 | 0.85 |
| S502T | GF | 0.69 | 0.69 | 0.984 | 0.68 |
| S502A | GF | 0.52 | 0.52 | 0.869 | 0.53 |
| S502L | GF | 0.51 | 0.51 | 0.998 | 0.51 |
| G503R | GF | 0.66 | 0.66 | 0.999 | 0.66 |
| G503E | GF | 0.21 | 0.21 | 0.999 | 0.24 |
| G503V | GF | 0.78 | 0.78 | 0.999 | 0.78 |
| G503A | GF | 0.72 | 0.72 | 0.998 | 0.72 |
| M504V | GF | 0.71 | 0.71 | 0.983 | 0.73 |
| Q506P | DN | 0.92 | 0.92 | 0.999 | 0.93 |
| T507K | DN | 0.43 | 0.43 | 0.996 | 0.49 |
| Q510R | DN | 0.92 | 0.92 | 0.999 | 0.93 |
| Q510E | DN | 0.76 | 0.76 | 0.943 | 0.81 |
| Q510P | DN | 0.96 | 0.96 | 0.998 | 0.96 |
| Q510H | DN | 0.91 | 0.91 | 0.999 | 0.92 |
| T553M | GF | 0.06 | 0 | 0.07 | 0.06 |
| L560F | GF | 0.1 | 0.02 | 0.125 | 0.12 |

Both case studies confirm the difficulty for by ESCOTT/PRESCOTT and AlphaMissense on classifying GF and AD mutations, while they highlight no mistake on the classification of DN and AR mutations. Systematic analyses should be done at large scale to assess anything on mode classification.

Interpretability of the ESCOTT/PRESCOTT scores. Figure 2 D&F: Unsure what the score scale means in D and F, between ESCOTT and PRESCOTT, would be very helpful for non-CS/Math oriented clinical experts to better describe this so they can understand it in context for their patients; e.g. what does a score of 0 or of 1 indicate PRESCOTT and ESCOTT? Is that scoring (0,1) different in the ESCOTT/PRESCOTT score scale? I am also unclear what table F exactly tries to convey because of this and cannot accurately understand from this table the impact of adding allele frequency on the scoring. Adding a row that show what you expect it to be for a perfect Mutation scoring would help here.

We thank the referee for this insightful remark, which has helped us **improve the text** (pp. 4–5). We have clarified the interpretability of the ESCOTT and PRESCOTT scores, explaining that their scale is the same, since PRESCOTT adjusts ESCOTT scores within the scale. We clarified that ESCOTT matrix was given with ranksorted scores:

“Note that the ESCOTT matrix in **Figure 2A** is based on ranksorted scores, ranging from 0 (no mutational effect) to 1 (high mutational effect). Consequently, the ESCOTT and PRESCOTT score scales are identical in this context (**Figure 2**, bottom left). In general, ESCOTT generates both a raw score matrix and a ranksorted score matrix, with PRESCOTT adjusting the ranksorted ESCOTT scores within the 0–1 scale (see Methods).”

Additionally, in the *Methods* section “ESCOTT ranksorted scores”, we wrote a new sentence:

“PRESCOTT scores are computed from the ranksorted ESCOTT matrix. If provided with the matrix of raw ESCOTT scores, PRESCOTT first ranksorts them before adjusting the values based on allele frequencies.”

In pp.6, we also explained how scores should be understood in the context of clinical applications by adding a paragraph discussing specific mutations that can be classified by PRESCOTT, potentially assisting doctors in evaluating ClinVar annotations.

To further address this concern, we **rewrote the legend** of **Figure 2** for clarity and proposed a **new coloring scheme for the table** in **Figure 2F** to make its connection to **Figures 2B** and **2D** more explicit. We believe these revisions will make the impact of adding allele frequency data on the scoring process more comprehensible, especially for non-CS/Math-oriented clinical experts, as mentioned.

I have the following minor comments:

*    The manuscript could use an overall restructuring for better readability. the dataset, model development, and comparisons to other computational methods are scattered throughout the manuscript rather than being organized in fewer, more focused sections and PRESCOTT and it's capabilities is mentioned very often and need not be repeated so often. A larger font in the figures (on y/x axis and text throughout) could also greatly improve the readability.

We rewrote the Results section to incorporate parts of the Methods, following your suggestion, and we agree that this has significantly improved the presentation of the methods. Additionally, we chose to retain the comparisons with other tools within the specific sections analyzing the various datasets, as this approach avoids redundancy in discussing PRESCOTT's dataset analysis.

**Figure 8** was particularly difficult to read and we augmented the characters. The same adjustment was applied to **Figure 4**.

*    Please add the UniProt identifiers to their respective protein to the gain of function mutations .csv file so no confusion can be caused regarding isoform/canonical sequence should future researchers require this for comparison.

This is done. See **Figure 8F** with the labels included.

*    ESCOTT is mentioned that it can be applied to splicing variants in line 61 page 4, but thereafter no mention is made on how ESCOTT can help with evaluating such variants.

The **new section** “ESCOTT/PRESCOTT can differentiate mutational effects across isoforms”, pp 10, is dedicated to this point. Please, see the answer to the comment of the first referee on splicing variant. We also added a **new Figure 5C** where a comparative study between isoform 1 and isoform 8 of P53 is developed.

*    Page 13, the note on ClinVar is a bit confusing and seems to indicate that PRESCOTT cannot deal with One-Based Vs. Zero-Based Genome Coordinate Systems, but could also mean this is only for the sake of comparison to EVE? Clarification would help here. 

The November 2021 ClinVar report on these mutations is used in our analysis to align with the version employed by EVE, ensuring a consistent basis for comparison. We rewrote the sentence.

*    Use of word doctors throughout manuscript (p4 line 14 and p16 line 10) where the authors are referring to target users. I believe a better word for inclusivity would be clinicians (as, worldwide, not all GCs or clinical researchers are also MDs/doctors)

The word “doctor” has been replaced in all the manuscript with “clinician”.

*    Minor Docker/gitlab issues: I could not get the docker / gitlab make a test-run in a fluent way. There were a few minor issues in the way. Firstly, There's no latest tag in dockerhub, making ` docker pull tekpinar/prescott-docker` or ` docker pull tekpinar/prescott-docker:latest` result in an error. Would recommend to add a `latest` tag in dockerhub and specify the version within the manuscript that was used for the results (I think. v.1.6.0?). Had to use `docker pull tekpinar/prescott-docker:v1.6.0`. Next I cloned the repository from gitlab but it would be helpful to add Docker details for end users to run your example. Instead of the command `prescott -e ../data/MLH1_normPred_evolCombi.txt -g ../data/gnomAD_v4.0.0_MLH1_HUMAN_ENSG00000076242.csv -s ../data/MLH1.fasta` I had to modify the docker run executed within the cloned PRESCOTT project as 
`docker run --mount src="$(pwd)/data",target=/data,type=bind tekpinar/prescott-docker:v1.6.0 prescott -e /data/MLH1_normPred_evolCombi.txt -g /data/gnomAD_v4.0.0_MLH1_HUMAN_ENSG00000076242.csv -s /data/MLH1.fasta`. Would like to note this is cherry-on-top type of a minor comment, the authors really did an excellent job on their documentation and reproducibility.

This has been fixed. Thank you.

*    Page 15, line 22: states Mustafa - likely a typo Based on author's name.

Done, thank you!

Reviewer's Responses to Questions

**Second round of review**

**Reviewer 1**

I have carefully reviewed the responses, and the authors have addressed my comments well. I have no further concerns and would like to submit an Accept recommendation.

**Reviewer 2**

The authors have addressed all my comments. I applaud all their efforts which have significantly strengthened how to interpret PRESCOTT/ESCOTT scores.

I have one last minor comment that the file “gain-of-function-with-igemme-scores-2.csv” still has the column “protein” and that does not display the UniProt identifier. I would request of the authors to add these identifiers for the final print.

I have no further comments. I believe that, with the current revisions, the manuscript is in an acceptable state for publication.
